# Navigating Nitration Chemistry: A Practical Guide to Reagents, Mechanisms, and Selectivity

**DOI:** 10.1002/anie.202526128

**Published:** 2026-03-25

**Authors:** Harry Lecomte, Anthony J. Fernandes, Dmitry Katayev

**Affiliations:** ^1^ Department Für Chemie und Biochemie Universität Bern Bern Switzerland

**Keywords:** electrophilic, mechanisms, nitration, radical, reagents

## Abstract

We highlight recent advances in nitration chemistry with emphasis on the development of sustainable and selective methodologies. A comprehensive overview of nitrating reagents is provided, classified by origin (organic or inorganic) and activation mode (photochemical, electrochemical, thermal, and others). Each reagent is critically analyzed with respect to its performance across different nitration processes, that is, aromatic, *ipso*‐, olefinic, alkyne, and heteroatom nitration. This analysis scrutinizes yield, substrate scope, functional group tolerance, versatility, resource and hazard, key features that comprise an efficiency score. A comparative analysis of activation strategies underscores the evolution of nitration from classical mixed‐acid approaches to modern photocatalytic, electrochemical, and cross‐coupling methodologies. The insights gathered here provide a practical framework for identifying the most suitable nitrating reagents and highlight future directions toward safer, greener, and more versatile nitration chemistry.

## Graphical abstract

This review highlights key contributions of modern nitration chemistry, emphasizing sustainable mechanistic platforms and comparing the performance of both organic and inorganic reagents across aromatic, *ipso‐*, olefin, alkyne, and heteroatom nitration. It provides the community with a clearer, unified perspective on current advances and facilitates the selection of nitrating reagents by establishing a performance score.

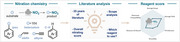

## 1 Introduction

**FIGURE 1:**
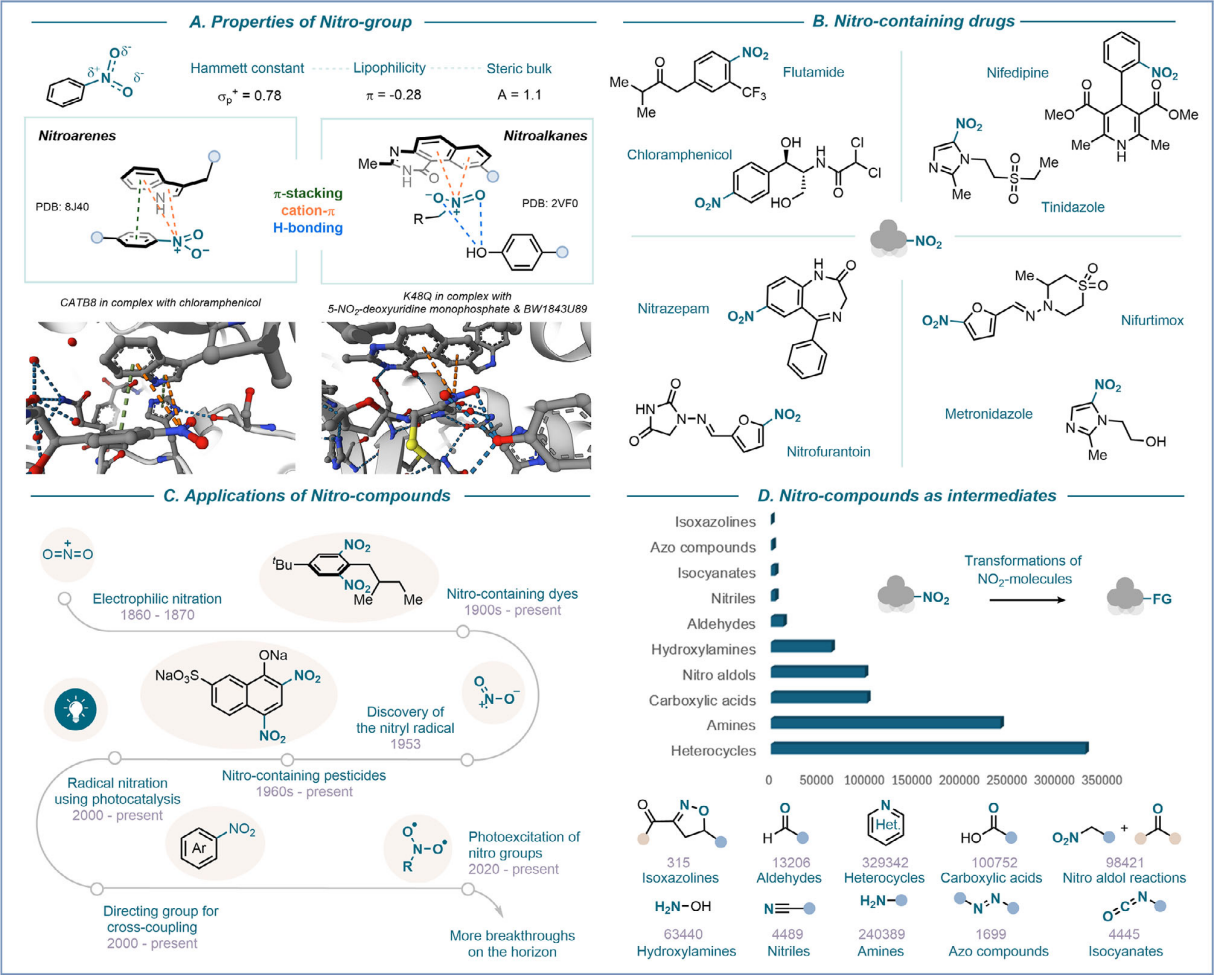
The importance of the nitro group: (a) key properties and interactions involving ‐NO_2_ groups, (b) selected NO_2_‐containing drugs, (c) applications over the years, and (d) as a key intermediate to access other functionalities (data obtained from SciFinder searches, 2025).

Nitro‐containing compounds occupy a central place in chemistry and have shaped both industrial practice and academic research for more than a century [1, 2, 3, 4]. The unique properties of the NO_2_ group (relatively small, hydrophilic, and a strong electron‐withdrawing substituent) make these derivatives ubiquitous as pharmaceuticals, agrochemicals, dyes, energetic materials, and fine chemicals [5, 6, 7]. The nitro group itself can engage in diverse non‐covalent interactions, such as hydrogen bonding, π‐stacking, and cation‐π interactions, which underpin its success in drug design and molecular recognition (Figure 1a,b).

Historically, nitration chemistry has been dominated by electrophilic pathways, with the so‐called “mixed‐acid” process becoming the cornerstone of large‐scale industrial synthesis (Figure 1c) [4]. While efficient, this method is associated with severe drawbacks, including poor selectivity, functional group intolerance, and significant environmental and safety concerns. The 20th century witnessed the emergence of radical pathways that harnessed the previously elusive nitryl radical, thereby expanding the conceptual space of nitration [8, 9]. More recently, developments in photochemistry, electrochemistry, and flow technology have sparked renewed interest in nitration chemistry, providing milder, more sustainable, and more selective alternatives. Despite these advances, major challenges remain. Traditional methods are still widely used, often at the expense of greener and safer alternatives. Regioselectivity still is a persistent issue, particularly in complex molecules. Alongside these challenges, nitration chemistry offers exciting opportunities, and the scientific community is continually innovating to develop novel methodologies to help address these limitations. Emerging methods such as metal catalysis, organocatalysis, dual catalytic strategies, electrochemistry, and light‐driven pathways are reshaping the field [1, 4].

Applications are also broadening, with nitro groups being exploited not only as pharmaceuticals and agrochemicals but also in material sciences, organic electronics, and novel synthetic methodologies [10, 11, 12, 13, 14]. Beyond their applications, nitro groups serve as highly versatile synthetic intermediates, enabling access to a wide range of functional derivatives and greatly expanding their chemical utility. Among these transformations, the conversion of nitro compounds into heterocycles and amines represents the most extensively exploited pathway, surpassing other valuable transformations enabling the access to carboxylic acids, hydroxylamines, and related derivatives (Figure 1d).

**FIGURE 2:**
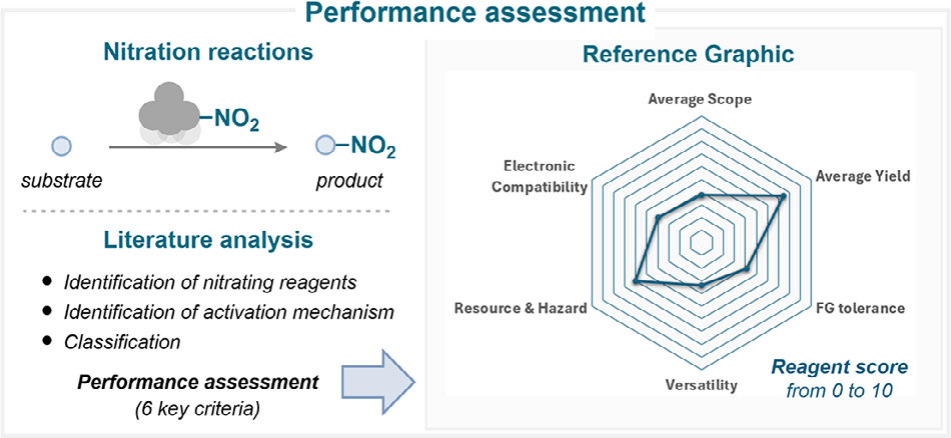
Approach for assessing the performance of nitrating reagents.

**FIGURE 3:**
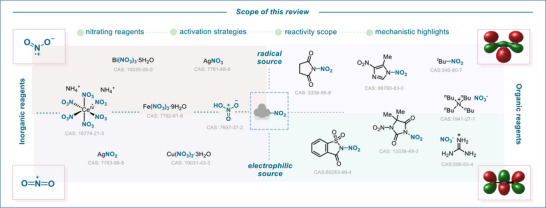
Scope of this review: reagents employed in modern nitration chemistry, involving both electrophilic and radical approaches.

Many recent reviews of nitration chemistry are available in the literature, providing a useful overview of the field [1, 2, 3, 4, 6, 15, 16]. In fact, this domain is so vast, encompassing hundreds of nitrating reagents and reaction conditions, that it is overwhelmingly challenging to appropriately select the required reaction conditions (Figure 2). Consequently, the present review aims to provide a critical assessment of the literature, rather than providing a compilation of examples. Hence, all nitrating reagents discussed in this review are systematically analyzed and classified according to valuable criteria (Figure 3; see Supporting Information, page 2, for further details), including:

- **Average Scope**: The number of successfully nitrated substrates was counted for each reagent, and an average was calculated. This metric provides an objective measure of how widely a nitrating reagent has been used, helping chemists assess its overall usefulness across different structural classes.
- **Electronic Compatibility**: The types of substrates nitrated (electron‐rich, electron‐poor, neutral nature, and/or heteroaromatics) were evaluated to determine the breadth of applicability. This measure reflects a nitration method's ability to accommodate a wide range of electronic environments, indicating its utility across diverse synthetic challenges.
- **Average Yield**: Similarly, average yields across reported examples were compiled. Yield remains a central criterion in evaluating reaction efficiency; comparing average yields across reagents offers insight into which methods consistently deliver synthetically valuable results.
- **Functional Group Tolerance**: Substrate scopes were analyzed for the presence of sensitive functional groups, such as alcohols, amines, amides, nitriles, carboxylic acids, esters, aldehydes, phenols, anilines, and various heterocycles. This parameter is crucial for determining the chemoselectivity and practicality of a nitration method in complex molecule synthesis, especially in late‐stage functionalization and medicinal chemistry.
- **Versatility**: Each reagent was assessed based on the number of distinct mechanisms it participates in and the diversity of nitrating species it can generate. Versatile reagents offer flexibility in synthetic planning by enabling multiple pathways or reactivities, making them valuable tools in a chemist's repertoire.
- **Resource & Hazard**: A score was established based on reagent toxicity, cost, and the potential for recovery or recycling. Incorporating these parameters enables practitioners to evaluate reagents beyond intrinsic reactivity metrics, thereby facilitating more informed selection based on practical, economic, and environmental considerations.

Based on these criteria, each reagent is assigned a score of 0 to 10 (with 10 being the highest) to assess its performance and provide a clearer overview of the most suitable reagents for each nitration reaction discussed. While this score represents a comparative and literature‐based assessment derived from the available data, it reflects the current state of the literature and may evolve as additional studies emerge.

The substrate scope of key nitration reactions is examined, including aromatic, *ipso*‐, alkene, alkyne, and heteroatom nitration reactions, while highlighting activation modes ranging from thermal and photochemical to electrochemical or mechanical strategies. Special emphasis is placed on emerging methodologies that are redefining modern nitration chemistry, and representative examples are illustrated in each section. Other contributions are cited in the discussion and compiled in a summarizing scheme, providing an overview of the given nitration reaction, including selected examples of nitrated products to showcase their diversity. In this section of the review, we aim to provide guidance on selecting the most appropriate nitrating reagent for a given transformation.

Finally, this review provides a comprehensive and practical guide to nitration chemistry, structured around the scores assigned to nitrating reagents as well as the underlying mechanistic strategies and methodological concepts.

## 2 *ipso*‐Nitration

### 2.1 Metal‐Catalyzed Nitration

**SCHEME 1:**
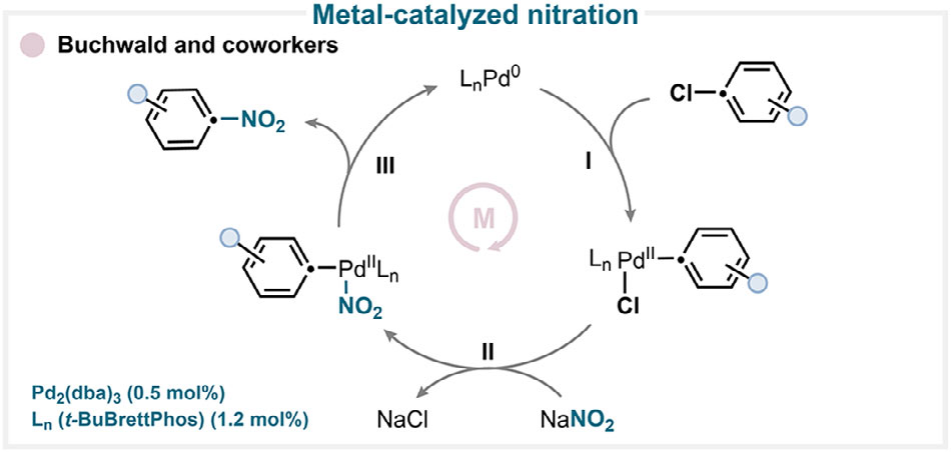
Key metal‐catalyzed *ipso*‐nitration mechanism proposed by Buchwald and coworkers [17]. t‐BuBrettPhos = 2‐(di‐tert‐butylphosphino)‐2′,4′,6′‐triisopropyl‐3,6‐dimethoxy‐1,1′‐biphenyl.

A representative example of metal‐catalyzed *ipso*‐nitration was demonstrated by Buchwald and coworkers, who employed Pd_2_(dba)_3_ as the catalyst and sodium nitrite (NaNO_2_) as the nitrating source (Scheme 1) [17]. The proposed mechanism involves an initial oxidative addition (**I**) of the aryl chloride to the palladium center, followed by a transmetallation step (**II**) with sodium nitrite, and finally reductive elimination (**III**) to regenerate the catalyst and yield the desired nitroarene (for key examples, see Scheme 7c) [17].

Comparable results have been obtained using sodium nitrite [17, 18, 19, 20, 21], as well as other nitrating reagents such as potassium nitrite (KNO_2_) [22], or *tert*‐butylammonium nitrite (TBAN) [19, 23], in combination with either palladium or copper catalysts. Although these mechanisms have not been investigated in detail, it is generally suggested that the reaction proceeds via an inner‐sphere catalytic process [24].

### 2.2 In Situ Activation

**SCHEME 2:**
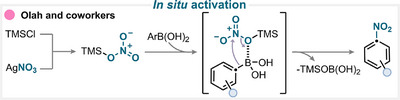
Key ipso‐nitration of boronic acids reported by Olah and coworkers [25].

Olah and coworkers demonstrated a complementary approach to perform *ipso*‐nitration of aryl boronic acids using silver nitrate (AgNO_3_) (Scheme 2) [25]. In this protocol, the nitrating reagent reacts with trimethylsilyl chloride (TMSCl) to generate in situ a silyl nitrate intermediate. This active species subsequently reacts with the arylboronic acid via a 1,3‐metalate rearrangement, producing the desired nitro arene (for key examples, see Scheme 7c) [25]. Following a similar strategy, the same group has reported the use of sodium nitrite and TMSCl to generate silyl nitrite species that enable both nitrosation and nitration of arylboronic acid derivatives [26].

Prior to these findings, the Olah research group had already reported a related protocol employing tetrabutylammonium nitrate in combination with trifluoroacetic anhydride to form trifluoroacetyl nitrate as an active nitrating species [27]. Building on this foundation, Caille and coworkers used a similar pathway using the same reagent [28].

Other non‐traditional approaches have also been documented, such as the use of zirconyl nitrate in combination with molecular iodine or iron(III) nitrate with ZnFe_2_O_4_. Despite their different activation systems, these methods share a common mechanistic principle: the in situ generation of an active nitrating species that subsequently engages in *ipso*‐nitration of the aryl boronic acid substrate. Such “non‐orthodox” systems highlight ongoing exploration of heterogeneous or metal‐oxide‐assisted protocols to improve efficiency, selectivity, and sustainability in nitration chemistry [29, 30].

### 2.3 Light‐Mediated Nitration

**SCHEME 3:**
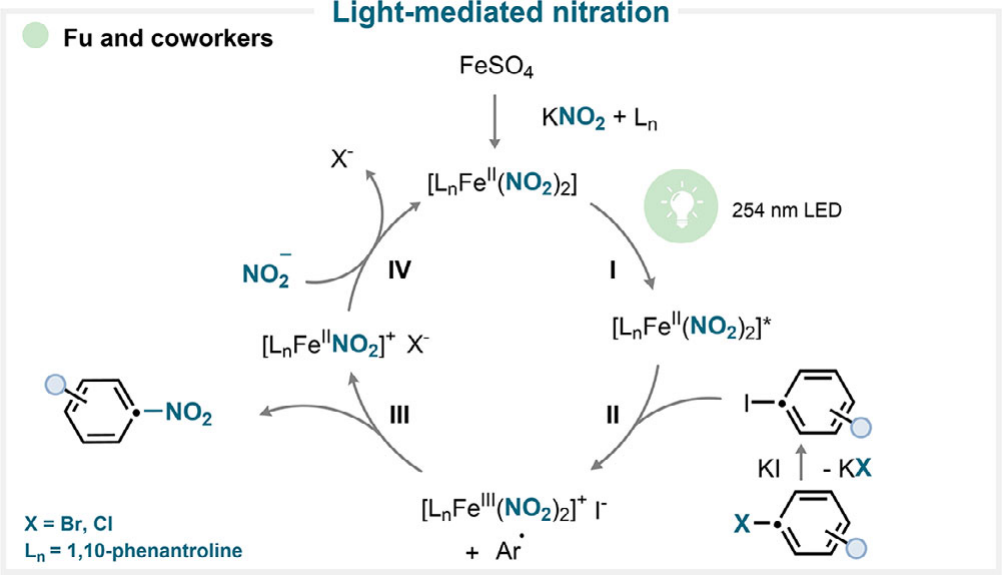
Key proposed mechanism of photochemically driven ipso‐nitration by Fu and coworkers [31].

In Fu's work, iron(II) sulfate forms an iron(II) nitrite complex in the presence of KNO_2_ and 1,10‐phenanthroline (Scheme 3) [31]. Under light irradiation, the complex is promoted to an excited state. A subsequent single‐electron transfer (SET) to the in situ‐generated aryl iodide generates the iron(III) species and an aryl radical. The latter then reacts with the iron(III) complex, yielding the desired nitro‐substituted compound, and after anion exchange, regenerating the iron(II) catalyst (for key examples, see Scheme 7c) [31].

In contrast, Jin and coworkers reported a different strategy employing iron(III) nitrate [32]. Upon light irradiation, the excited ferric complex undergoes N–O bond fragmentation and generates a nitryl radical and an Fe(IV) = O complex. The electrophilic nitryl radical then adds to the arylboronic acid, forming a cyclohexadienyl radical intermediate [33, 34]. Subsequent SET oxidation and deboronation yield the desired nitrated product [32].

### 2.4 Acid‐Assisted Nitration

**SCHEME 4:**
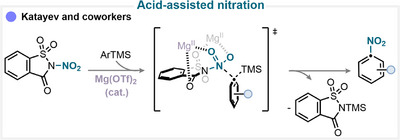
Key proposed mechanism of acid‐assisted ipso‐nitration by Katayev and coworkers [35].

In this study, our group introduced an efficient protocol for the *ipso*‐nitration of organosilanes using the electrophilic *N*‐nitrosaccharin reagent in the presence of the magnesium triflate Lewis acid catalyst (Scheme 4) [35]. The mechanism involves the coordination of the Lewis acid to the nitrating reagent, which significantly weakens the N–NO_2_ bond. This activation facilitates the formation of a nitronium‐like species capable of delivering the NO_2_ group to the organosilane substrate via a concerted *ipso*‐substitution mechanism (for key examples, see Scheme 7c). Experimental and computational investigations, including Hammett analysis and density functional theory (DFT) calculations, supported this mechanistic proposal and confirmed the electrophilic nature of the activated *N*‐nitrosaccharin species [35].

Similarly, *ipso*‐nitration can be achieved via an acid‐assisted pathway using hexafluoroisopropanol (HFIP) as the solvent. Owing to its strong hydrogen‐bond donating ability and high polarity, HFIP can stabilize cationic or radical intermediates and facilitate the generation of the active nitrating species [36]. When used in combination with iron(III) nitrate or *N*‐nitrosaccharin, this medium promotes efficient and smooth *ipso*‐substitution on aromatic substrates [35, 37].

### 2.5 Photoredox Catalysis

**SCHEME 5:**
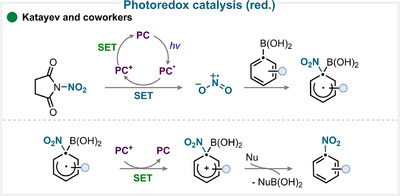
Key proposed mechanism of photoredox‐catalyzed ipso‐nitration by Katayev and coworkers [38].

Using *N*‐nitrosuccinimide under photoredox conditions, our group successfully developed an *ipso*‐nitration of aryl boronic acid derivatives (Scheme 5) [38]. Building on this foundation, Schoenebeck and coworkers later extended the strategy to aryl germanium species, demonstrating that the method not only retained high efficiency but also exhibited remarkable selectivity depending on the electronic nature of the substrate [39].

In both studies, an Ru‐based photocatalyst was employed. Upon blue‐light irradiation, the photocatalyst is promoted to an excited state and undergoes oxidative quenching with *N*‐nitrosuccinimide, generating the nitryl radical. This electrophilic radical then reacts at the *ipso*‐position of the aryl substrate, forming an aryl radical intermediate. Subsequent oxidation and rearomatization yield the nitrated aromatic product while regenerating the photocatalyst (for key examples, see Scheme 7c). DFT calculations supported this mechanistic proposal, confirming the key role of the nitryl radical as the active nitrating species and validating the reaction pathway observed experimentally [38, 39].

### 2.6 Thermal Activation

The general mechanism relies on the use of thermolabile nitrating reagents that, upon heating, decompose to generate an active nitrating species, typically the nitryl radical or the nitronium ion. This well‐established strategy has been successfully demonstrated using a broad range of reagents, such as silver nitrate [41], bismuth nitrate [42, 43, 44], *tert*‐butyl nitrite (TBN) [45, 46], nitric acid [28, 47], iron nitrate [29, 40, 48], sodium nitrite [49], nitronium tetrafluoroborate (NO_2_BF_4_) [50, 51], and 1,3‐disulfonic acid imidazolium nitrate (DsimNO_3_) [52].

**SCHEME 6:**
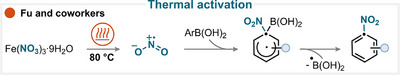
Key proposed mechanism of thermally activated ipso‐nitration by Fu and coworkers [40].

**SCHEME 7:**
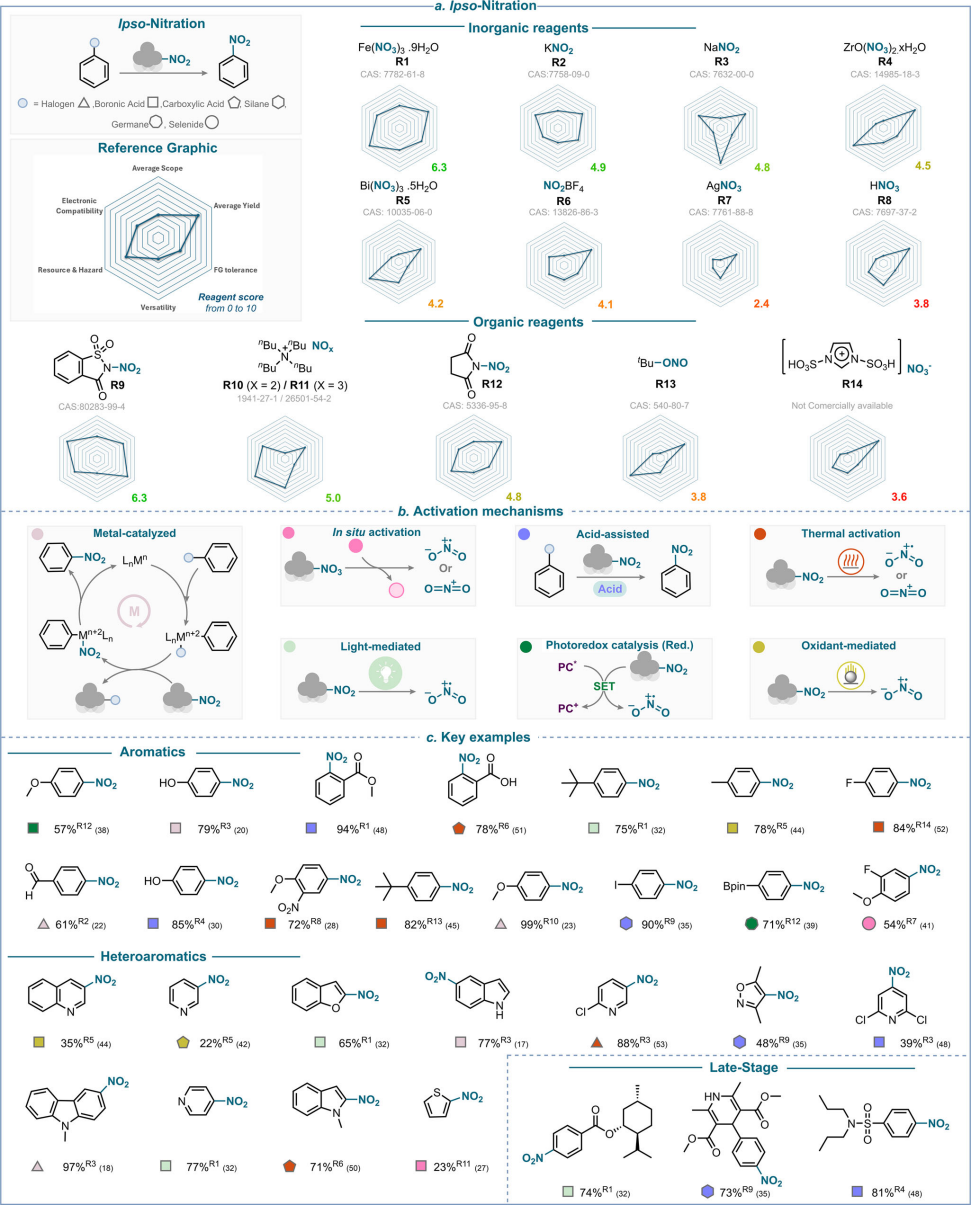
Overview of ipso‐nitration strategies over the past 25 years. (a) Nitrating reagents used, ranked from best to least performing, and divided between organic and inorganic natures. (b) Activation mechanisms encountered, (c) Selected examples using aromatic, heteroaromatic, and late‐stage derivatives. The colored balls indicate the activation method used, and the superscript number after the yield refers to the nitrating reagent used. The reference is given in brackets.

A representative example of this thermal activation pathway was reported by Fu and coworkers, who employed iron(III) nitrate at 80°C to generate a nitryl radical (Scheme 6), which undergoes *ipso*‐addition to the arylboronic acid substrate, followed by release of the boronic acid‐derived radical, ultimately affording the desired nitroarene product (for key examples, see Scheme 7c).

This work illustrates how simple thermal activation can enable efficient and selective *ipso*‐nitration without requiring external oxidants or photochemical assistance [40].

Alternatively, electrophilic diaryliodonium salts react with sodium nitrite to afford nitroarenes via thermally allowed intramolecular rearrangements [53].

### 2.7 Oxidant‐Mediated Nitration

**SCHEME 8:**
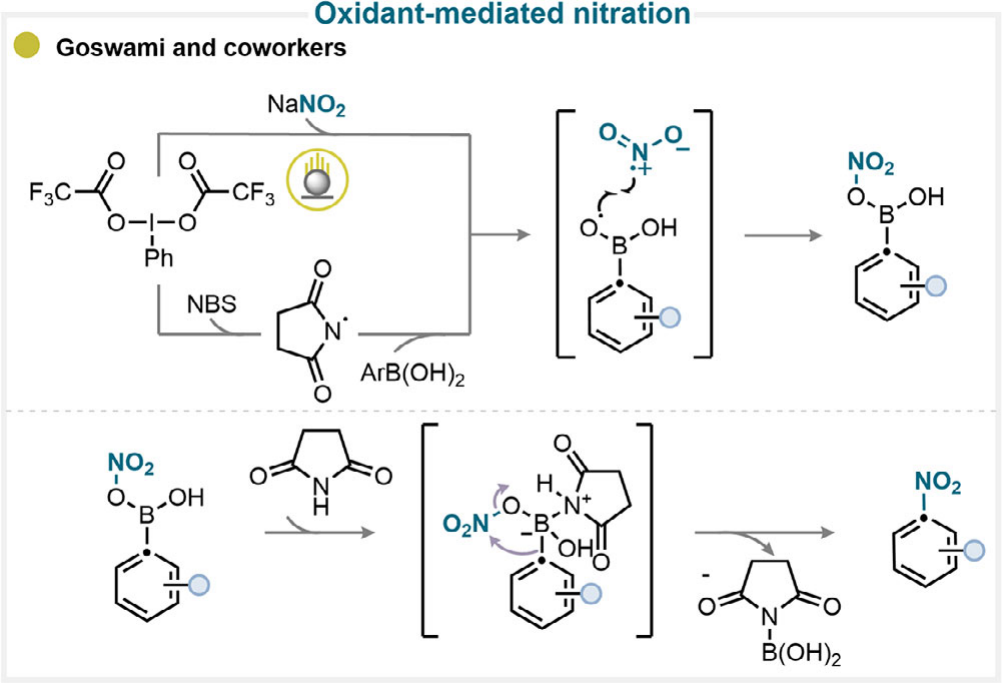
Key proposed mechanism of oxidant‐mediated ipso‐nitration by Goswami and coworkers [54].

Goswami and coworkers reported an elegant *ipso*‐nitration strategy that employs a dual‐oxidant system combining *N*‐bromosuccinimide (NBS) and [bis(trifluoroacetoxy)iodo]benzene (PIFA) [54]. In their study, PIFA plays two key roles in generating the succinimidyl radical, which is proposed to promote HAT with the arylboronic acid to form an oxygen‐centered radical and nitryl radical (through SET oxidation of sodium nitrite) (Scheme 8). Following radical–radical coupling, coordination of the *N*‐succinimide by‐product forms a tetracoordinated borate intermediate, which triggers a 1,3‐aryl migration leading to the product (for key examples, see Scheme 7c). This transformation highlights the synergistic effect of dual oxidation in enabling selective *ipso*‐nitration under mild conditions [54].

Similarly, bismuth nitrate has been employed in combination with potassium persulfate (K_2_S_2_O_8_) and proposed to generate both nitryl and aryl radicals (from boronic acids). The resulting radicals then recombine to furnish the corresponding nitroarene (for key examples, see Scheme 7c) [44].

### 2.8 Overview of the *ipso*‐Nitration

The assessment of the efficiency of various reagents with respect to the key criteria listed earlier (see the introduction and Supporting Information, page 2, for further details), is presented in Scheme 7. This analysis reveals that iron nitrate (**R1**) and *N*‐nitrosaccharin (**R9**) are the top performers in *ipso*‐nitration with a score of 6.3. The scores obtained for each criterion are well balanced for these two reagents, although a key advantage of **R1** over **R9** might lie in its *versatility*. Indeed, **R1** can operate under many mechanistic manifolds to release either a nitryl radical or a nitronium ion, whereas activation of **R9** remains, so far, limited to thermal and acid strategies.

Among other top performers, inorganic salts such as potassium nitrite (**R2**) and sodium nitrite (**R3**), and organic reagents such as TBA nitrite and nitrate (**R10**/**R11**) and *N*‐nitrosuccinimide (**R12**) show overall good performance.

## 3 Alkyne Nitration

### 3.1 Thermal Activation

The conventional strategy for thermally induced nitration of alkyne relies on thermolabile nitrating reagents, which decompose upon heating to generate nitryl radical. After subsequent addition, a vinyl radical intermediate is formed and further reacts to afford a functionalized nitroalkene product. Such transformations have been successfully demonstrated using a variety of metal nitrates, including iron(III) nitrate nonahydrate [56], copper(II) nitrate trihydrate [57], and aluminum nitrate nonahydrate [58]. A notable exception is TBN, which, upon heating, primarily releases nitric oxide [55, 59, 60, 61, 62, 63, 64, 65, 66]. The latter can then be oxidized in situ, typically by molecular oxygen, to generate the active nitryl radical. Because the key activation event depends on thermal energy, even when a secondary oxidant is involved, these processes are collectively classified as thermally activated nitration reactions.

**SCHEME 9:**
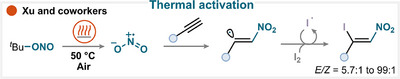
Key mechanistic proposal for the thermally activated nitration of alkynes by Xu and coworkers [55].

In the work of Xu and coworkers, the generation of nitryl radical was realized using TBN at 50°C under an air atmosphere (Scheme 9) [55]. The radical subsequently added to the alkyne, producing a vinyl radical intermediate, which was trapped by iodine, leading to the formation of the iodo‐nitroalkene via a difunctionalization pathway (for key examples, see Scheme 11c). In a related study, the Guo group employed a similar strategy, whereby the vinyl radical underwent intramolecular cyclization onto a tethered aromatic ring, followed by oxidation with iron salts, ultimately affording a cyclized nitroaromatic product (for key examples, see Scheme 11c) [56].

### 3.2 In Situ Activation

In situ‐generated nitrating species are usually produced by reacting a nitrate or nitrite salt with an activation partner, such as potassium iodide or trimethylsilyl chloride.

**SCHEME 10:**
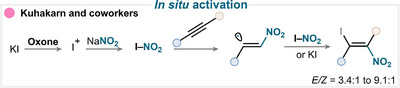
Key mechanistic proposal for in situ‐activated alkyne nitration by Kuhakarn and coworkers [67].

**SCHEME 11:**
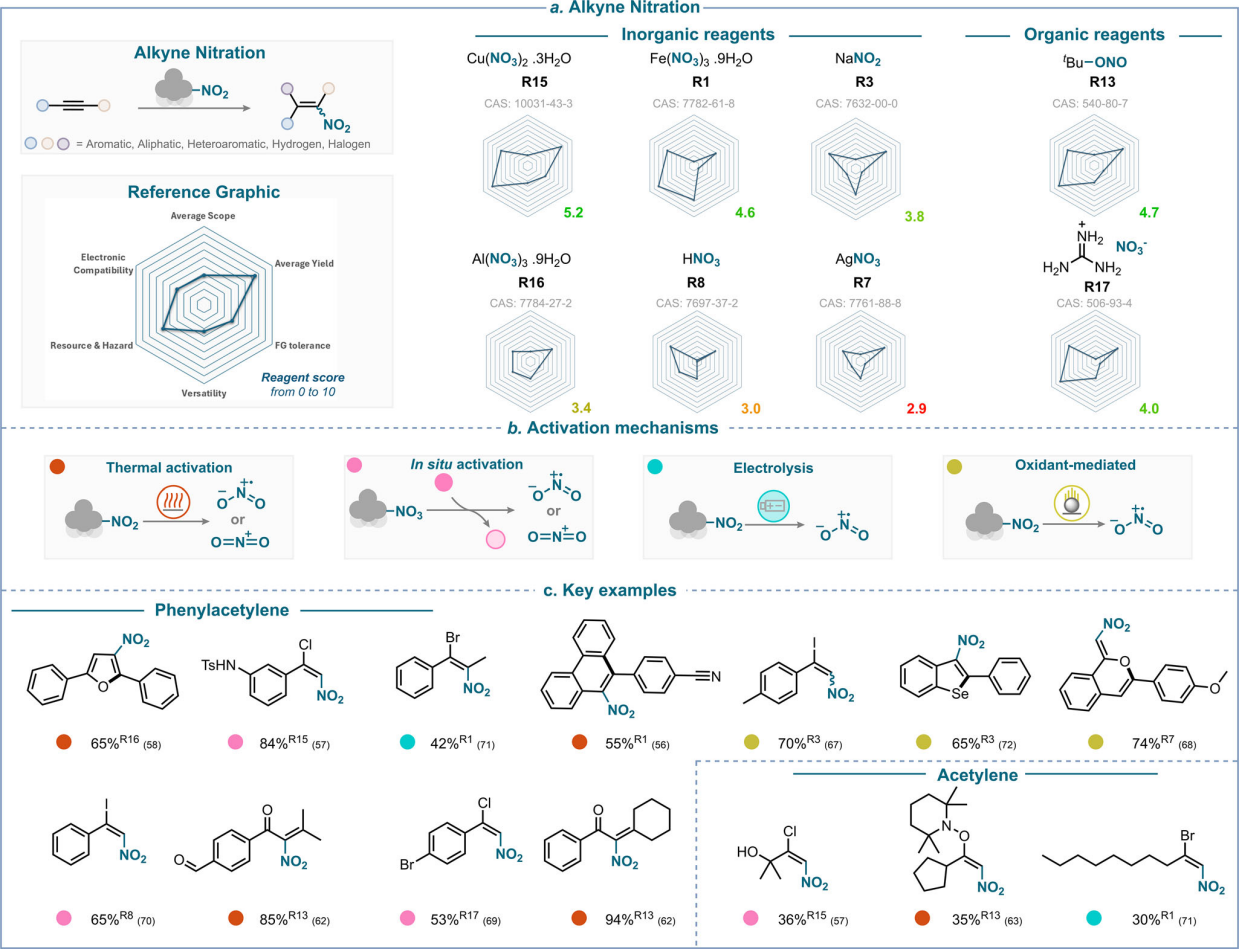
Overview of alkyne nitration strategies over the past 25 years. (a) Nitrating reagents used, ranked from best to least performing, and divided between organic and inorganic natures. (b) Activation mechanisms encountered. (c) Selected examples using phenylacetylene and acetylene derivatives. The colored balls indicate the activation method used, and the superscript number after the yield refers to the nitrating reagent used. The reference is given in brackets.

A representative example of this methodology is provided by the work of Kuhakarn and coworkers, who employed sodium nitrite as the nitrating reagent in combination with Oxone and potassium iodide (KI) (Scheme 10) [67]. In their proposed mechanism, KI is first oxidized by Oxone to form electrophilic iodine species (I^+^), which can then react with the nitrite ion to generate the active nitryl iodide intermediate. While the exact mechanism has not been elucidated, it has been proposed that this reactive species adds to the alkyne substrate *via* the formation of a vinyl radical or cation intermediate. The latter then undergoes a subsequent reaction with nitryl iodide or KI, resulting in the β‐iodonitroalkene as the final product (for key examples, see Scheme 11c).

A similar mechanistic scenario can be observed when using other nitrating reagents, such as silver nitrate [68], guanidine nitrate [69], or even nitric acid [70], where the activator facilitates the in situ formation of the electrophilic or radical nitrating species, which then engages the unsaturated substrate in a comparable difunctionalization process.

### 3.3 Electrolysis

**SCHEME 12:**
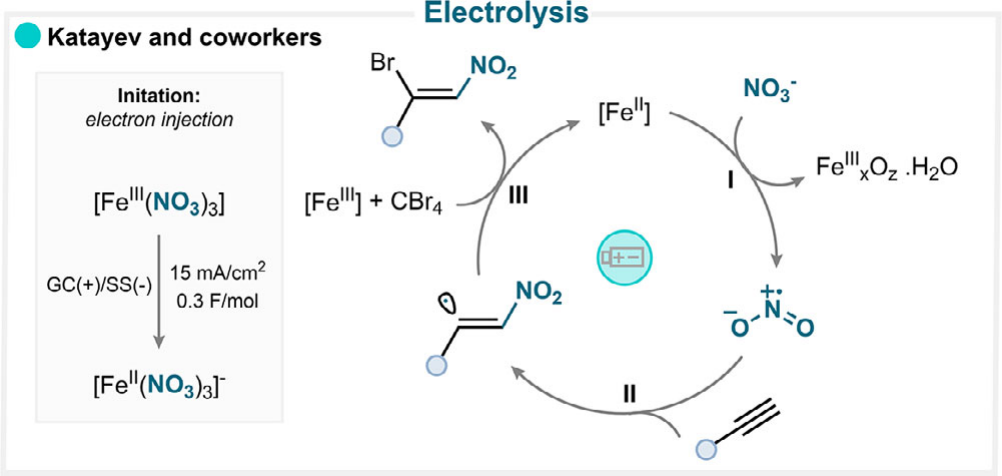
Key proposed mechanism of alkyne nitration via indirect electrolysis by Katayev and coworkers [71].

Our group has reported an electrochemical strategy for alkyne difunctionalization mediated by iron(III) nitrate (Scheme 12) [71]. The study demonstrated that this iron salt undergoes direct single‐electron reduction at the cathode to produce nitryl radicals at ambient temperature. The latter species then adds to the alkyne substrate to form a vinyl radical (**II**), which is trapped by CBr_4_, yielding a β‐bromonitroalkene product (**III**, and for key examples, see Scheme 11c) [71].

### 3.4 Oxidant‐Mediated Nitration

The use of an oxidant in combination with a nitrating reagent, such as sodium nitrite, efficiently generates the nitryl radical, which can then engage in radical addition to an alkyne.

**SCHEME 13:**
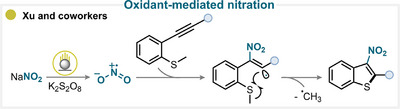
Key proposed mechanism of oxidant‐activated alkyne nitration by Xu and coworkers [72].

The Xu group explored the combination of sodium nitrite and potassium persulfate to produce •NO_2_ and to engage it in a selective addition reaction with alkyne (Scheme 13) [72]. This strategy involved the intermediacy of a high‐energy vinyl radical that underwent intramolecular cyclization with a methylthio substituent. This exergonic S_H_2 (radical homolytic substitution) event afforded the final product with the concomitant release of a methyl radical (for key examples, see Scheme 11c) [72].

A related transformation can also occur when the vinyl radical cyclizes intramolecularly onto an aromatic ring, leading to the formation of nitro‐substituted cyclic frameworks, further highlighting the versatility of this oxidative nitration strategy in C–C bond‐forming and ring‐closing reactions (for key examples, see Scheme 11c) [73].

### 3.5 Overview of the Alkyne Nitration

The assessment of the efficiency of various nitrating agents with respect to the key criteria is presented in Scheme 11. This analysis reveals that copper nitrate (**R15**) is the top performer in alkyne nitration with a score of 5.2, followed by TBN (**R13**) with a score of 4.7.

Alkyne nitration has not been widely studied and, as a result, it still faces significant limitations, particularly regarding *functional group tolerance* and *reagent activation* ‒ except when using iron nitrate. These limitations are common across most frequently used reagents, although the functional group tolerance is somewhat better for **R15**.

Among other top performers, iron nitrate (**R1**) with a score of 4.6 and guanidinium nitrate (**R17**) with a score of 4.0 show a relatively good performance.

## 4 Olefin Nitration

### 4.1 Thermal Activation

The general strategy relies on thermolabile reagents that, upon heating, generate a nitryl radical or nitronium ion, which subsequently engages the olefin to furnish the desired product. This approach has been demonstrated with a range of reagents, including ferric nitrate nonahydrate [76, 77, 78, 79, 80, 81, 82], copper nitrate trihydrate [74, 75, 83, 84], sodium nitrite [85], nitric acid [86], silver nitrite [87, 88], magnesium nitrate hexahydrate [89, 90], and TBN (through the formation of NO and subsequent oxidation to nitryl radical) [91, 92, 93, 94, 95, 96, 97, 98, 99, 100, 101, 102, 103, 104, 105, 106, 107].

**SCHEME 14:**
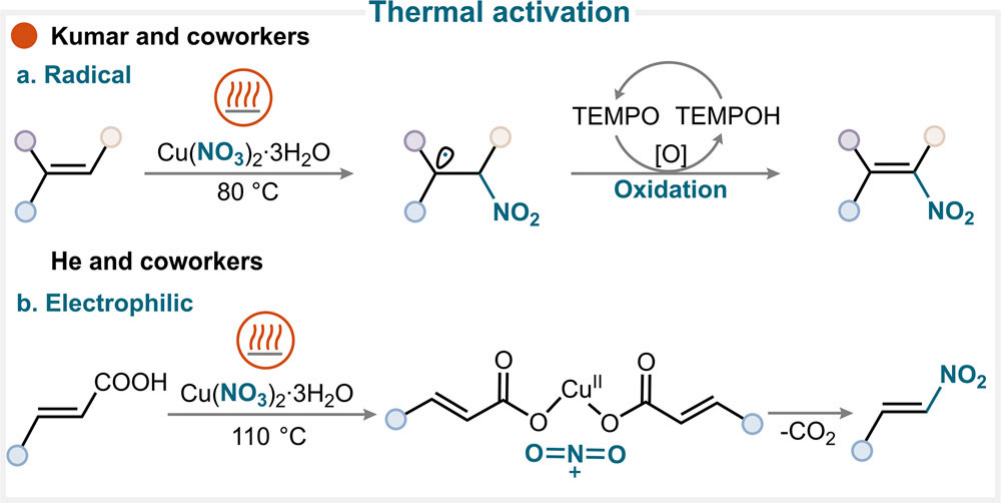
Key proposed mechanism for olefin nitration under thermal conditions by (a) Kumar and coworkers [74] and (b) He and coworkers [75].

Kumar and colleagues demonstrated that copper nitrate was a convenient source of nitryl radicals under thermal conditions. These radicals reacted with olefins, and the subsequent oxidation of the alkyl radical intermediate using TEMPO (2,6,6‐tetramethylpiperidin‐1‐yl)oxy) afforded the corresponding nitroalkene (Scheme 14a) [74]. In other cases, difunctionalization of alkene can be achieved through a radical ligand transfer (RLT) pathway: the nitryl radical generated from iron nitrate adds to olefin and produces an alkyl radical intermediate, which undergoes RLT with iron trichloride to afford the corresponding nitrohaloalkane (for key examples, see Scheme 20c) [78]. Finally, the alkyl radical intermediate may also undergo intramolecular cyclization, leading to the formation of cyclic nitroalkane (for key examples, see Scheme 20c) [89, 102]. An alternative approach exploited by Roy and coworkers relied on a nitrative decarboxylation protocol involving nitric acid and a catalytic amount of AIBN (azobis*iso*butyronitrile) [86].

In certain cases, the use of copper nitrate can give rise to multiple plausible pathways [75]. The reagent may directly release the nitryl radical, or alternatively, generate nitric acid in situ, which can subsequently release nitronium ion in solution (Scheme 14b) [75].

### 4.2 Photoredox Catalysis

**SCHEME 15:**
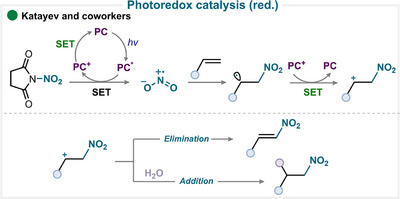
Key proposed mechanism for olefin nitration under photoredox catalysis by Katayev and coworkers [108].

In the context of photoredox catalysis, our group has published several studies employing *N*‐nitrosuccinimide as the nitrating reagent, enabled by SET reduction by a ruthenium‐ or iridium‐based photocatalyst [108, 109, 110]. Upon visible‐light irradiation (e.g., blue LEDs), the photocatalyst is excited and undergoes a SET with the nitrating reagent, triggering N–N bond mesolytic cleavage and generating the nitryl radical (Scheme 15). This radical adds to the olefin, producing an alkyl radical intermediate, which is typically oxidized by the photocatalyst to form a carbocation, thereby enabling catalytic turnover. The generated carbocation can then participate in cyclization or difunctionalization reaction, or a base may be used to afford alkene adduct (for key examples, see Scheme 20c). Building on this foundation, subsequent work has applied similar conditions to other substrates [111]. In the presence of a ketone, this pathway can also deliver isoxazolines (for key examples, see Scheme 20c) [108]. Alternatively, the alkyl radical intermediate can engage in RLT with a metal complex, providing nitro‐haloalkane product [34, 110].

### 4.3 In Situ Activation

In situ activation strategies generally rely on an activating moiety such as trifluoroacetic anhydride, triflic anhydride, trimethylsilyl chloride, Pd‐species, acetic acid, or acetyl chloride, in combination with various nitrate salts such as tetrabutylammonium nitrate [112, 113], copper nitrate trihydrate [84, 114, 115], silver nitrate [116, 117], guanidine nitrate [69], or cerium ammonium nitrate [118]. These systems enable the generation of highly reactive nitrating intermediates under mild conditions.

**SCHEME 16:**
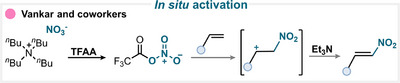
Proposed mechanism of in situ activated olefin nitration by Vankar and coworkers [112].

In the case of anhydrides (e.g. Ac_2_O, Tf_2_O, TFAA), the general strategy involves activation of the nitrate anion to generate a nitronium acetate (or triflate) in situ (Scheme 16) [112, 113, 117, 118]. This powerful nitrating agent can react with olefins via an ionic pathway to form a carbocation intermediate, which, after deprotonation, delivers the desired nitroalkene (for key examples, see Scheme 20c). Acetyl chloride‐mediated activation parallels the anhydride pathway, generating nitronium acetate species in situ [112, 116]. For trimethylsilyl chloride‐based systems, reaction with nitrate salts affords in situ silyl nitrate, which can be further converted into nitryl chloride in the presence of excess TMSCl [69, 115]. Nitryl chloride acts as the active nitrating species and can react with alkenes to furnish the corresponding nitrated products. A special case arises in Pd‐catalyzed nitro‐hydroxylation reaction, where the slow in situ generation of nitronium ion by Pd(II) from nitric acid is believed to minimize side reactions such as aromatic nitration. In this scenario, the Pd–π‐alkene complex undergoes electrophilic nitration by the external nitronium ion [119]. Subsequent reductive elimination furnishes the nitrohydroxylated alkane, albeit with limited diastereoselectivity.

### 4.4 Light‐Mediated Nitration

**SCHEME 17:**
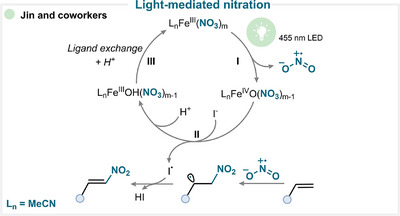
Key proposed mechanism of light‐mediated olefin nitration by Jin and coworkers [120].

Jin and coworkers demonstrated that ferric nitrate nonahydrate can serve as an efficient nitrating reagent under visible‐light irradiation (Scheme 17) [120]. In their study, the excited Fe(III) complex undergoes O–NO_2_ bond homolysis, releasing a nitryl radical along with a Fe(IV) = O complex (**I**). The electrophilic nitryl radical undergoes addition to the olefin, generating a benzylic radical intermediate. This radical is subsequently trapped by an iodine radical produced via SET from the Fe(IV) species (**II**). The resulting β‐iodonitroalkane undergoes spontaneous elimination of HI to afford the desired *E*‐nitroalkene (For key examples, see Scheme 20c) [120].

### 4.5 Metal‐Mediated Radical Substitution

**SCHEME 18:**
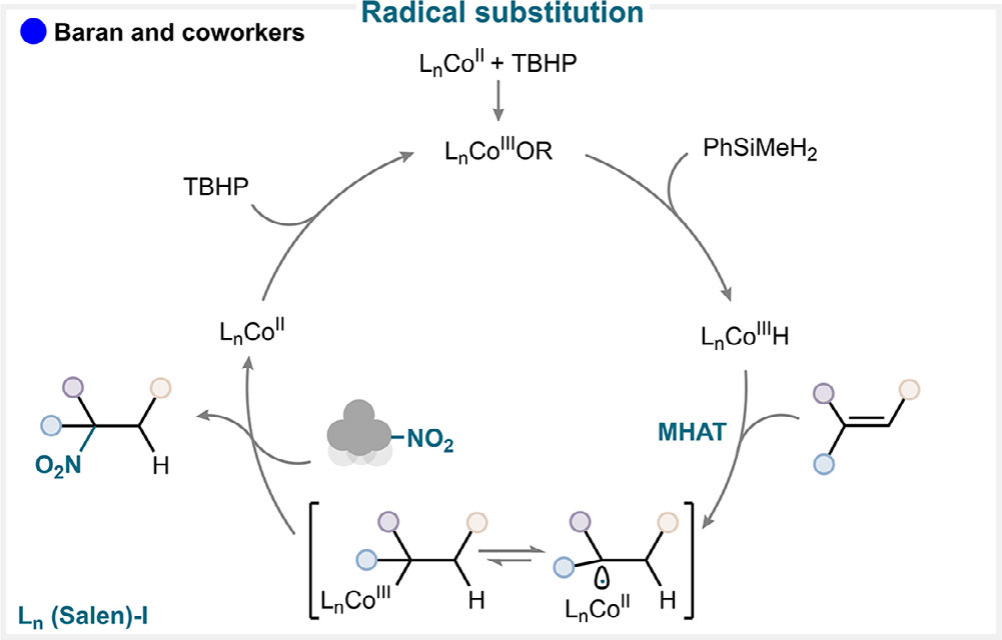
Key proposed mechanism of olefin nitration by Baran and coworkers [121].

Baran and coworkers reported a hydronitration of alkenes using a cobalt salen complex and a newly designed anomeric nitroamide reagent [121]. In this scenario, Co(II) is initially oxidized by *tert*‐butylhydroperoxide and subsequently reacts with phenylsilane to generate a Co(III) hydride. A subsequent metal hydride atom transfer (MHAT) step generates a tertiary radical (Scheme 18). Although the exact mechanism remains unclear, a radical‐polar crossover pathway was found unlikely. Instead, it is proposed that the nitroamide reagent delivers the NO_2_ group to the radical through participation of a Co(III) intermediate (for key examples, see Scheme 20c).

### 4.6 Electrolysis

**SCHEME 19:**
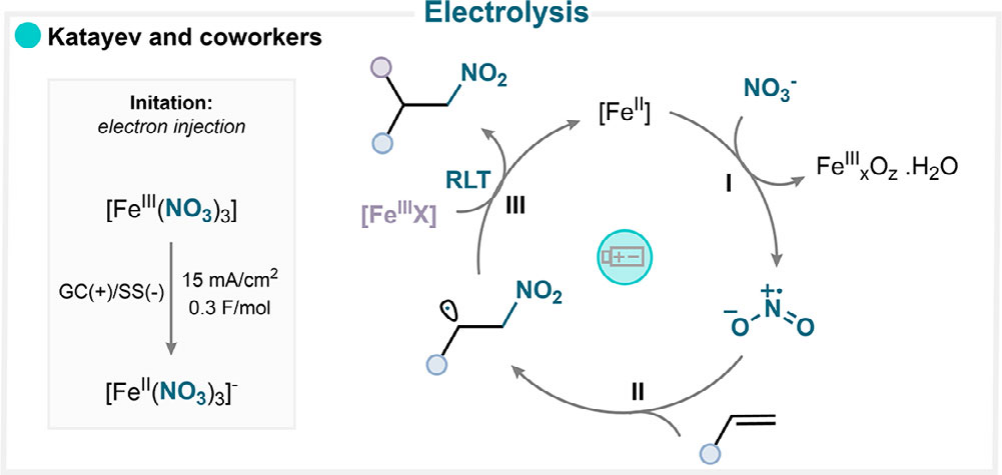
Key proposed mechanism of olefin nitration via indirect electrolysis by Katayev and coworkers [71].

**SCHEME 20:**
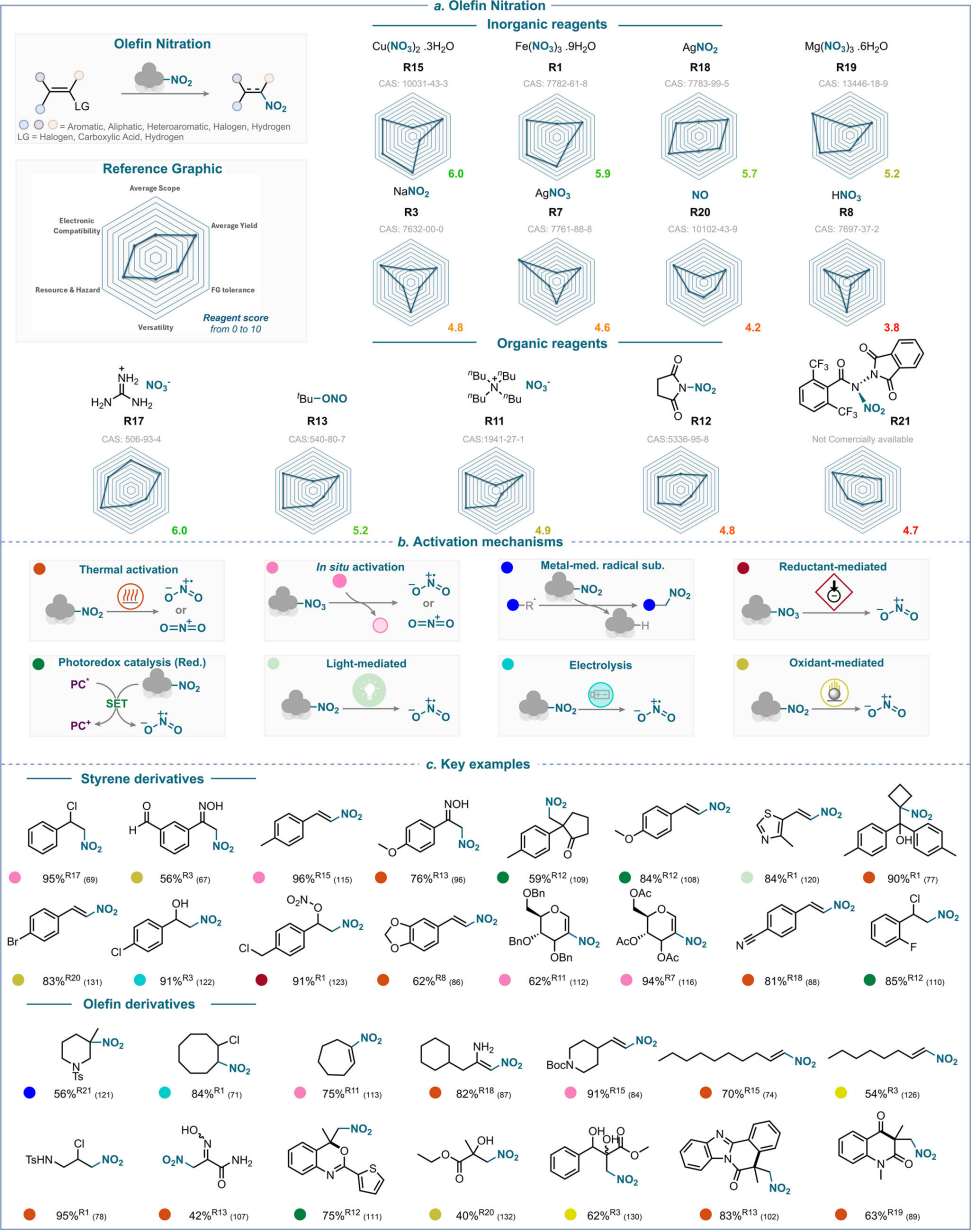
Overview of olefin nitration strategies over the past 25 years. (a) Nitrating reagents used, ranked from best to least performing, and divided between organic and inorganic natures. (b) Activation mechanisms encountered. (c) Selected examples using styrene and olefin derivatives. The colored balls indicate the activation method used, and the superscript number after the yield refers to the nitrating reagent used. The reference is given in brackets.

Our group reported an electrochemical strategy for the difunctionalization of olefins using iron nitrate [71]. Mechanistic studies demonstrated that only a catalytic amount of electrons was necessary in this reaction to reduce Fe(III) to Fe(II) at the cathode through direct single‐electron reduction. This generates a nitryl radical (Scheme 19), which adds to the alkene (**II**), and the resulting alkyl radical subsequently undergoes an RLT between the radical and the iron intermediate (**III**), affording the difunctionalized product (for key examples, see Scheme 20c) [71].

Building on this concept, Zhang and coworkers expanded the electrochemical nitration approach by employing sodium nitrite as the nitro source [122]. In their proposed mechanism, a nitrite anion is oxidized at the anode to produce a nitryl radical, which adds to a styrenic olefin. The resulting benzyl radical intermediate is subsequently oxidized by Cu(II) to generate a benzylic carbocation while reducing the copper catalyst to Cu(I). This carbocation then reacts with a nucleophile present in the reaction medium, leading to the desired functionalized product (for key examples, see Scheme 20c) [122].

### 4.7 Reductant‐Mediated Nitration

**SCHEME 21:**
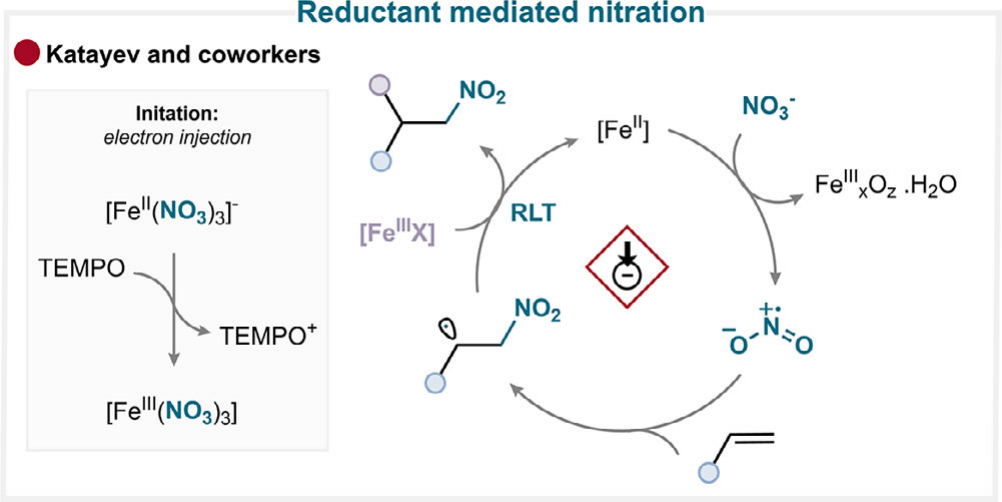
Key proposed mechanism of olefin nitration by Katayev and coworkers [123].

Building upon our work on electrochemical nitration [71], we further extended the electron catalysis strategy to mechanochemical conditions (Scheme 21) [123]. It was hypothesized that catalytic amounts of electrons delivered by TEMPO could reduce ferric nitrate to generate the nitryl radical. The study suggests that the nitrate anion (NO^3–^] can be reduced to NO_3_•^2–^, which, upon protonation, releases the nitryl radical. This radical adds to styrenic olefin affording a nitroalkyl intermediate, and subsequent RLT with ferric nitrate introduces a nitrate group, ultimately furnishing the difunctionalized product (for key examples, see Scheme 20c) [123].

### 4.8 Oxidant‐Mediated Nitration

A common strategy for nitryl radical generation relies on the oxidation of a nitrate or nitrite (copper nitrate trihydrate [83], sodium nitrite [67, 124, 125, 126, 127, 128, 129, 130], or nitric oxide [131, 132] precursor by an external oxidant. Various oxidants have been successfully employed, including K_2_S_2_O_8_ [124, 125, 126, 127], oxone [67, 83, 129], I_2_O_5_ [128], and even molecular oxygen [131, 132], all of which promote the homolytic cleavage required to release the electrophilic nitryl radical.

**SCHEME 22:**
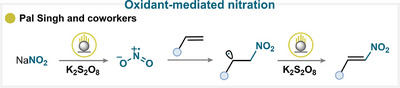
Key proposed mechanism of olefin nitration by Pal Singh and coworkers [124].

Once generated, this radical typically undergoes addition to an olefin, producing a carbon‐centered radical intermediate (Scheme 22). From this point, several divergent reaction pathways are possible depending on the system and reaction conditions. Following a radical‐polar crossover (RPC) pathway, the radical can be oxidized, followed by deprotonation to restore a C = C bond and yield the corresponding β‐nitroolefin (for key examples, see Scheme 20c) [67, 124, 126, 128, 131]. Alternatively, the intermediate may participate in intramolecular trapping, enabling the synthesis of cyclized nitroalkane [83, 125, 127]. Under certain conditions, the radical can react further with nitric oxide to afford α‐nitrooximes, providing a direct route to valuable bifunctionalized products [129]. Reaction with molecular oxygen is another productive pathway, giving rise to oxygenated species such as nitro alcohols, nitro ketones, or nitroso nitro derivatives (for key examples, see Scheme 20c) [132]. These oxidative activation approaches provide a versatile platform in which the choice of oxidant and reaction environment determines whether simple nitration, cyclization, or difunctionalization predominates.

### 4.9 Overview of the Olefin Nitration

An assessment of the reagents’ efficiency with respect to the key criteria is provided in Scheme 20. This analysis reveals that copper nitrate (**R15**) and guanidinium nitrate (**R17**) are the top performers in olefin nitration with a score of 6.0. Although both reagents have the same score, **R17** is more balanced across all six criteria, while **R15** exhibits weaknesses in terms of *average scope* and *functional group tolerance*. In contrast, key strengths of **R15** over **R17** lie in the *scope diversity* and reagent *versatility*.

Among other top performers, iron nitrate (**R1**) with a score of 5.9 exhibits limited *FG tolerance* similar to **R15** and **R17**. Nevertheless, improved *FG tolerance* is noted for silver nitrite (**R18**) which possesses a score of 5.7.

## 5 Heteroatom Nitration

### 5.1 In Situ Activation

**SCHEME 23:**
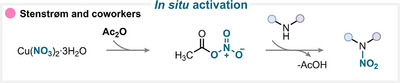
Key proposed mechanism of heteroatom nitration by Stenstrøm and coworkers [133].

Nitric acid or nitrate salts can be efficiently activated in combination with various anhydrides, such as acetic or trifluoroacetic anhydride, to afford the desired nitrated products (for key examples, see Scheme 25c) [133, 134, 135, 136, 137, 138, 139, 140, 141, 142, 143]. A representative example of this activation mode was reported by Stenstrøm and coworkers, who demonstrated that copper nitrate in acetic anhydride generates in situ a reactive nitrating intermediate (Scheme 23) [133]. This electrophilic species can subsequently react with amine substrate, leading to nitroamine products under mild conditions (for key examples, see Scheme 25c) [133, 143]. Similarly, silver nitrate in combination with triphenylphosphine can produce a comparable reactive nitronium‐type intermediate, which serves as an efficient nitrating agent to access a variety of nitrated organic compounds (for key examples, see Scheme 25c) [144].

### 5.2 Acid‐Assisted Nitration

**SCHEME 24:**
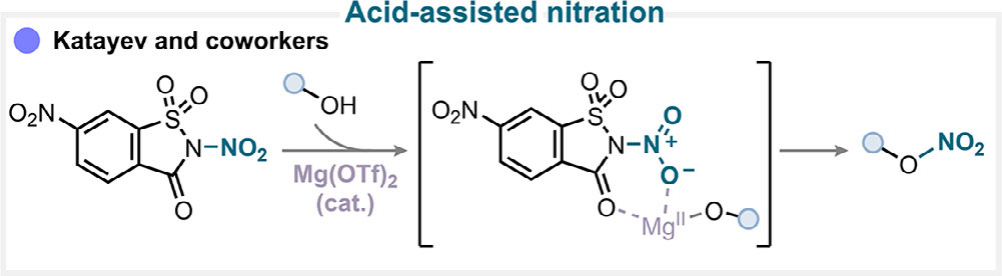
Key proposed mechanism of acid‐assisted heteroatom nitration by Katayev and coworkers [145].

**SCHEME 25:**
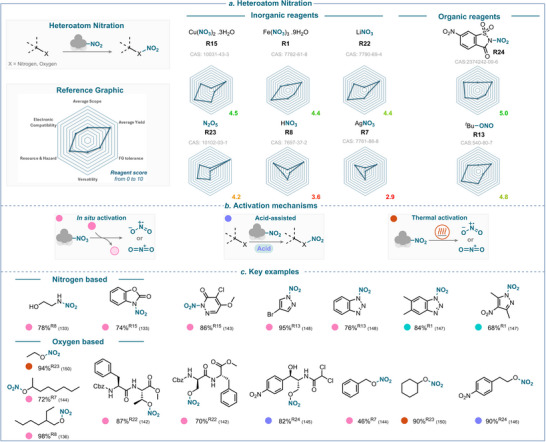
Overview of heteroatom nitration strategies over the past 25 years. (a) Nitrating reagents used, ranked from best to least performing, and divided between organic and inorganic natures. (b) Activation mechanisms encountered. (c) Selected examples using nitrogen‐ and oxygen‐based derivatives. The colored balls indicate the activation method used, and the superscript number after the yield refers to the nitrating reagent used. The reference is given in brackets.

In a study conducted by our group on the nitration of alcohols, we provided detailed ab initio mechanistic insights into the activation pathway of the *N*,6‐dinitrosaccharin reagent [145]. The mechanism involves coordination of the magnesium triflate Lewis acid to both the carbonyl moiety and the NO_2_ group of the reagent (Scheme 24). Subsequent N–N bond cleavage and nitronium ion release is followed by nucleophilic attack from the alcohol substrate, leading to the formation of the corresponding nitrate esters (for key examples, see Scheme 25c) [145]. Importantly, this Lewis acid‐promoted nitration could be carried out efficiently not only under conventional solution‐phase conditions but also under mechanochemical conditions [146]. This finding highlights the robustness and adaptability of the methodology across different reaction environments.

### 5.3 Thermal Activation

**SCHEME 26:**
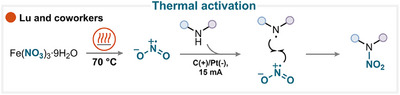
Key proposed mechanism of thermally activated heteroatom nitration by Lu and coworkers [147].

An elegant combination of thermal activation and electrocatalysis was proposed by Lu and coworkers, who employed ferric nitrate as the nitrating reagent (Scheme 26) [147]. In their study, heating the mixture to 70°C facilitated the generation of the nitryl radical. Simultaneously, the *N*‐heterocyclic substrate was anodically oxidized to produce a nitrogen‐centered radical. A subsequent radical–radical coupling then efficiently delivered the desired nitroamine products. This dual‐activation strategy demonstrates that thermal and electrochemical pathways can synergistically enable nitration under relatively mild conditions, without the need for a chemical oxidant (for key examples, see Scheme 25c) [147]. Analogously, Zhuang and coworkers obtained results similar to those using TBN as a nitrating reagent and cerium ammonium nitrate (CAN) as an oxidant [148, 149].

Other thermally activated nitration pathways have been reported, for instance, using dinitrogen pentoxide as the nitrating reagent. Upon heating, N_2_O_5_ releases nitronium ion, which can subsequently engage in the *O*‐nitration reaction to afford the corresponding nitrate esters (for key examples, see Scheme 25c) [150].

### 5.4 Overview of the Heteroatom Nitration

An assessment of the reagents' efficiency with respect to the key criteria is provided in Scheme 25. This analysis reveals that *N*,6‐dinitrosaccharin (**R24**) performs well in heteroatom nitration, with a score of 5.0. While **R24** is relatively well‐balanced, it still lacks *versatility* in activation mode, as it is currently restricted to Lewis acid activation.

Among the other top performers, TBN (**R13**) with a score of 4.8, exhibits lower *FG tolerance* than **R24**, yet it can be activated under various conditions, including in the presence of acids or upon heating. Iron nitrate also performed relatively well in heteroatom nitration with a global score of 4.4.

## 6 Aromatic Nitration

### 6.1 Mechanical Activation

**SCHEME 27:**
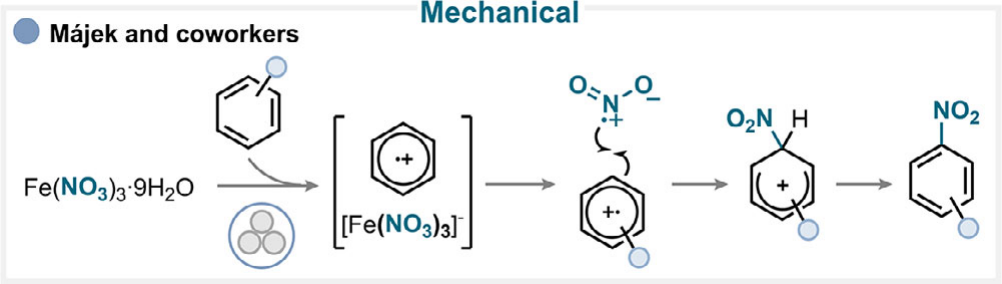
Key proposed mechanism for the aromatic nitration under mechanochemical conditions by Májek and coworkers [151].

Majek and coworkers harnessed the growing potential of mechanochemistry, specifically ball milling, to achieve aromatic nitration via mechanical activation [151]. In their study, iron(III) nitrate was proposed to form a charge‐transfer complex with the arene substrate under mechanical force (Scheme 27). This highly unstable complex readily decomposes to generate the nitryl radical, which subsequently reacts with the arene radical cation. The final deprotonation step affords the desired nitroaromatic product (for key examples, see Scheme 35c) [151].

On the other hand, Guo and coworkers employed the same nitrating reagent and phosphorus pentoxide (P_2_O_5_) as an additive [152]. The authors suggested that under mechanical stress, P_2_O_5_ promotes the formation of the nitryl radical from iron nitrate, leading to efficient aromatic nitration through a similar mechanistic pathway (for key examples, see Scheme 35c) [152].

### 6.2 Photoredox Catalysis

**SCHEME 28:**
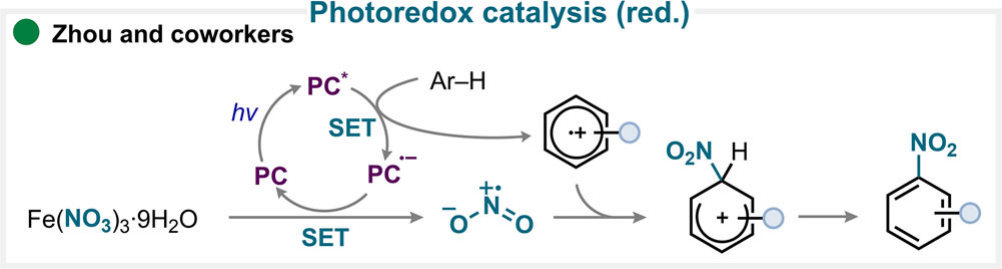
Key proposed mechanism for the photoredox‐catalyzed aromatic nitration by Zhou and coworkers [153].

Zhou and coworkers developed a photochemical aromatic nitration protocol using iron(III) nitrate in combination with riboflavin tetraacetate (RFTA) as the photosensitizer [153]. In the proposed mechanism, the photoexcited RFTA oxidizes the aromatic substrate to generate the corresponding radical cation (Scheme 28). Concurrently, the reduced form of the photocatalyst transfers an electron to ferric nitrate, triggering the release of a nitryl radical. The coupling of these two radical species, followed by deprotonation, delivers the desired nitroarene (for key examples, see Scheme 35c). This elegant dual‐activation strategy highlights the potential of organic photocatalysts to mediate both oxidation and reduction events within a single catalytic cycle [153]. Sodium nitrite has also been employed under analogous photoredox conditions for the nitration of anilines [154].

### 6.3 In Situ Activation

The in situ activation concept is among the most widely employed strategies for aromatic nitration and has been demonstrated using a broad range of nitrating reagents, including iron nitrate [156, 157, 158], TBAN [155, 159], copper nitrate [160, 161, 162], *tert*‐butyl nitrite [163], sodium nitrite [164], sodium nitrate [165, 166], silver nitrate [167, 168], silver nitrite [169], urea nitrate [170], guanidine nitrate [171], aluminum nitrate [172, 173], lithium nitrate [172], [Bi_5_O(OH)_9_(NO_3_)_4_] [174], ceric ammonium nitrate [118], ethylammonium nitrate [175], and nitromethane [176]. In most cases, these nitrating agents are not sufficiently reactive to directly engage in electrophilic aromatic substitution. Therefore, additives are employed to generate a more reactive nitrating intermediate in situ. Such additives can include anhydrides, TMSCl, thionyl chloride (SOCl_2_), ZnCl_2_, CuCl_2_, hypervalent iodine reagents, bases, cobalt or palladium complexes, or water, depending on the system.

**SCHEME 29:**
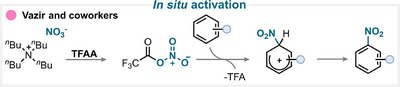
Key proposed mechanism for the aromatic nitration using an in situ‐generated reagent by Vazir and coworkers [155].

A representative example of this strategy was reported by Vazir and coworkers (Scheme 29), who combined TBAN with trifluoroacetic anhydride (TFAA) to generate a reactive nitrating species capable of undergoing electrophilic aromatic substitution and affording the desired nitroarene after subsequent deprotonation (for key examples, see Scheme 35c) [155].

### 6.4 Light‐Mediated Nitration

**SCHEME 30:**
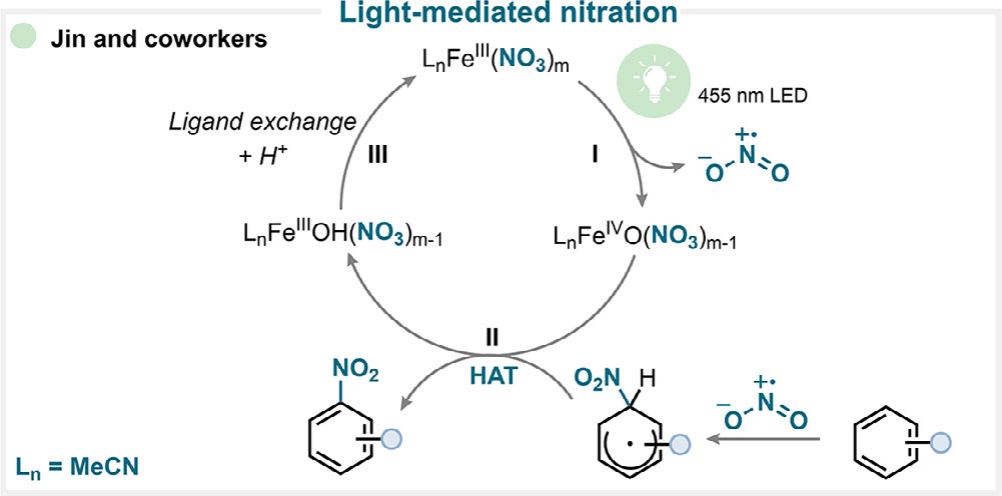
Key proposed mechanism for the aromatic nitration by reagent photolysis by Jin and coworkers [177].

In an interesting study by Jin and colleagues, the nitryl radical is formed via direct photolysis of iron nitrate under 395 nm irradiation (Scheme 30, **I**) [177]. The generated nitryl radical adds to the aromatic ring, yielding the corresponding cyclohexadienyl radical intermediate. The iron(IV) species is then proposed to facilitate a HAT (**II**), leading to the formation of the desired nitroarene (for key examples, see Scheme 35c).

A similar mechanism was envisioned for 5‐methyl‐dinitroimidazole (DNIm), in which irradiation at 395 nm promoted homolytic cleavage of the N–NO_2_ bond to generate a nitryl radical, which was then engaged with various aromatic substrates to afford the corresponding nitroarenes (for key examples, see Scheme 35c) [178, 179].

### 6.5 Acid‐Assisted Nitration

Acid‐assisted aromatic nitration is a well‐established strategy that was traditionally carried out under “mixed acid” conditions, where the high acidity of sulfuric acid was used to generate nitronium ions from nitric acid. Owing to the rather harsh conditions and the strong reactivity of this system, this canonical approach has several limitations that can be mitigated by using alternative nitrating sources and acids.

**SCHEME 31:**
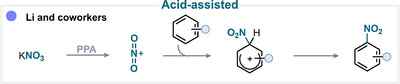
Key proposed mechanism for the acid‐assisted aromatic nitration by Li and coworkers [180].

For example, in a very recent contribution by the Li group (Scheme 31), potassium nitrate was employed with polyphosphoric acid (PPA) to generate nitronium ion, which subsequently undergoes S_E_Ar to afford the desired nitroaromatic compounds (for key examples, see Scheme 35c) [180].

This general strategy is applicable to a wide range of nitrating reagents, including iron nitrate [37, 181], tetrabutylammonium nitrate [182], *N*‐nitrosaccharin [183], *N*,6‐dinitrosaccharin [146], sodium nitrite [184], sodium nitrate [185, 186, 187], potassium nitrate [180, 188], nitric acid [189, 190, 191, 192, 193, 194, 195, 196, 197, 198, 199, 200, 201, 202, 203], urea nitrate [204], guanidine nitrate [205], lithium nitrate [206], bismuth nitrate [207, 208], nickel nitrate [209], 3‐methyl‐1‐sulfonic acid imidazolium nitrate [210], thiourea nitrate [211], yttrium nitrate [212], 5‐methyl‐dinitroimidazole [148], and dinitro‐5,5‐dimethylhydantoin [213].

While most of these systems follow similar mechanistic principles, several notable efforts have been made to develop greener, more sustainable variants, for instance, through mechanochemical approaches that enable acid‐assisted nitration under solvent‐free conditions (for key examples, see Scheme 35c) [146, 208].

### 6.6 Electrolysis

**SCHEME 32:**
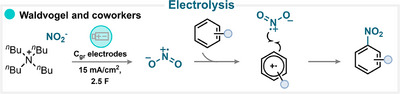
Key proposed mechanism for the aromatic nitration under electrolysis by Waldvogel and coworkers [214].

Waldvogel and coworkers reported an advantageous aromatic nitration protocol that employs nitrite salt and electrochemistry. Mechanistic studies revealed that the nitrite anion is anodically oxidized to generate the nitryl radical (Scheme 32) [214]. It is proposed that this nitryl radical dimerize in part into N_2_O_4_, which forms a charge‐transfer complex with the aromatic substrates upon ionic dissociation to [NO^+^] [NO_3_^−^]. This interaction facilitates the generation of the corresponding arene radical cations, which eventually couple with nitryl radicals and furnish the desired nitroaromatic product after the deprotonation (for key examples, see Scheme 35c). The possibility for the arene radical cation to undergo nucleophilic attack by nitrite anion could, however, not be excluded. This study provided valuable electrochemical insight into the direct anodic generation of reactive nitrating species [214].

### 6.7 Thermal Activation

**SCHEME 33:**
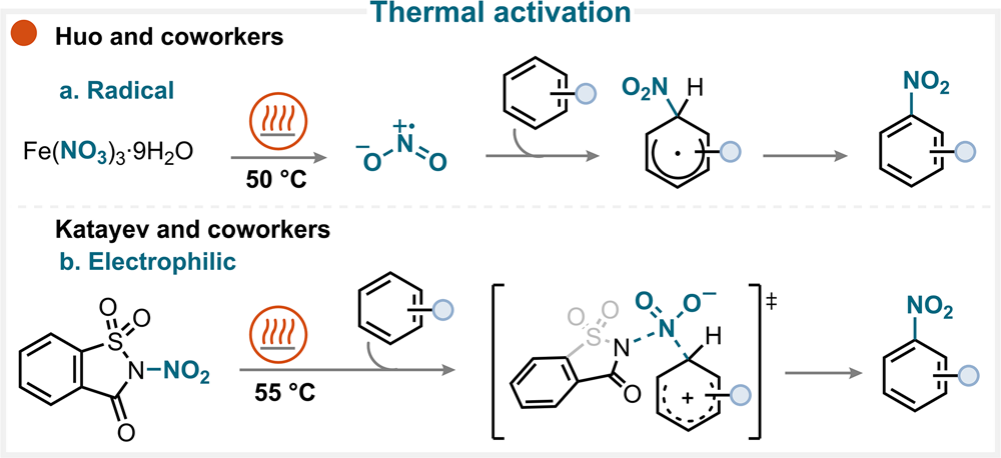
Key proposed mechanism for the thermal aromatic nitration by (a) Huo and coworkers [215] and (b) Katayev and coworkers [183].

Thermal conditions can give rise to two distinct active nitrating species: the nitryl radical and the nitronium ion. In the work of Huo and coworkers, it was demonstrated that heating iron(III) nitrate to as little as 50°C is sufficient to generate the nitryl radical (Scheme 33a). This reactive species can add to the aromatic ring, and a subsequent rearomatization event yields the desired nitroaromatic product (for key examples, see Scheme 35c) [215].

An alternative electrophilic pathway involves the use of *N*‐nitrosaccharin under similar thermal conditions in HFIP (Scheme 33b). In this case, the reaction proceeds via thermal cleavage of the N–NO_2_ bond, concomitant with the delivery of the nitronium ion via a concerted yet highly asynchronous mechanism (for key examples, see Scheme 35c) [183].

A variety of nitrating reagents have been evaluated under thermal conditions, ranging from conventional species, including iron nitrate [215, 216, 217, 218, 219], copper nitrate [218], *tert*‐butyl nitrite [163, 220, 221, 222, 223, 224, 225, 226, 227, 228, 229, 230, 231, 232, 233, 234, 235, 236, 237, 238, 239], sodium nitrite [240], CAN [218, 241, 242], pyridinium nitrate [243], and methoxymethyl nitrate [244], to more sophisticated pyridinium salt‐based nitrate esters [245] and benziodazole‐type O_2_NO–I(III) reagents [246].

### 6.8 Oxidant‐Mediated Nitration

**SCHEME 34:**
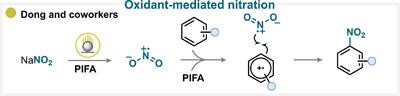
Key proposed mechanism for the aromatic nitration employing chemical oxidants by Dong and coworkers [247].

**SCHEME 35:**
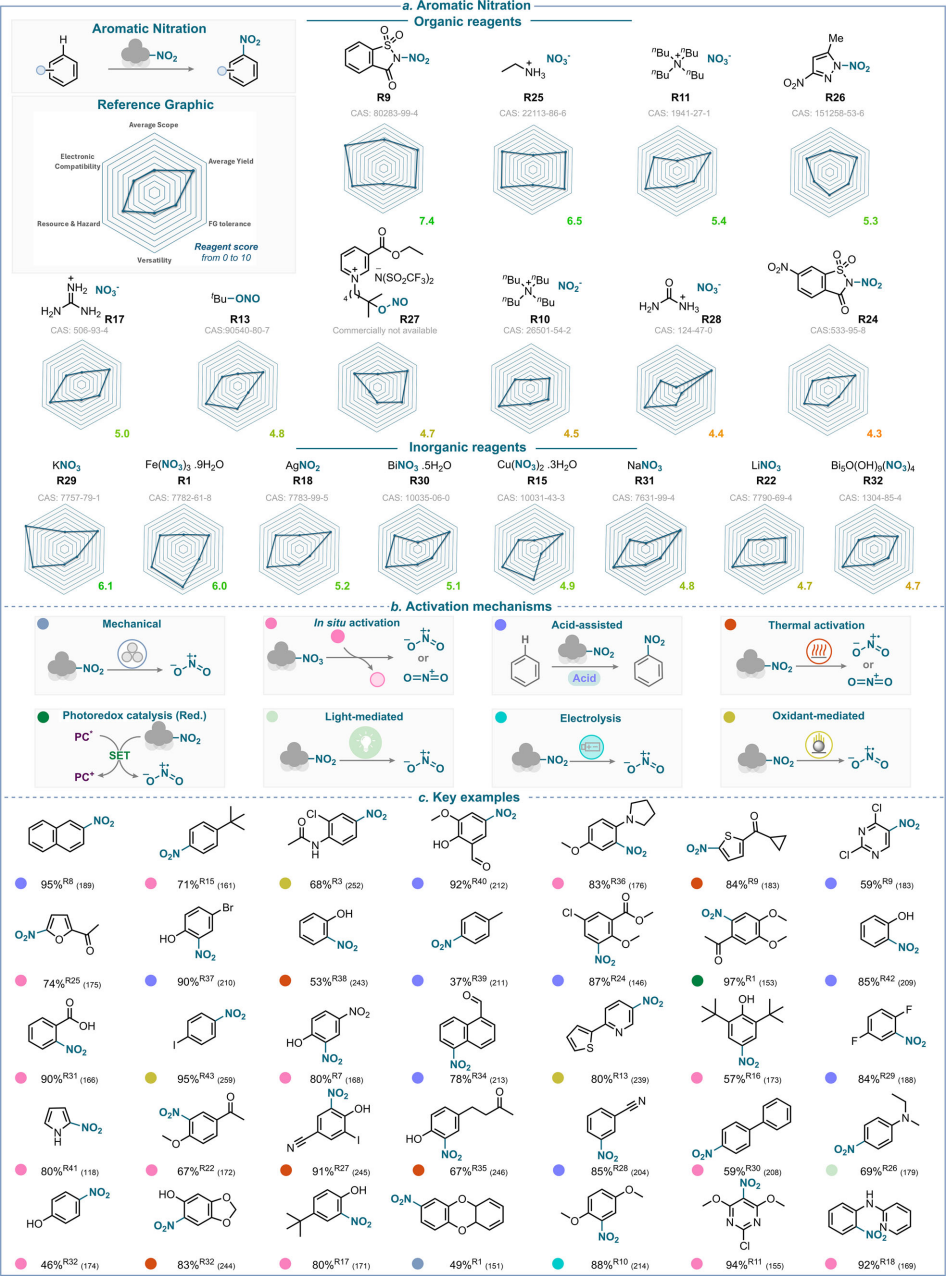
Overview of aromatic nitration strategies over the past 25 years. (a) Nitrating reagents used (see Supporting Information, page 6, for analysis of additional reagents), ranked from best to least performing, and divided between organic and inorganic natures. (b) Activation mechanisms encountered. (c) Selected examples. The colored balls indicate the activation method used, and the superscript number after the yield refers to the nitrating reagent used. The reference is given in brackets.

The formation of the nitryl radical through chemical oxidation of the nitrating reagent is among the most common strategies in aromatic nitration. A representative and powerful example is the system relying on sodium nitrite in combination with PIFA (Scheme 34). Under these conditions, PIFA oxidizes the nitrite anion to generate the nitryl radical while simultaneously oxidizing the aromatic substrate to its radical cation. The coupling of these two radical species affords the desired nitroarene (for key examples, see Scheme 35c) [247].

Comparable mechanistic pathways have been observed with other combinations of nitrating reagents and oxidants, such as copper nitrate [248, 249], *tert*‐butyl nitrate [250, 251], sodium nitrate [252], sodium nitrite [247, 252, 253, 254], silver nitrate [255, 256, 257], nitric acid [258], N_2_O_4_, [259], and silver nitrite [260], all operating through generation of the key nitryl radical through oxidation.

### 6.9 Overview of the Aromatic Nitration

An assessment of the reagents’ efficiency with respect to the key criteria is provided in Scheme 35. This analysis reveals that *N*‐nitrosaccharin (**R9**) is the top performer in aromatic nitration with a score of 7.4. A key limitation of **R9** is its lack of *versatility* with regard to activation mode, as it remains currently restrained to Lewis acid and thermal activations, yet it demonstrates excellent performance under such conditions.

Among other top performers, ethyl ammonium nitrate (**R25**), potassium nitrate (**R29**), and iron nitrate (**R1**) all exhibit scores above 6. As identified in other nitration processes, iron nitrate stands out with its superior *versatility*, being capable of generating both nitronium ion and nitryl radical under a plethora of reaction conditions.

Aromatic nitration is, without doubt, the most widely explored nitration reaction. This has led to the iterative improvement of reagents over the years, leaving the community with highly efficient and selective nitration processes.

## 7 Guide to Nitration

**SCHEME 36:**
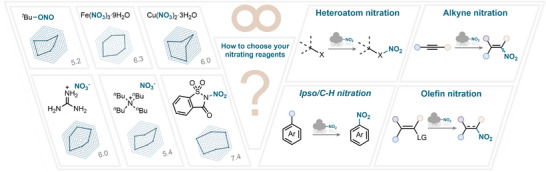
Representation of the best performing nitrating reagents (four organic and four inorganic) in the studied nitration reactions.

**SCHEME 37:**
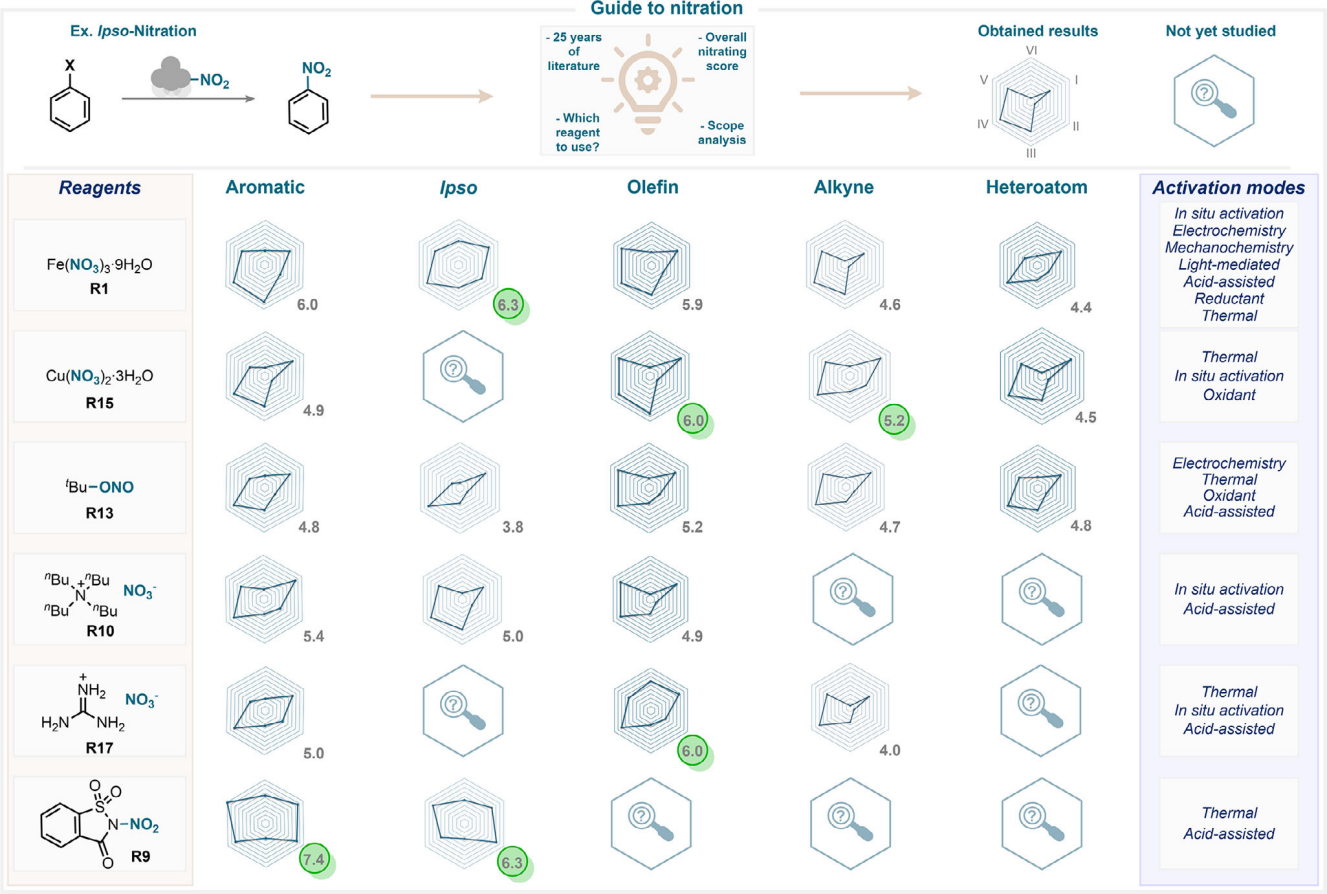
A guide to nitration chemistry showcasing the best‐performing nitrating reagents and their performance in the studied nitration reactions. (a) average scope, (b) average yield, (c) functional group tolerance, (d) versatility, (e) resource and hazard, (f) electronic compatibility.

Nitration chemistry can sometimes feel overwhelming, as a wide variety of approaches can be used to access the desired nitro products. The traditional mixed‐acid protocol, although demonstrating a powerful nitrating ability, often suffers from drawbacks. In this section of the review, we aim to provide guidance on selecting the most appropriate nitrating reagent for a given transformation (Scheme 36). To this end, we systematically compared the best‐performing reagents across the different reaction classes considered in this work, thereby offering a practical overview of which reagents are most suitable for specific applications (Scheme 37).

From our analysis, several NO_2_ sources consistently stood out as top performers in at least one transformation: iron nitrate nonahydrate, copper nitrate trihydrate, *tert*‐butyl nitrite, guanidinium nitrate, *tetra*‐butylammonium nitrate, and nitrosaccharin (Scheme 36). These compounds represent a diverse set of inorganic salts and organic‐based reagents, each with distinct activation requirements and mechanistic profiles. Among them, iron nitrate and TBN were by far the most frequently used in the literature, as reflected in Scheme 37, where both appear across all of the analyzed transformations. This frequent application is a valuable advantage: the wealth of conditions and precedents makes these two reagents particularly versatile starting points for synthetic planning.

Iron nitrate performed exceptionally well in *ipso*‐nitration and showed strong, though slightly less optimal, results in heteroatom nitration. Its versatility is remarkable, as it can participate in multiple activation modes, including thermal, photolytic, electrochemical, and mechanochemical pathways, while generating either the nitronium ion or the nitryl radical. The main drawback of iron nitrate is its non‐recyclable nature, which could limit its appeal for large‐scale applications where recyclable reagents and minimized waste are increasingly desirable [261]. Yet, the high natural abundance of iron on Earth still makes it a first‐choice reagent in the context of nitration chemistry.

Copper nitrate ranked among the most effective reagents overall, excelling in both olefin and alkyne nitration, while also exhibiting good results in heteroatom and aromatic systems. The only considered transformation for which it has not been applied is *ipso*‐nitration. Given its broad performance and availability, it is a useful reagent similar to iron nitrate.

*Tert*‐butyl nitrite was also broadly used, showing its best performance in olefin and heteroatom nitration, but underperforming in *ipso*‐nitration. A key limitation lies in its activation: TBN is primarily thermally activated, and its decomposition releases nitric oxide, which then requires an additional oxidant to generate the reactive nitryl radical. This two‐step requirement can pose challenges when working with thermally or oxidant‐sensitive substrates.

Nitrate salts, including tetrabutylammonium and guanidinium, are among the top‐performing nitrating sources. Tetrabutylammonium nitrate exhibited good and balanced performance across aromatic, olefin, and *ipso*‐nitration reactions. It is especially attractive due to its operational simplicity, as it is typically activated in situ by an anhydride or other activating agent to generate a reactive nitronium intermediate. While not yet applied in alkyne or heteroatom nitration, its practicality and robustness make it a strong candidate for expansion into these areas. Guanidinium nitrate performed well in both aromatic and olefin nitration, with somewhat weaker performance in alkyne nitration. The reported results suggest that it is a promising, yet underutilized option. The improved solubility of tetrabutylammonium and guanidinium salts in organic solvents certainly facilitated their widespread adoption and use in various nitration processes, compared to other inorganic salts, such as potassium nitrate. Although the latter performed well in aromatic nitration (Scheme 35, score 6.1), it has not been well explored in other transformations.

Finally, *N*‐nitrosaccharin has emerged as a promising organic nitrating reagent. It consistently delivered some of the highest overall scores, most notably in aromatic nitration, where it outperformed the other nitrating reagents. It is readily available, recyclable, and can be activated thermally or with Lewis acid catalysts, features that align well with modern demands for greener, more sustainable reagents. Although not yet explored in olefin, alkyne, or heteroatom nitration, its demonstrated efficiency strongly suggests it will soon find applications in these areas.

Taken together, these findings showcase that while a broad spectrum of nitrating reagents is available, each has unique strengths, limitations, and mechanistic profiles. A particularly striking observation is that nitric acid, despite being the historical cornerstone of nitration chemistry, consistently underperformed across nearly all transformations analyzed in this review. This further highlights the progress made over the years in facilitating the nitration of organic molecules and improving key aspects of modern synthetic chemistry, such as selectivity, functional group tolerance, and the generality of the methods.

## 8 Conclusion and Outlook

Nitration chemistry, despite significant progress, still faces several important challenges and clear room for improvement. One promising direction for future development lies in the use of greener, more sustainable methodologies, such as mechanochemistry, photochemistry, or electrochemistry. These strategies not only reduce the reliance on harsh reagents and conditions but also open new activation pathways that could expand the scope of nitration.

Another persistent challenge is direct Csp^3^–H nitration. Despite rapid advances in Csp^3^–H functionalization chemistry leveraging HAT or metal‐catalysis concepts, direct nitrative Csp^3^–H functionalization remains elusive. Nevertheless, ongoing progress in C–H activation chemistry suggests that practical approaches for site‐selective Csp^3^–H nitration may soon emerge. Notably, recent developments in radical ligand‐transfer chemistry offer a promising avenue for achieving radical functionalization under mild and controlled conditions. Such a strategy could become a game‐changer, enabling selective nitration of radicals [262].

Finally, asymmetric nitration remains a particularly challenging problem. While some groups have explored approaches using specific substrate derivatives to induce asymmetry, a broadly applicable catalytic method has yet to be established [263].

Addressing these challenges would represent major milestones in nitration chemistry, providing both more sustainable and more selective access to nitrogen‐containing molecules.

By highlighting alternative reagents and their activation strategies, we hope to guide practitioners toward more efficient, selective, and sustainable approaches to nitration chemistry, away from the hazards and limitations of mixed‐acid protocols. We anticipate that this review will stimulate the exploration of new areas of nitration chemistry.

The data that support the analysis of this review are available in the Supporting Information of this article.

## Conflicts of Interest

The authors declare no conflicts of interest.

## Supporting information




**Supporting File 1**: anie71946‐sup‐0001‐SuppMat.pdf.

## Data Availability

The data that support the findings of this study are available from the corresponding author upon reasonable request.
