# Supplementary material for: Navigating Nitration Chemistry: A Practical Guide to Reagents, Mechanisms, and Selectivity
Source: Angew Chem Int Ed Engl. 2026 Mar 25;65(20):e26128. doi: 10.1002/anie.202526128 (PMC13159431; doi:10.1002/anie.202526128)

Navigating Nitration Chemistry: A Practical Guide to Reagents, Mechanisms, and Selectivity

Harry Lecomte, Anthony J. Fernandes, Dmitry Katayev*

Department für Chemie und Biochemie, Universität Bern, Freiestrasse 3, 3012 Bern (Switzerland)
KEYWORDS Nitration, Electrophilic reactions, Radical reactions, Reagents, Mechanisms.

I. PERFORMANCE SCORE **3**

1. *IPSO*-NITRATION 4
$NaNO_2$ (R3) 5
N-Nitrosuccinimide (R12) 9
KNO_2 (R2) 10
HNO_3 (R8) 11
$Fe(NO_3)_3.9H_2O$ (R1) 12
Tetra-butyl ammonium nitrite/nitrate (R10/R11) 14
N-Nitrosaccharin (R9) 16
$Bi(NO_3)_3.5H_2O$ (R5) 17
NO_2BF_4 (R6) 19
1,3-disulfonic acid imidazolium nitrate (R14) 20
$ZrO(NO_3)$. xH_2O (R4) 21
Tert-butyl nitrite (R13) 22
$AgNO_3$ (R7) 23
2. ALKYNE NITRATION 24
$Fe(NO_3)_3.9H_2O$ (R1) 25
Tert-butyl nitrite (R13) 26
$NaNO_2$ (R3) 30
$AgNO_3$ (R7) 32
$Al(NO_3)_3.9H_2O$ (R16) 33
$Cu(NO_3)_2.3H_2O$ (R15) 34
Guanidine nitrate (R17) 35
HNO_3 (R8) 36
3. OLEFIN NITRATION 37
$Fe(NO_3)_3.9H_2O$ (R1) 38
Tetra-butyl ammonium nitrate (R11) 43
N-Nitrosuccinimide (R12) 44
$Cu(NO_3)_2.3H_2O$ (R15) 46
Tert-butyl nitrate (R13) 49
$NaNO_2$ (R3) 56
NO (R20) 61
$AgNO_3$ (R7) 62
HNO_3 (R8) 63
$AgNO_2$ (R18) 64
$Mg(NO_3)_2.6H_2O$ (R19) 65
Anomeric nitroamide (R21) 66
Guanidine nitrate (R17) 67

4. HETEROATOM NITRATION 68
$Cu(NO_3)2.3H_2O$ (R15) 69
$Fe(NO_3)_3.9H_2O$ (R1) 70
$LiNO_3$ (R22) 71
N_2O_5 (R23) 72
HNO_3 (R8) 73
$AgNO_3$ (R7) 77
N,6-dinitrosaccharin (R24) 78
Tert-butyl nitrite (R13) 79
5. AROMATIC NITRATION 80
$Fe(NO_3)_3.9H_2O$ (R1) 81
Tetra-butyl ammonium nitrate (R11) 87
Tetra-butyl ammonium nitrite (R10) 89
6,N-dinitrosaccharin (R24) 90
N-nitrosaccharin (R9) 91
$Cu(NO_3)_2.3H_2O$ (R15) 92
Tert-butyl nitrite (R13) 95
$NaNO_2$ (R3) 105
$NaNO_3$ (R31) 110
KNO_3 (R29) 113
$AgNO_3$ (R7) 114
HNO_3 (R8) 117
N_2O_4 (R43) 124
$AgNO_2$ (R18) 125
Urea Nitrate (R28) 126
Guanidine nitrate (R17) 127
$Al(NO_3)_3.9H_2O$ (R16) 128
$LiNO_3$ (R22) 129
$Bi_5O(OH)_9(NO_3)_4$ (R32) 130
Cerium ammonium nitrate (R41) 131
$Bi(NO_3)_3$ (R30) 133
$Ni(NO_3)_2.6H_2O$ (R42) 134
Ethyl ammonium nitrate (R25) 135
3-methyl-1-sulfonic acid imidazolium nitrate (R37) 136
Thiourea Nitrate (R39) 137
Pyridinium Nitrate (R38) 138
$Y(NO_3)_3.6H_2O$ (R40) 139
Nitro methane (R36) 140
3-(Ethoxycarbonyl)-1-(5-methyl-5-(nitrosooxy)hexyl)pyridin-1-ium bis(trifluoromethanesulfonyl)imides (R27) 141
5-methyl-1,4-dinitroimidazole (R26) 142
2-methoxymethyl nitrite (R33) 144
Dinitro-5,5-dimethylhydantoin (R34) 145
Benziodazole-Type O_2NO-I(III) (R35) 146

II. EXTENDED ANALYSIS FOR THE AROMATIC NITRATION: 147

I. Performance score

All the research was based on published papers cited throughout the manuscript, each focusing on a specific type of nitration reaction. The information was extracted directly from these sources and subsequently organized according to reaction type: aromatic, *ipso*-, olefin-, heteroatom-, and alkyne nitration. These papers were then analyzed with respect to the reagents employed, activation modes, and scope examples.

General scoring system used:

- **Average Scope**: The number of successfully nitrated substrates was counted for each reagent, and an average was calculated. This metric provides an objective measure of how broadly a nitrating reagent has been applied, helping chemists assess its general usefulness across different structural classes. This score was then converted to a 10-point scale.
- **Electronic compatibility**: The types of substrates nitrated (electron-rich, electron-poor, neutral aromatics, and heteroaromatics) were evaluated to determine the breadth of applicability. This measure reflects the ability of a nitration method to accommodate a wide range of electronic environments, indicating its utility across diverse synthetic challenges. This score was then converted to a 10-point scale.
- **Average Yield**: Similarly, average yields across reported examples were compiled. Yield remains a central criterion in evaluating reaction efficiency; comparing average yields across reagents offers insight into which methods consistently deliver synthetically valuable results. This score was then converted to a 10-point scale.
- **Functional Group Tolerance**: Substrate scopes were analyzed for the presence of sensitive functional groups, such as alcohols, amines, amides, nitriles, carboxylic acids, esters, aldehydes, phenols, anilines, and various heterocycles. This parameter is crucial for determining the chemoselectivity and practicality of a nitration method in complex molecule synthesis, especially in late-stage functionalization or medicinal chemistry. 1 point for each functional group tolerated in the scope. The average score across all functional groups was used as the final tolerance value. This score was then converted to a 10-point scale.
- **Versatility**: Each reagent was assessed based on the number of distinct mechanisms it participates in and the diversity of nitrating species it can generate. Versatile reagents offer flexibility in synthetic planning by enabling multiple pathways or reactivities, making them valuable tools in a chemist's repertoire. This score was then converted to a 10-point scale.
- **Resource & Hazard:** A score was established based on reagent toxicity, cost, and the potential for recovery or recycling. Toxicity was scored as non-toxic = 2, unknown = 1, and toxic = 0. Recyclability contributed 1 point if applicable, otherwise 0. Cost was evaluated per gram, with 0–50 $ = 2 points, 50–200 $ or not commercially available = 1 point, and >200 $ = 0 points. Incorporating these parameters enables practitioners to evaluate reagents beyond intrinsic reactivity metrics, thereby facilitating more informed selection based on practical, economic, and environmental considerations. This score was then converted to a 10-point scale.

Final reagent score: for a given reagent, the average of the above values was taken and used to build the final reagent score.

1. *Ipso*-Nitration

Electronic compatibility (Total = 5 points)

1. Heteroaromatics (1 point)
    - Presence of at least one nitrated heteroaromatic substrate → 1 point, otherwise 0.
2. Electronic Classes (4 points total)
   For each electronic class (electron-rich, electron-poor, electron-neutral), points were assigned based on the proportion of substrates represented in the scope:
    - ≥25% → 2 points
    - 15–25% → 1 point
    - 0–15% → 0 points

   Electronic class definitions:
   - Electron-rich: anilines, OMe, ≥2 halogens, alcohols, *t*Bu
   - Electron-poor: carbonyl, cyano, boronic acid, OCF_3, CF_3
   - Electron-neutral: 1 halogen, alkyl, unsubstituted benzene, alkyne-substituted aromatics

Versatility (Total = 6 points)

Versatility was evaluated based on the number of mechanistic pathways accessible to the method and the diversity of nitrating species detected.

- 1–2 mechanisms → 1 point
- 3–4 mechanisms → 2 points
- ≥4 mechanisms → 3 points

Additionally, 1 point was awarded for each distinct nitrating species observed (e.g., Nitronium ion, nitryl radical, nitrite) up to a maximum of 3 points, giving a total possible versatility score of 6 points.

$NaNO_2$ (R3)

	Functional group tolerance												Average yield	Total Scope
**Ref.**	**Halo.**	**Alco.**	**Amine**	**Amide**	**Cyano**	**Acid**	**Ester**	**Aldehyde**	**Phenol**	**aniline**	**Hetero.**	**Score FG (%)**	**60.82**	40
	1	0	0	0	0	1	1	1	1	1	0	55		
**20**	**Electronic compatibility**							**Versatility**						
	**Rich**	**Poor**	**Neut.**					**Rad.**	**Elect.**	**Nucleo.**				
	12/40	13/40	15/40							x				
	**Functional group tolerance**												**Average yield**	**Total Scope**
**Ref.**	**Halo.**	**Alco.**	**Amine**	**Amide**	**Cyano**	**Acid**	**Ester**	**Aldehyde**	**Phenol**	**aniline**	**Hetero.**	**Score FG (%)**	65.19	36
	1	0	0	0	1	0	1	1	0	0	1	45		
**53**	**Electronic compatibility**							**Versatility**						
	**Rich**	**Poor**	**Neut.**					**Rad.**	**Elect.**	**Nucleo.**				
	6/36	10/36	20/36							x				
	**Functional group tolerance**												**Average yield**	**Total Scope**
**Ref.**	**Halo.**	**Alco.**	**Amine**	**Amide**	**Cyano**	**Acid**	**Ester**	**Aldehyde**	**Phenol**	**aniline**	**Hetero.**	**Score FG (%)**	70.73	11

**49**	1	0	0	0	0	0	1	0	0	0	0	18		
	**Electronic compatibility**							**Versatility**						
	**Rich**	**Poor**	**Neut.**					**Rad.**	**Elect.**	**Nucleo.**				
	2/11	3/11	6/11							x				
	**Functional group tolerance**												**Average yield**	**Total Scope**
**Ref.**	**Halo.**	**Alco.**	**Amine**	**Amide**	**Cyano**	**Acid**	**Ester**	**Aldehyde**	**Phenol**	**aniline**	**Hetero.**	**Score FG (%)**	87.63	19
**54**	1	0	0	0	1	0	0	1	0	0	1	36		
	**Electronic compatibility**							**Versatility**						
	**Rich**	**Poor**	**Neut.**					**Rad.**	**Elect.**	**Nucleo.**				
	1/19	4/19	14/19					x						
	**Functional group tolerance**												**Average yield**	**Total Scope**
**Ref.**	**Halo.**	**Alco.**	**Amine**	**Amide**	**Cyano**	**Acid**	**Ester**	**Aldehyde**	**Phenol**	**aniline**	**Hetero.**	**Score FG (%)**	70.0	10
**19**	1	0	0	0	1	0	0	1	0	0	0	27		
	**Electronic compatibility**							**Versatility**						
	**Rich**	**Poor**	**Neut.**					**Rad.**	**Elect.**	**Nucleo.**				
	2/10	3/10	5/10							x				

	**Functional group tolerance**											**Average yield**	**Total Scope**
**Ref.**	**Halo.**	**Alco.**	**Amine**	**Amide**	**Cyano**	**Acid**	**Ester**	**Aldehyde**	**Phenol**	**aniline**	**Hetero.**	**Score FG (%)**	38.79	14
**26**	1	0	0	0	0	0	0	0	0	0	0	9		
	**Electronic compatibility**							**Versatility**						
	**Rich**	**Poor**	**Neut.**					**Rad.**	**Elect.**	**Nucleo.**				
	7/14	2/14	5/14							x				

	**Functional group tolerance**												**Average yield**	**Total Scope**
**Ref.**	**Halo.**	**Alco.**	**Amine**	**Amide**	**Cyano**	**Acid**	**Ester**	**Aldehyde**	**Phenol**	**aniline**	**Hetero.**	**Score FG (%)**	70.24	49
**18**	0	1	0	0	1	0	0	0	0	1	0	27		
	**Electronic compatibility**							**Versatility**						
	**Rich**	**Poor**	**Neut.**					**Rad.**	**Elect.**	**Nucleo.**				
	17/49	4/49	28/49							x				

	**Functional group tolerance**												**Average yield**	**Total Scope**
**Ref.**	**Halo.**	**Alco.**	**Amine**	**Amide**	**Cyano**	**Acid**	**Ester**	**Aldehyde**	**Phenol**	**aniline**	**Hetero.**	**Score FG (%)**	86.07	14
	1	1	0	0	1	0	0	0	0	1	1	27		

17	Electronic compatibility							Versatility						
	**Rich**	**Poor**	**Neut.**					**Rad.**	**Elect.**	**Nucleo.**				
	3/14	2/14	9/14							x				

Average scope: 24.1 or 2.4/10
Electronic compatibility: 3/5 or 6/10
Average yield: 68.7% or 6.9/10
Versatility: 5/6 or 8.3/10
Resource & health: Toxic + price (50-200$/g) + not recyclable = 1/5 or 2/10
Functional group tolerance: 3.3/10

Average score: 4.8/10

***N*-Nitrosuccinimide (R12)**

	Functional group tolerance												Average yield	Total Scope
**Ref.**	**Halo.**	**Alco.**	**Amine**	**Amide**	**Cyano**	**Acid**	**Ester**	**Aldehyde**	**Phenol**	**aniline**	**Hetero.**	**Score FG (%)**	**77.25**	51
**38**	1	0	0	1	0	0	1	0	1	0	1	45		
	**Electronic compatibility**							**Versatility**						
	**Rich**	**Poor**	**Neut.**					**Rad.**	**Elect.**	**Nucleo.**				
	7/51	9/51	36/51					x						
	**Functional group tolerance**												**Average yield**	**Total Scope**
**Ref.**	**Halo.**	**Alco.**	**Amine**	**Amide**	**Cyano**	**Acid**	**Ester**	**Aldehyde**	**Phenol**	**aniline**	**Hetero.**	**Score FG (%)**	71.84	25
**39**	1	0	0	0	1	0	1	0	0	0	1	36		
	**Electronic compatibility**							**Versatility**						
	**Rich**	**Poor**	**Neut.**					**Rad.**	**Elect.**	**Nucleo.**				
	5/25	0/25	20/25					X						

Average scope: 38.0 or 3.8/10
Electronic compatibility: 2/5 or 4/10
Average yield: 74.5% or 7.5/10
Versatility: 2/6 or 3.3/10
Resource & health: Not toxic + price (>200$/g) + Recyclable = 3/5 or 6/10
Functional group tolerance: 4.1/10
Average score: 4.8/10

KNO_2 (R2)

	Functional group tolerance												Average yield	Total Scope
**Ref.**	**Halo.**	**Alco.**	**Amine**	**Amide**	**Cyano**	**Acid**	**Ester**	**Aldehyde**	**Phenol**	**aniline**	**Hetero.**	**Score FG (%)**	**64.88**	56
**31**	1	0	0	1	1	0	1	1	1	0	1	64		
	**Electronic compatibility**							**Versatility**						
	**Rich**	**Poor**	**Neut.**					**Rad.**	**Elect.**	**Nucleo.**				
	11/56	11/56	34/56							x				

	Functional group tolerance												Average yield	Total Scope
**Ref.**	**Halo.**	**Alco.**	**Amine**	**Amide**	**Cyano**	**Acid**	**Ester**	**Aldehyde**	**Phenol**	**aniline**	**Hetero.**	**Score FG (%)**	66.25	24
**22**	1	0	0	0	1	0	0	1	0	1	1	45		
	**Electronic compatibility**							**Versatility**						
	**Rich**	**Poor**	**Neut.**					**Rad.**	**Elect.**	**Nucleo.**				
	6/24	7/24	11/24							X				

Average scope: 40.0 or 4/10
Electronic compatibility: 3/5 or 6/10
Average yield: 65.6% or 6.6/10
Versatility: 2/6 or 3.3/10
Resource & health: Toxic + price (0-50$/g) + Not recyclable = 3/5 or 6/10
Functional group tolerance: 5.5/10
Average score: 4.9/10

HNO_3 (R8)

	Functional group tolerance												Average yield	Total Scope
**Ref.**	**Halo.**	**Alco.**	**Amine**	**Amide**	**Cyano**	**Acid**	**Ester**	**Aldehyde**	**Phenol**	**aniline**	**Hetero.**	**Score FG (%)**	**79.29**	**21**
**28**	1	1	0	1	1	1	0	0	0	0	0	45		
	**Electronic compatibility**							**Versatility**						
	**Rich**	**Poor**	**Neut.**					**Rad.**	**Elect.**	**Nucleo.**				
	1/21	4/21	16/21					x						
	**Functional group tolerance**												**Average yield**	**Total Scope**
**Ref.**	**Halo.**	**Alco.**	**Amine**	**Amide**	**Cyano**	**Acid**	**Ester**	**Aldehyde**	**Phenol**	**aniline**	**Hetero.**	**Score FG (%)**	59.47	15
**47**	1	0	0	0	0	0	1	0	0	0	0	18		
	**Electronic compatibility**							**Versatility**						
	**Rich**	**Poor**	**Neut.**					**Rad.**	**Elect.**	**Nucleo.**				
	2/15	3/15	10/15						x					

Average scope: 18 or 1.8/10
Electronic compatibility: 1/5 or 2/10
Average yield: 69.4% or 6.9/10
Versatility: 3/6 or 5/10
Resource & health: Toxic + price (0-50$/g) + Not recyclable = 2/5 or 4/10
Functional group tolerance: 3.2/10
Average score: 3.8/10

$Fe(NO_3)_3.9H_2O$ (R1)

	Functional group tolerance												Average yield	Total Scope
Ref.	Halo.	Alco.	Amine	Amide	Cyano	Acid	Ester	Aldehyde	Phenol	aniline	Hetero.	Score FG (%)	**67.50**	**92**
32	1	0	0	1	1	1	1	0	1	1	1	73		
	Electronic compatibility							Versatility						
	Rich	Poor	Neut.					Rad.	Elect.	Nucleo.				
	8/92	29/92	55/92					x						

	Functional group tolerance												Average yield	Total Scope
Ref.	Halo.	Alco.	Amine	Amide	Cyano	Acid	Ester	Aldehyde	Phenol	aniline	Hetero.	Score FG (%)	75.79	86
48	1	0	0	1	1	1	1	1	1	1	1	82		
	Electronic compatibility							Versatility						
	Rich	Poor	Neut.					Rad.	Elect.	Nucleo.				
	9/86	39/86	42/86					x						

	Functional group tolerance												Average yield	Total Scope
Ref.	Halo.	Alco.	Amine	Amide	Cyano	Acid	Ester	Aldehyde	Phenol	aniline	Hetero.	Score FG (%)	79.09	22

	1	0	0	0	0	1	1	1	1	1	0	55		
**40**	**Electronic compatibility**							**Versatility**						
	**Rich**	**Poor**	**Neut.**					**Rad.**	**Elect.**	**Nucleo.**				
	5/22	6/22	11/22					x						
	**Functional group tolerance**												**Average yield**	**Total Scope**
**Ref.**	**Halo.**	**Alco.**	**Amine**	**Amide**	**Cyano**	**Acid**	**Ester**	**Aldehyde**	**Phenol**	**aniline**	**Hetero.**	**Score FG (%)**	84.25	12
	1	0	0	0	1	0	0	0	0	0	1	27		
**29**	**Electronic compatibility**							**Versatility**						
	**Rich**	**Poor**	**Neut.**					**Rad.**	**Elect.**	**Nucleo.**				
	2/12	2/12	8/12						x					

Average scope: 53 or 5.3/10
Electronic compatibility: 3/5 or 6/10
Average yield: 76.7% or 7.7/10
Versatility: 3/6 or 5/10
Resource & health: Not toxic + price (0-50$/g) + Not recyclable = 4/5 or 8/10
Functional group tolerance: 5.9/10

Average score: 6.3/10

***Tetra*-butyl ammonium nitrite/nitrate (R10/R11)**

	**Functional group tolerance**												**Average yield**	**Total Scope**
**Ref.**	**Halo.**	**Alco.**	**Amine**	**Amide**	**Cyano**	**Acid**	**Ester**	**Aldehyde**	**Phenol**	**aniline**	**Hetero.**	**Score FG (%)**	**61.18**	**11**
	0	0	0	0	0	0	0	0	0	1	0	9		
**23**	**Electronic compatibility**							**Versatility**						
	**Rich**	**Poor**	**Neut.**					**Rad.**	**Elect.**	**Nucleo.**				
	6/11	0/11	5/11							x				
	**Functional group tolerance**												**Average yield**	**Total Scope**
**Ref.**	**Halo.**	**Alco.**	**Amine**	**Amide**	**Cyano**	**Acid**	**Ester**	**Aldehyde**	**Phenol**	**aniline**	**Hetero.**	**Score FG (%)**	60.94	16
	1	0	0	0	1	0	1	1	0	0	1	45		
**19**	**Electronic compatibility**							**Versatility**						
	**Rich**	**Poor**	**Neut.**					**Rad.**	**Elect.**	**Nucleo.**				
	1/16	4/16	11/16							x				
	**Functional group tolerance**												**Average yield**	**Total Scope**
**Ref.**	**Halo.**	**Alco.**	**Amine**	**Amide**	**Cyano**	**Acid**	**Ester**	**Aldehyde**	**Phenol**	**aniline**	**Hetero.**	**Score FG (%)**	54.46	13

**24**	1	0	0	1	0	0	0	0	0	1	0	27		
	**Electronic compatibility**							**Versatility**						
	**Rich**	**Poor**	**Neut.**					**Rad.**	**Elect.**	**Nucleo.**				
	6/13	3/13	4/13							x				

	**Functional group tolerance**												**Average yield**	**Total Scope**
**Ref.**	**Halo.**	**Alco.**	**Amine**	**Amide**	**Cyano**	**Acid**	**Ester**	**Aldehyde**	**Phenol**	**aniline**	**Hetero.**	**Score FG (%)**	39.31	16
**27**	1	0	0	0	0	0	0	0	0	0	1	18		
	**Electronic compatibility**							**Versatility**						
	**Rich**	**Poor**	**Neut.**					**Rad.**	**Elect.**	**Nucleo.**				
	3/16	0/16	13/16						x					

Average scope: 14 or 1.4/10
Electronic compatibility: 3/5 or 6/10
Average yield: 54% or 5.4/10
Versatility: 4/6 or 6.6/10
Resource & health: Not toxic + price (0-50$/g) + Not recyclable = 4/5 or 8/10
Functional group tolerance: 2.5/10

Average score: 5.0/10

***N*-Nitrosaccharin (R9)**

	**Functional group tolerance**												**Average yield**	**Total Scope**
**Ref.**	**Halo.**	**Alco.**	**Amine**	**Amide**	**Cyano**	**Acid**	**Ester**	**Aldehyde**	**Phenol**	**aniline**	**Hetero.**	**Score FG (%)**	**69.98**	**52**
**35**	1	0	1	1	0	1	1	1	1	1	1	82		
	**Electronic compatibility**							**Versatility**						
	**Rich**	**Poor**	**Neut.**					**Rad.**	**Elect.**	**Nucleo.**				
	8/52	13/52	31/52						X					

Average scope: 52 or 5.2/10
Electronic compatibility: 4/5 or 8/10
Average yield: 70% or 7.0/10
Versatility: 2/6 or 3.3/10
Resource & health: Not toxic + price (>200$/g) + Recyclable = 3/5 or 6/10
Functional group tolerance: 8.2/10

Average score: 6.3/10

$Bi(NO_3)_3.5H_2O$ (R5)

	Functional group tolerance												Average yield	Total Scope
**Ref.**	**Halo.**	**Alco.**	**Amine**	**Amide**	**Cyano**	**Acid**	**Ester**	**Aldehyde**	**Phenol**	**aniline**	**Hetero.**	**Score FG (%)**	**74.07**	**27**
**44**	1	0	0	0	0	0	1	0	0	0	1	27		
	**Electronic compatibility**							**Versatility**						
	**Rich**	**Poor**	**Neut.**					**Rad.**	**Elect.**	**Nucleo.**				
	4/27	3/27	20/27					x						

	Functional group tolerance												Average yield	Total Scope
**Ref.**	**Halo.**	**Alco.**	**Amine**	**Amide**	**Cyano**	**Acid**	**Ester**	**Aldehyde**	**Phenol**	**aniline**	**Hetero.**	**Score FG (%)**	76.47	15
**43**	0	0	0	0	0	0	0	1	1	0	1	27		
	**Electronic compatibility**							**Versatility**						
	**Rich**	**Poor**	**Neut.**					**Rad.**	**Elect.**	**Nucleo.**				
	1/15	2/15	12/15						x					

	Functional group tolerance												Average yield	Total Scope
**Ref.**	**Halo.**	**Alco.**	**Amine**	**Amide**	**Cyano**	**Acid**	**Ester**	**Aldehyde**	**Phenol**	**aniline**	**Hetero.**	**Score FG (%)**	32.50	12

	1	0	0	0	0	0	0	0	0	0	1	18		
**42**	**Electronic compatibility**							**Versatility**						
	**Rich**	**Poor**	**Neut.**					**Rad.**	**Elect.**	**Nucleo.**				
	0/12	0/12	12/12					x						

Average scope: 18 or 1.8/10
Electronic compatibility: 1/5 or 2/10
Average yield: 61% or 6.1/10
Versatility: 3/6 or 5/10
Resource & health: Not toxic + price (>200$/g) + Not recyclable = 4/5 or 8/10
Functional group tolerance: 2.4/10

Average score: 4.2/10

NO_2BF_4 (R6)

	Functional group tolerance												Average yield	Total Scope
**Ref.**	**Halo.**	**Alco.**	**Amine**	**Amide**	**Cyano**	**Acid**	**Ester**	**Aldehyde**	**Phenol**	**aniline**	**Hetero.**	**Score FG (%)**	**82.71**	**21**
**51**	1	0	0	0	1	0	1	1	0	0	1	45		
	**Electronic compatibility**							**Versatility**						
	**Rich**	**Poor**	**Neut.**					**Rad.**	**Elect.**	**Nucleo.**				
	1/21	5/21	15/21						x					

	Functional group tolerance												Average yield	Total Scope
**Ref.**	**Halo.**	**Alco.**	**Amine**	**Amide**	**Cyano**	**Acid**	**Ester**	**Aldehyde**	**Phenol**	**aniline**	**Hetero.**	**Score FG (%)**	63.68	25
**50**	1	0	0	0	0	0	0	0	1	0	1	27		
	**Electronic compatibility**							**Versatility**						
	**Rich**	**Poor**	**Neut.**					**Rad.**	**Elect.**	**Nucleo.**				
	2/25	3/25	20/25						x					

Average scope: 23 or 2.3/10
Electronic compatibility: 2/5 or 4/10
Average yield: 73% or 7.3/10
Versatility: 2/6 or 3.3/10
Resource & health: Toxic + price (>200$/g) + Not recyclable = 2/5 or 4/10
Functional group tolerance: 3.6/10
Average score: 4.1/10

1,3-disulfonic acid imidazolium nitrate (R14)

	Functional group tolerance												Average yield	Total Scope
**Ref.**	**Halo.**	**Alco.**	**Amine**	**Amide**	**Cyano**	**Acid**	**Ester**	**Aldehyde**	**Phenol**	**aniline**	**Hetero.**	**Score FG (%)**	**83.08**	**13**
**52**	1	0	0	0	0	0	0	1	1	0	0	27		
	**Electronic compatibility**							**Versatility**						
	**Rich**	**Poor**	**Neut.**					**Rad.**	**Elect.**	**Nucleo.**				
	3/13	4/13	6/13					X						

Average scope: 13 or 1.3/10
Electronic compatibility: 1/5 or 2/10
Average yield: 83% or 8.3/10
Versatility: 2/6 or 3.3/10
Resource & health: Unknown + price (>200$/g) + Recyclable = 2/5 or 4/10
Functional group tolerance: 2.7/10

Average score: 3.6/10

$ZrO(NO_3)$. xH_2O (R4)

	Functional group tolerance												Average yield	Total Scope
Ref.	Halo.	Alco.	Amine	Amide	Cyano	Acid	Ester	Aldehyde	Phenol	aniline	Hetero.	Score FG (%)	84.41	17
30	1	0	0	0	0	0	0	1	1	1	0	36		
	Electronic compatibility							Versatility						
	Rich	Poor	Neut.					Rad.	Elect.	Nucleo.				
	3/17	2/17	12/17						x					

Average scope: 17 or 1.7/10
Electronic compatibility: 1/5 or 2/10
Average yield: 84.4% or 8.4/10
Versatility: 2/6 or 3.3/10
Resource & health: Not toxic + price (0-50$/g) + Not recyclable = 4/5 or 8/10
Functional group tolerance: 3.6/10

Average score: 4.5/10

***Tert*-butyl nitrite (R13)**

	Functional group tolerance												Average yield	Total Scope
**Ref.**	**Halo.**	**Alco.**	**Amine**	**Amide**	**Cyano**	**Acid**	**Ester**	**Aldehyde**	**Phenol**	**aniline**	**Hetero.**	**Score FG (%)**	**68.40**	**10**
**46**	1	0	0	0	0	0	0	1	0	0	0	18		
	**Electronic compatibility**							**Versatility**						
	**Rich**	**Poor**	**Neut.**					**Rad.**	**Elect.**	**Nucleo.**				
	2/10	1/10	7/10							x				

	Functional group tolerance												Average yield	Total Scope
**Ref.**	**Halo.**	**Alco.**	**Amine**	**Amide**	**Cyano**	**Acid**	**Ester**	**Aldehyde**	**Phenol**	**aniline**	**Hetero.**	**Score FG (%)**	61.0	13
**45**	1	0	0	0	1	0	0	0	0	0	0	18		
	**Electronic compatibility**							**Versatility**						
	**Rich**	**Poor**	**Neut.**					**Rad.**	**Elect.**	**Nucleo.**				
	3/13	2/13	8/13						x					

Average scope: 11.5 or 1.2/10
Electronic compatibility: 1/5 or 2/10
Average yield: 64.7% or 6.5/10
Versatility: 2/6 or 3.3/10
Resource & health: Not toxic + price (0-50$/g) + Not recyclable = 4/5 or 8/10
Functional group tolerance: 1.8/10
Average score: 3.8/10

$AgNO_3$ (R7)

	Functional group tolerance												Average yield	Total Scope
**Ref.**	**Halo.**	**Alco.**	**Amine**	**Amide**	**Cyano**	**Acid**	**Ester**	**Aldehyde**	**Phenol**	**aniline**	**Hetero.**	**Score FG (%)**	**43.22**	**9**
**41**	1	0	0	0	0	0	0	0	0	1	0	18		
	**Electronic compatibility**							**Versatility**						
	**Rich**	**Poor**	**Neut.**					**Rad.**	**Elect.**	**Nucleo.**				
	9/9	0/9	0/9						x					

Average scope: 9 or 0.9/10
Electronic compatibility: 1/5 or 2/10
Average yield: 43.2% or 4.3/10
Versatility: 2/6 or 3.3/10
Resource & health: Toxic + price (50-200$/g) + Not recyclable = 1/5 or 2/10
Functional group tolerance: 3.6/10

Average score: 2.4/10

2. Alkyne Nitration

Electronic compatibility (Total = 5 points)

1. Difunctionalization (1 point)
    - Method allows difunctionalization → 1 point
    - Otherwise → 0 points
2. Alkyne Type (3 points total)
    Points assigned based on the proportion of phenylacetylene substrates in the scope:
    - 75% → 1 point
    - 50–75% → 2 points
    - <50% → 3 points

    Terminal acetylenes:
    - Included → 1 point
    - Not included → 0 points

Versatility (Total = 6 points)

Versatility was evaluated based on the number of mechanistic pathways accessible to the method and the diversity of nitrating species detected.

- 1 mechanism → 1 point
- 2 mechanisms → 2 points
- ≥3 mechanisms → 3 points

Additionally, 1 point was awarded for each distinct nitrating species observed (e.g., Nitronium ion, nitryl radical, nitrite) up to a maximum of 3 points, giving a total possible versatility score of 6 points.

$Fe(NO_3)_3.9H_2O$ (R1)

	Functional group tolerance												Average yield	Total Scope
Ref.	Halo.	Alco.	Amine	Amide	Cyano	Acid	Ester	Aldehyde	Phenol	aniline	Hetero.	Score FG (%)	44	3
71	0	0	0	0	0	0	0	0	0	0	0	0		
	Electronic compatibility							Versatility						
	Phen.	Acety.	Difun.	Ter. Alky.				Rad.	Elect.	Nucleo.				
	2/3	1/3	X	X				x						

	Functional group tolerance												Average yield	Total Scope
Ref.	Halo.	Alco.	Amine	Amide	Cyano	Acid	Ester	Aldehyde	Phenol	aniline	Hetero.	Score FG (%)	55	14
56	1	0	0	0	1	0	0	0	0	0	0	20		
	Electronic compatibility							Versatility						
	Phen.	Acety.	Difun.	Ter. Alky.				Rad.	Elect.	Nucleo.				
	14/14	0/14	X					x						

Average scope: 8.5 or 0.9/10
Electronic compatibility: 3/5 or 6/10
Average yield: 49.5% or 5.0/10
Versatility: 4/6 or 6.6/10
Resource & health: Not toxic + price (0-50$/g) + Not recyclable = 4/5 or 8/10
Functional group tolerance: 1/10
Average score: 4.6/10

***Tert*-butyl nitrite (R13)**

	Functional group tolerance												Average yield	Total Scope
Ref.	Halo.	Alco.	Amine	Amide	Cyano	Acid	Ester	Aldehyde	Phenol	aniline	Hetero.	Score FG (%)	72.18	28
59	1	0	0	0	0	0	0	1	0	0	0	20		
	Electronic compatibility							Versatility						
	Phen.	Acety.	Difun.	Ter. Alky.				Rad.	Elect.	Nucleo.				
	28/28	0/28	X					x						

	Functional group tolerance												Average yield	Total Scope
Ref.	Halo.	Alco.	Amine	Amide	Cyano	Acid	Ester	Aldehyde	Phenol	aniline	Hetero.	Score FG (%)	70.81	27
60	1	0	0	1	1	0	0	0	0	0	0	30		
	Electronic compatibility							Versatility						
	Phen.	Acety.	Difun.	Ter. Alky.				Rad.	Elect.	Nucleo.				
	23/27	4/27	X	X				x						

	Functional group tolerance												Average yield	Total Scope
Ref.	Halo.	Alco.	Amine	Amide	Cyano	Acid	Ester	Aldehyde	Phenol	aniline	Hetero.	Score FG (%)	40.73	22

61	1	0	0	0	0	0	1	0	0	0	0	20		
	Electronic compatibility							Versatility						
	Phen.	Acety.	Difun.	Ter. Alky.				Rad.	Elect.	Nucleo.				
	22/22	0/22	X					x						
	Functional group tolerance												Average yield	Total Scope
Ref.	Halo.	Alco.	Amine	Amide	Cyano	Acid	Ester	Aldehyde	Phenol	aniline	Hetero.	Score FG (%)	77.05	20
62	1	0	0	0	1	0	1	1	0	0	0	40		
	Electronic compatibility							Versatility						
	Phen.	Acety.	Difun.	Ter. Alky.				Rad.	Elect.	Nucleo.				
	20/20	0/20						x						
	Functional group tolerance												Average yield	Total Scope
Ref.	Halo.	Alco.	Amine	Amide	Cyano	Acid	Ester	Aldehyde	Phenol	aniline	Hetero.	Score FG (%)	62.67	15
63	1	0	0	0	1	0	0	0	0	0	0	20		
	Electronic compatibility							Versatility						
	Phen.	Acety.	Difun.	Ter. Alky.				Rad.	Elect.	Nucleo.				
	14/15	1/15	X					x						

	Functional group tolerance												Average yield	Total Scope
Ref.	Halo.	Alco.	Amine	Amide	Cyano	Acid	Ester	Aldehyde	Phenol	aniline	Hetero.	Score FG (%)	65.06	18
64	1	0	0	1	1	0	0	0	0	0	0	30		
	Electronic compatibility							Versatility						
	Phen.	Acety.	Difun.	Ter. Alky.				Rad.	Elect.	Nucleo.				
	18/18	0/15	X					x						
	Functional group tolerance												Average yield	Total Scope
Ref.	Halo.	Alco.	Amine	Amide	Cyano	Acid	Ester	Aldehyde	Phenol	aniline	Hetero.	Score FG (%)	64.84	19
55	1	0	0	0	0	0	0	0	0	0	0	10		
	Electronic compatibility							Versatility						
	Phen.	Acety.	Difun.	Ter. Alky.				Rad.	Elect.	Nucleo.				
	19/19	0/19	X	X				x						
	Functional group tolerance												Average yield	Total Scope
Ref.	Halo.	Alco.	Amine	Amide	Cyano	Acid	Ester	Aldehyde	Phenol	aniline	Hetero.	Score FG (%)	62.73	22
	1	0	0	0	0	0	0	0	0	0	0	10		

**65**	**Electronic compatibility**							**Versatility**						
	**Phen.**	**Acety.**	**Difun.**	**Ter. Alky.**				**Rad.**	**Elect.**	**Nucleo.**				
	22/22	0/22	X					x						
	**Functional group tolerance**												**Average yield**	**Total Scope**
**Ref.**	**Halo.**	**Alco.**	**Amine**	**Amide**	**Cyano**	**Acid**	**Ester**	**Aldehyde**	**Phenol**	**aniline**	**Hetero.**	**Score FG (%)**	**61.11**	**19**
**65**	1	0	0	0	0	0	0	0	0	0	0	10		
	**Electronic compatibility**							**Versatility**						
	**Phen.**	**Acety.**	**Difun.**	**Ter. Alky.**				**Rad.**	**Elect.**	**Nucleo.**				
	19/19	0/19	X					x						

Average scope: 21.1 or 2.1/10
Electronic compatibility: 3/5 or 6/10
Average yield: 65% or 6.5/10
Versatility: 2/6 or 3.3/10
Resource & health: Not toxic + price (0-50$/g) + Not recyclable = 4/5 or 8/10
Functional group tolerance: 2.1/10

Average score: 4.7/10

$NaNO_2$ (R3)

	Functional group tolerance												Average yield	Total Scope
Ref.	Halo.	Alco.	Amine	Amide	Cyano	Acid	Ester	Aldehyde	Phenol	aniline	Hetero.	Score FG (%)	51.56	9
67	1	0	0	0	0	0	0	0	0	0	0	10		
	Electronic compatibility							Versatility						
	Phen.	Acety.	Difun.	Ter. Alky.				Rad.	Elect.	Nucleo.				
	9/9	0/9	X	X				x						

	Functional group tolerance												Average yield	Total Scope
Ref.	Halo.	Alco.	Amine	Amide	Cyano	Acid	Ester	Aldehyde	Phenol	aniline	Hetero.	Score FG (%)	74.50	22
72	1	0	0	0	1	0	0	0	0	0	0	20		
	Electronic compatibility							Versatility						
	Phen.	Acety.	Difun.	Ter. Alky.				Rad.	Elect.	Nucleo.				
	22/22	0/22	X					x						

	Functional group tolerance												Average yield	Total Scope
Ref.	Halo.	Alco.	Amine	Amide	Cyano	Acid	Ester	Aldehyde	Phenol	aniline	Hetero.	Score FG (%)	79.08	26

	1	0	0	0	0	0	0	0	0	0	0	10		
**73**	**Electronic compatibility**							**Versatility**						
	**Phen.**	**Acety.**	**Difun.**	**Ter. Alky.**				**Rad.**	**Elect.**	**Nucleo.**				
	26/26	0/26	X					x						

Average scope: 19.0 or 1.9/10
Electronic compatibility: 3/5 or 6/10
Average yield: 68.4% or 6.8/10
Versatility: 3/6 or 5/10
Resource & health: Toxic + price (50-200$/g) + Not recyclable = 1/5 or 2/10
Functional group tolerance: 1.3/10

Average score: 3.8/10

$AgNO_3$ (R7)

	Functional group tolerance												Average yield	Total Scope
**Ref.**	**Halo.**	**Alco.**	**Amine**	**Amide**	**Cyano**	**Acid**	**Ester**	**Aldehyde**	**Phenol**	**aniline**	**Hetero.**	**Score FG (%)**	**54.07**	**14**
**68**	1	0	0	0	0	0	0	0	0	0	0	10		
	**Electronic compatibility**							**Versatility**						
	**Phen.**	**Acety.**	**Difun.**	**Ter. Alky.**				**Rad.**	**Elect.**	**Nucleo.**				
	14/14	0/14	X					x						

Average scope: 14 or 1.4/10
Electronic compatibility: 2/5 or 4/10
Average yield: 54.1% or 5.4/10
Versatility: 2/6 or 3.3/10
Resource & health: Toxic + price (50-200$/g) + Not recyclable = 1/5 or 2/10
Functional group tolerance: 1/10

Average score: 2.9/10

$Al(NO_3)_3.9H_2O$ (R16)

	Functional group tolerance												Average yield	Total Scope
**Ref.**	**Halo.**	**Alco.**	**Amine**	**Amide**	**Cyano**	**Acid**	**Ester**	**Aldehyde**	**Phenol**	**aniline**	**Hetero.**	**Score FG (%)**	**48.60**	**20**
**58**	1	1	0	0	0	0	0	0	0	0	0	20		
	**Electronic compatibility**							**Versatility**						
	**Phen.**	**Acety.**	**Difun.**	**Ter. Alky.**				**Rad.**	**Elect.**	**Nucleo.**				
	20/20	0/20	X					x						

Average scope: 20 or 2/10
Electronic compatibility: 2/5 or 4/10
Average yield: 48.6% or 4.7/10
Versatility: 2/6 or 3.3/10
Resource & health: Toxic + price (0-50$/g) + Not recyclable = 2/5 or 4/10
Functional group tolerance: 2/10

Average score: 3.4/10

$Cu(NO_3)_2.3H_2O$ (R15)

	Functional group tolerance												Average yield	Total Scope
**Ref.**	**Halo.**	**Alco.**	**Amine**	**Amide**	**Cyano**	**Acid**	**Ester**	**Aldehyde**	**Phenol**	**aniline**	**Hetero.**	**Score FG (%)**	**76.05**	**21**
**57**	1	1	0	0	1	0	0	0	0	1	0	40		
	**Electronic compatibility**							**Versatility**						
	**Phen.**	**Acety.**	**Difun.**	**Ter. Alky.**				**Rad.**	**Elect.**	**Nucleo.**				
	18/21	3/21	X	X					x					

Average scope: 21 or 2.1/10
Electronic compatibility: 3/5 or 6/10
Average yield: 76% or 7.6/10
Versatility: 2/6 or 3.3/10
Resource & health: Not toxic + price (0-50$/g) + Not recyclable = 4/5 or 8/10
Functional group tolerance: 4/10

Average score: 5.2/10

Guanidine nitrate (R17)

	Functional group tolerance												Average yield	Total Scope
**Ref.**	**Halo.**	**Alco.**	**Amine**	**Amide**	**Cyano**	**Acid**	**Ester**	**Aldehyde**	**Phenol**	**aniline**	**Hetero.**	**Score FG (%)**	**50**	**4**
**69**	1	0	0	0	0	0	0	0	0	0	0	10		
	**Electronic compatibility**							**Versatility**						
	**Phen.**	**Acety.**	**Difun.**	**Ter. Alky.**				**Rad.**	**Elect.**	**Nucleo.**				
	4/4	0/4	X	X					x					

Average scope: 4 or 0.4/10
Electronic compatibility: 3/5 or 6/10
Average yield: 50% or 5/10
Versatility: 2/6 or 3.3/10
Resource & health: Not toxic + price (0-50$/g) + Not recyclable = 4/5 or 8/10
Functional group tolerance: 1/10

Average score: 4/10

HNO_3 (R8)

	Functional group tolerance												Average yield	Total Scope
**Ref.**	**Halo.**	**Alco.**	**Amine**	**Amide**	**Cyano**	**Acid**	**Ester**	**Aldehyde**	**Phenol**	**aniline**	**Hetero.**	**Score FG (%)**	**41.3**	**3**
**70**	0	0	0	0	0	0	0	0	0	0	0	0		
	**Electronic compatibility**							**Versatility**						
	**Phen.**	**Acety.**	**Difun.**	**Ter. Alky.**				**Rad.**	**Elect.**	**Nucleo.**				
	3/3	0/3	X	X					x					

Average scope: 3 or 0.3/10
Electronic compatibility: 3/5 or 6/10
Average yield: 41.3% or 4.1/10
Versatility: 2/6 or 3.3/10
Resource & health: Toxic + price (0-50$/g) + Not recyclable = 2/5 or 4/10
Functional group tolerance: 0/10

Average score: 3.0/10

3. Olefin Nitration

Electronic compatibility (Total = 5 points)

1. Difunctionalization (1 point)
   - Method allows difunctionalization → 1 point
   - Otherwise → 0 points
2. Olefin Type (3 points total)
   Points assigned based on the proportion of styrene substrates in the scope:
   - 75% → 1 point
   - 50–75% → 2 points
   - <50% → 3 points

   Terminal olefin:
   - Included → 1 point
   - Not included → 0 points

Versatility (Total = 6 points)

Versatility was evaluated based on the number of mechanistic pathways accessible to the method and the diversity of nitrating species detected.

- 1 mechanism → 1 point
- 2 mechanisms → 2 points
- ≥3 mechanisms → 3 points

Additionally, 1 point was awarded for each distinct nitrating species observed (e.g., Nitronium ion, nitryl radical, nitrite) up to a maximum of 3 points, giving a total possible versatility score of 6 points.

$Fe(NO_3)_3.9H_2O$ (R1)

	**Functional group tolerance**												**Average yield**	**Total Scope**
**Ref.**	**Halo.**	**Alco.**	**Amine**	**Amide**	**Cyano**	**Acid**	**Ester**	**Aldehyde**	**Phenol**	**aniline**		**Score FG (%)**	**74.45**	**65**
**71**	1	1	0	1	1	0	1	1	0	0		60		
	**Electronic compatibility**							**Versatility**						
	**Styr.**	**Olefin**	**Difun.**	**Ter. Alky.**				**Rad.**	**Elect.**	**Nucleo.**				
	48/65	17/65	X	X				x						
	**Functional group tolerance**												**Average yield**	**Total Scope**
**Ref.**	**Halo.**	**Alco.**	**Amine**	**Amide**	**Cyano**	**Acid**	**Ester**	**Aldehyde**	**Phenol**	**aniline**		**Score FG (%)**	**77.74**	**43**
**123**	1	0	1	0	0	1	1	1	0	0		50		
	**Electronic compatibility**							**Versatility**						
	**Styr.**	**Olefin**	**Difun.**	**Ter. Alky.**				**Rad.**	**Elect.**	**Nucleo.**				
	36/43	7/43	X	x				x						
	**Functional group tolerance**												**Average yield**	**Total Scope**
**Ref.**	**Halo.**	**Alco.**	**Amine**	**Amide**	**Cyano**	**Acid**	**Ester**	**Aldehyde**	**Phenol**	**aniline**		**Score FG (%)**	**54.58**	**37**

	1	0	0	0	0	0	0	0	0	0		10		
**77**	**Electronic compatibility**							**Versatility**						
	**Styr.**	**Olefin**	**Difun.**	**Ter. Alky.**				**Rad.**	**Elect.**	**Nucleo.**				
	37/37	0/37	X					x						
	**Functional group tolerance**												**Average yield**	**Total Scope**
**Ref.**	**Halo.**	**Alco.**	**Amine**	**Amide**	**Cyano**	**Acid**	**Ester**	**Aldehyde**	**Phenol**	**aniline**		**Score FG (%)**	**77.44**	**43**
	1	0	0	0	1	0	1	0	0	0		30		
**120**	**Electronic compatibility**							**Versatility**						
	**Styr.**	**Olefin**	**Difun.**	**Ter. Alky.**				**Rad.**	**Elect.**	**Nucleo.**				
	43/43	0/43		X				x						
	**Functional group tolerance**												**Average yield**	**Total Scope**
**Ref.**	**Halo.**	**Alco.**	**Amine**	**Amide**	**Cyano**	**Acid**	**Ester**	**Aldehyde**	**Phenol**	**aniline**		**Score FG (%)**	**75.56**	**36**
	1	0	0	0	1	0	1	1	0	0		40		
**76**	**Electronic compatibility**							**Versatility**						
	**Styr.**	**Olefin**	**Difun.**	**Ter. Alky.**				**Rad.**	**Elect.**	**Nucleo.**				
	27/36	9/36		X				x						

	**Functional group tolerance**												**Average yield**	**Total Scope**
**Ref.**	**Halo.**	**Alco.**	**Amine**	**Amide**	**Cyano**	**Acid**	**Ester**	**Aldehyde**	**Phenol**	**aniline**		**Score FG (%)**	**54.69**	**26**
**78**	1	0	0	1	0	0	1	0	0	0		30		
	**Electronic compatibility**							**Versatility**						
	**Styr.**	**Olefin**	**Difun.**	**Ter. Alky.**				**Rad.**	**Elect.**	**Nucleo.**				
	9/26	17/26	X	X				x						
	**Functional group tolerance**												**Average yield**	**Total Scope**
**Ref.**	**Halo.**	**Alco.**	**Amine**	**Amide**	**Cyano**	**Acid**	**Ester**	**Aldehyde**	**Phenol**	**aniline**		**Score FG (%)**	**62.08**	**13**
**79**	0	0	0	0	0	0	1	0	0	0		10		
	**Electronic compatibility**							**Versatility**						
	**Styr.**	**Olefin**	**Difun.**	**Ter. Alky.**				**Rad.**	**Elect.**	**Nucleo.**				
	0/13	13/13	X	X				x						
	**Functional group tolerance**												**Average yield**	**Total Scope**
**Ref.**	**Halo.**	**Alco.**	**Amine**	**Amide**	**Cyano**	**Acid**	**Ester**	**Aldehyde**	**Phenol**	**aniline**		**Score FG (%)**	**77.23**	**13**
	1	0	0	0	1	0	1	0	0	0		30		

**80**	**Electronic compatibility**							**Versatility**						
	**Styr.**	**Olefin**	**Difun.**	**Ter. Alky.**				**Rad.**	**Elect.**	**Nucleo.**				
	13/13	0/13						x						
	**Functional group tolerance**												**Average yield**	**Total Scope**
**Ref.**	**Halo.**	**Alco.**	**Amine**	**Amide**	**Cyano**	**Acid**	**Ester**	**Aldehyde**	**Phenol**	**aniline**		**Score FG (%)**	**65.21**	**19**
**81**	1	0	0	0	0	0	0	0	1	0		20		
	**Electronic compatibility**							**Versatility**						
	**Styr.**	**Olefin**	**Difun.**	**Ter. Alky.**				**Rad.**	**Elect.**	**Nucleo.**				
	19/19	0/19		X				x						
	**Functional group tolerance**												**Average yield**	**Total Scope**
**Ref.**	**Halo.**	**Alco.**	**Amine**	**Amide**	**Cyano**	**Acid**	**Ester**	**Aldehyde**	**Phenol**	**aniline**		**Score FG (%)**	**60.29**	**17**
**82**	0	1	0	1	0	0	0	0	0	0		20		
	**Electronic compatibility**							**Versatility**						
	**Styr.**	**Olefin**	**Difun.**	**Ter. Alky.**				**Rad.**	**Elect.**	**Nucleo.**				
	0/17	17/17	X	X				x						

Average scope: 31.2 or 3.1/10
Electronic compatibility: 4/5 or 8/10
Average yield: 67.9% or 6.8/10
Versatility: 4/6 or 6.6/10
Resource & health: Not toxic + price (0-50$/g) + Not recyclable = 4/5 or 8/10
Functional group tolerance: 3/10

Average score: 5.9/10

***Tetra*-butyl ammonium nitrate (R11)**

	**Functional group tolerance**												**Average yield**	**Total Scope**
**Ref.**	**Halo.**	**Alco.**	**Amine**	**Amide**	**Cyano**	**Acid**	**Ester**	**Aldehyde**	**Phenol**	**aniline**		**Score FG (%)**	**76.75**	**12**
	1	0	0	0	1	0	1	0	0	0		30		
**113**	**Electronic compatibility**							**Versatility**						
	**Styr.**	**Olefin**	**Difun.**	**Ter. Alky.**				**Rad.**	**Elect.**	**Nucleo.**				
	0/12	12/12							x					

	**Functional group tolerance**												**Average yield**	**Total Scope**
**Ref.**	**Halo.**	**Alco.**	**Amine**	**Amide**	**Cyano**	**Acid**	**Ester**	**Aldehyde**	**Phenol**	**aniline**		**Score FG (%)**	**64.58**	**12**
	0	0	0	0	0	0	0	0	0	0		0		
**112**	**Electronic compatibility**							**Versatility**						
	**Styr.**	**Olefin**	**Difun.**	**Ter. Alky.**				**Rad.**	**Elect.**	**Nucleo.**				
	2/12	10/12		x					x					

Average scope: 12 or 1.2/10
Electronic compatibility: 4/5 or 8/10
Average yield: 70.7% or 7.1/10
Versatility: 2/6 or 3.3/10
Resource & health: Not toxic + price (0-50$/g) + Not recyclable = 4/5 or 8/10
Functional group tolerance: 1.5/10
Average score: 5.9/10

***N*-Nitrosuccinimide (R12)**

	Functional group tolerance												Average yield	Total Scope
**Ref.**	**Halo.**	**Alco.**	**Amine**	**Amide**	**Cyano**	**Acid**	**Ester**	**Aldehyde**	**Phenol**	**aniline**		**Score FG (%)**	**71.44**	**25**
**109**	1	0	0	0	0	0	0	0	0	0		10		
	**Electronic compatibility**							**Versatility**						
	**Styr.**	**Olefin**	**Difun.**	**Ter. Alky.**				**Rad.**	**Elect.**	**Nucleo.**				
	25/25	0/25	X	X				X						

	Functional group tolerance												Average yield	Total Scope
**Ref.**	**Halo.**	**Alco.**	**Amine**	**Amide**	**Cyano**	**Acid**	**Ester**	**Aldehyde**	**Phenol**	**aniline**		**Score FG (%)**	**63.30**	**47**
**110**	1	0	0	1	1	0	1	1	0	0		50		
	**Electronic compatibility**							**Versatility**						
	**Styr.**	**Olefin**	**Difun.**	**Ter. Alky.**				**Rad.**	**Elect.**	**Nucleo.**				
	41/47	6/47	X	x				x						

	Functional group tolerance												Average yield	Total Scope
**Ref.**	**Halo.**	**Alco.**	**Amine**	**Amide**	**Cyano**	**Acid**	**Ester**	**Aldehyde**	**Phenol**	**aniline**		**Score FG (%)**	**63.03**	**49**

**108**	1	0	0	0	1	0	1	0	0	0		30		
	**Electronic compatibility**							**Versatility**						
	**Styr.**	**Olefin**	**Difun.**	**Ter. Alky.**				**Rad.**	**Elect.**	**Nucleo.**				
	49/49	0/49	X	X				X						
	**Functional group tolerance**												**Average yield**	**Total Scope**
**Ref.**	**Halo.**	**Alco.**	**Amine**	**Amide**	**Cyano**	**Acid**	**Ester**	**Aldehyde**	**Phenol**	**aniline**		**Score FG (%)**	**72.85**	**27**
**111**	1	0	0	1	0	0	0	0	0	0		20		
	**Electronic compatibility**							**Versatility**						
	**Styr.**	**Olefin**	**Difun.**	**Ter. Alky.**				**Rad.**	**Elect.**	**Nucleo.**				
	23/27	5/27	X	X				X						

Average scope: 37 or 3.7/10
Electronic compatibility: 3/5 or 6/10
Average yield: 67.7% or 6.8/10
Versatility: 2/6 or 3.3/10
Resource & health: Not toxic + price (>200$/g) + Recyclable = 3/5 or 6/10
Functional group tolerance: 2.8/10

Average score: 4.8/10

$Cu(NO_3)_2.3H_2O$ (R15)

	Functional group tolerance												Average yield	Total Scope
**Ref.**	**Halo.**	**Alco.**	**Amine**	**Amide**	**Cyano**	**Acid**	**Ester**	**Aldehyde**	**Phenol**	**aniline**		**Score FG (%)**	**74.74**	**39**
**84**	1	0	0	0	0	0	1	0	0	0		20		
	**Electronic compatibility**							**Versatility**						
	**Styr.**	**Olefin**	**Difun.**	**Ter. Alky.**				**Rad.**	**Elect.**	**Nucleo.**				
	0/39	39/39		X					x					
	**Functional group tolerance**												**Average yield**	**Total Scope**
**Ref.**	**Halo.**	**Alco.**	**Amine**	**Amide**	**Cyano**	**Acid**	**Ester**	**Aldehyde**	**Phenol**	**aniline**		**Score FG (%)**	**83.06**	**17**
**74**	1	0	0	0	0	0	0	0	0	0		10		
	**Electronic compatibility**							**Versatility**						
	**Styr.**	**Olefin**	**Difun.**	**Ter. Alky.**				**Rad.**	**Elect.**	**Nucleo.**				
	15/17	2/17	X	x				x						
	**Functional group tolerance**												**Average yield**	**Total Scope**
**Ref.**	**Halo.**	**Alco.**	**Amine**	**Amide**	**Cyano**	**Acid**	**Ester**	**Aldehyde**	**Phenol**	**aniline**		**Score FG (%)**	**69.92**	**26**

**114**	1	0	0	0	0	0	1	1	0	0		30		
	**Electronic compatibility**							**Versatility**						
	**Styr.**	**Olefin**	**Difun.**	**Ter. Alky.**				**Rad.**	**Elect.**	**Nucleo.**				
	19/26	7/26	X	X					X					
	**Functional group tolerance**												**Average yield**	**Total Scope**
**Ref.**	**Halo.**	**Alco.**	**Amine**	**Amide**	**Cyano**	**Acid**	**Ester**	**Aldehyde**	**Phenol**	**aniline**		**Score FG (%)**	**80.91**	**11**
**75**	1	0	0	0	0	0	0	0	0	0		10		
	**Electronic compatibility**							**Versatility**						
	**Styr.**	**Olefin**	**Difun.**	**Ter. Alky.**				**Rad.**	**Elect.**	**Nucleo.**				
	11/11	0/11						X	X					
	**Functional group tolerance**												**Average yield**	**Total Scope**
**Ref.**	**Halo.**	**Alco.**	**Amine**	**Amide**	**Cyano**	**Acid**	**Ester**	**Aldehyde**	**Phenol**	**aniline**		**Score FG (%)**	**87.91**	**11**
**115**	1	0	0	0	0	0	0	0	0	0		10		
	**Electronic compatibility**							**Versatility**						
	**Styr.**	**Olefin**	**Difun.**	**Ter. Alky.**				**Rad.**	**Elect.**	**Nucleo.**				
	11/11	0/11						X						

	**Functional group tolerance**												**Average yield**	**Total Scope**
**Ref.**	**Halo.**	**Alco.**	**Amine**	**Amide**	**Cyano**	**Acid**	**Ester**	**Aldehyde**	**Phenol**	**aniline**		**Score FG (%)**	**81.36**	**14**
**83**	1	0	0	1	1	0	0	0	0	0		30		
	**Electronic compatibility**							**Versatility**						
	**Styr.**	**Olefin**	**Difun.**	**Ter. Alky.**				**Rad.**	**Elect.**	**Nucleo.**				
	2/14	12/14	X	X				X						

Average scope: 19.7 or 2.0/10
Electronic compatibility: 4/5 or 8/10
Average yield: 79.7% or 8.0/10
Versatility: 5/6 or 8.3/10
Resource & health: Not toxic + price (0-50$/g) + Not recyclable = 4/5 or 8/10
Functional group tolerance: 1.8/10

Average score: 6.0/10

Tert-butyl nitrate (R13)

	Functional group tolerance												Average yield	Total Scope
**Ref.**	**Halo.**	**Alco.**	**Amine**	**Amide**	**Cyano**	**Acid**	**Ester**	**Aldehyde**	**Phenol**	**aniline**		**Score FG (%)**	**75.77**	**31**
**91**	1	0	0	1	1	0	0	0	0	0		30		
	**Electronic compatibility**							**Versatility**						
	**Styr.**	**Olefin**	**Difun.**	**Ter. Alky.**				**Rad.**	**Elect.**	**Nucleo.**				
	0/31	31/31	X	X				X						
	**Functional group tolerance**												**Average yield**	**Total Scope**
**Ref.**	**Halo.**	**Alco.**	**Amine**	**Amide**	**Cyano**	**Acid**	**Ester**	**Aldehyde**	**Phenol**	**aniline**		**Score FG (%)**	**76.93**	**16**
**92**	1	0	0	1	0	0	0	0	0	0		20		
	**Electronic compatibility**							**Versatility**						
	**Styr.**	**Olefin**	**Difun.**	**Ter. Alky.**				**Rad.**	**Elect.**	**Nucleo.**				
	1/16	15/16	X	x				x						
	**Functional group tolerance**												**Average yield**	**Total Scope**
**Ref.**	**Halo.**	**Alco.**	**Amine**	**Amide**	**Cyano**	**Acid**	**Ester**	**Aldehyde**	**Phenol**	**aniline**		**Score FG (%)**	**74.43**	**35**

**93**	1	0	0	0	1	0	1	1	0	0		40		
	**Electronic compatibility**							**Versatility**						
	**Styr.**	**Olefin**	**Difun.**	**Ter. Alky.**				**Rad.**	**Elect.**	**Nucleo.**				
	21/35	14/35		X				x						
	**Functional group tolerance**												**Average yield**	**Total Scope**
**Ref.**	**Halo.**	**Alco.**	**Amine**	**Amide**	**Cyano**	**Acid**	**Ester**	**Aldehyde**	**Phenol**	**aniline**		**Score FG (%)**	**62.96**	**24**
**94**	1	0	0	0	1	0	0	0	0	0		20		
	**Electronic compatibility**							**Versatility**						
	**Styr.**	**Olefin**	**Difun.**	**Ter. Alky.**				**Rad.**	**Elect.**	**Nucleo.**				
	23/24	1/24	X	X				X						
	**Functional group tolerance**												**Average yield**	**Total Scope**
**Ref.**	**Halo.**	**Alco.**	**Amine**	**Amide**	**Cyano**	**Acid**	**Ester**	**Aldehyde**	**Phenol**	**aniline**		**Score FG (%)**	**76.42**	**26**
**95**	1	0	0	0	0	0	1	0	0	0		20		
	**Electronic compatibility**							**Versatility**						
	**Styr.**	**Olefin**	**Difun.**	**Ter. Alky.**				**Rad.**	**Elect.**	**Nucleo.**				
	20/26	6/26	X	X				X						

	Functional group tolerance												Average yield	Total Scope
Ref.	Halo.	Alco.	Amine	Amide	Cyano	Acid	Ester	Aldehyde	Phenol	aniline		Score FG (%)	65.65	20
96	1	0	0	0	1	0	0	0	0	0		20		
	Electronic compatibility							Versatility						
	Styr.	Olefin	Difun.	Ter. Alky.				Rad.	Elect.	Nucleo.				
	20/20	0/20		X				X						
	Functional group tolerance												Average yield	Total Scope
Ref.	Halo.	Alco.	Amine	Amide	Cyano	Acid	Ester	Aldehyde	Phenol	aniline		Score FG (%)	62.19	16
97	1	0	0	0	1	0	1	0	0	0		30		
	Electronic compatibility							Versatility						
	Styr.	Olefin	Difun.	Ter. Alky.				Rad.	Elect.	Nucleo.				
	16/16	0/16	X	X				X						
	Functional group tolerance												Average yield	Total Scope
Ref.	Halo.	Alco.	Amine	Amide	Cyano	Acid	Ester	Aldehyde	Phenol	aniline		Score FG (%)	64.68	25
	1	0	0	0	1	0	1	1	0	0		40		

**98**	**Electronic compatibility**							**Versatility**						
	**Styr.**	**Olefin**	**Difun.**	**Ter. Alky.**				**Rad.**	**Elect.**	**Nucleo.**				
	22/25	3/25	X	X				X						
	**Functional group tolerance**												**Average yield**	**Total Scope**
**Ref.**	**Halo.**	**Alco.**	**Amine**	**Amide**	**Cyano**	**Acid**	**Ester**	**Aldehyde**	**Phenol**	**aniline**		**Score FG (%)**	**45.58**	**12**
**99**	0	0	0	0	1	0	1	1	0	0		30		
	**Electronic compatibility**							**Versatility**						
	**Styr.**	**Olefin**	**Difun.**	**Ter. Alky.**				**Rad.**	**Elect.**	**Nucleo.**				
	0/12	12/12	X	X				X						
	**Functional group tolerance**												**Average yield**	**Total Scope**
**Ref.**	**Halo.**	**Alco.**	**Amine**	**Amide**	**Cyano**	**Acid**	**Ester**	**Aldehyde**	**Phenol**	**aniline**		**Score FG (%)**	**42.07**	**19**
**100**	1	0	0	0	0	0	1	0	0	0		20		
	**Electronic compatibility**							**Versatility**						
	**Styr.**	**Olefin**	**Difun.**	**Ter. Alky.**				**Rad.**	**Elect.**	**Nucleo.**				
	8/19	11/19	X	X				X						

	**Functional group tolerance**												**Average yield**	**Total Scope**
**Ref.**	**Halo.**	**Alco.**	**Amine**	**Amide**	**Cyano**	**Acid**	**Ester**	**Aldehyde**	**Phenol**	**aniline**		**Score FG (%)**	**62.29**	**21**
	1	0	0	1	0	0	1	0	0	0		30		
**101**	**Electronic compatibility**							**Versatility**						
	**Styr.**	**Olefin**	**Difun.**	**Ter. Alky.**				**Rad.**	**Elect.**	**Nucleo.**				
	0/21	21/21	X	X				X						
	**Functional group tolerance**												**Average yield**	**Total Scope**
**Ref.**	**Halo.**	**Alco.**	**Amine**	**Amide**	**Cyano**	**Acid**	**Ester**	**Aldehyde**	**Phenol**	**aniline**		**Score FG (%)**	**81.0**	**13**
	1	0	0	1	0	0	0	0	0	0		20		
**102**	**Electronic compatibility**							**Versatility**						
	**Styr.**	**Olefin**	**Difun.**	**Ter. Alky.**				**Rad.**	**Elect.**	**Nucleo.**				
	0/13	13/13	X	X				X						
	**Functional group tolerance**												**Average yield**	**Total Scope**
**Ref.**	**Halo.**	**Alco.**	**Amine**	**Amide**	**Cyano**	**Acid**	**Ester**	**Aldehyde**	**Phenol**	**aniline**		**Score FG (%)**	**66.93**	**41**
	1	0	0	1	1	0	1	0	0	0		40		

103	Electronic compatibility							Versatility						
	Styr.	Olefin	Difun.	Ter. Alky.				Rad.	Elect.	Nucleo.				
	1/41	40/41	X	X				X						
	Functional group tolerance												Average yield	Total Scope
Ref.	Halo.	Alco.	Amine	Amide	Cyano	Acid	Ester	Aldehyde	Phenol	aniline		Score FG (%)	66.92	25
104	1	0	0	1	0	0	1	1	0	0		40		
	Electronic compatibility							Versatility						
	Styr.	Olefin	Difun.	Ter. Alky.				Rad.	Elect.	Nucleo.				
	1/25	24/25	X	X				X						
	Functional group tolerance												Average yield	Total Scope
Ref.	Halo.	Alco.	Amine	Amide	Cyano	Acid	Ester	Aldehyde	Phenol	aniline		Score FG (%)	66.39	23
105	1	0	0	1	0	0	0	0	0	0		20		
	Electronic compatibility							Versatility						
	Styr.	Olefin	Difun.	Ter. Alky.				Rad.	Elect.	Nucleo.				
	0/23	23/23	X	X				X						

	Functional group tolerance												Average yield	Total Scope
**Ref.**	**Halo.**	**Alco.**	**Amine**	**Amide**	**Cyano**	**Acid**	**Ester**	**Aldehyde**	**Phenol**	**aniline**		**Score FG (%)**	**72.86**	**12**
**106**	1	0	0	1	0	0	0	0	0	0		20		
	**Electronic compatibility**							**Versatility**						
	**Styr.**	**Olefin**	**Difun.**	**Ter. Alky.**				**Rad.**	**Elect.**	**Nucleo.**				
	1/12	11/12	X	X				X						

	Functional group tolerance												Average yield	Total Scope
**Ref.**	**Halo.**	**Alco.**	**Amine**	**Amide**	**Cyano**	**Acid**	**Ester**	**Aldehyde**	**Phenol**	**aniline**		**Score FG (%)**	**77.43**	**21**
**107**	1	0	0	1	0	0	1	0	0	0		30		
	**Electronic compatibility**							**Versatility**						
	**Styr.**	**Olefin**	**Difun.**	**Ter. Alky.**				**Rad.**	**Elect.**	**Nucleo.**				
	20/21	1/21	X	X				X						

Average scope: 22.4 or 2.2/10
Electronic compatibility: 4/5 or 8/10
Average yield: 67.1% or 6.7/10
Versatility: 2/6 or 3.3/10
Resource & health: Not toxic + price (0-50$/g) + Not recyclable = 4/5 or 8/10
Functional group tolerance: 2.8/10

Average score: 5.2/10

$NaNO_2$ (R3)

	Functional group tolerance												Average yield	Total Scope
**Ref.**	**Halo.**	**Alco.**	**Amine**	**Amide**	**Cyano**	**Acid**	**Ester**	**Aldehyde**	**Phenol**	**aniline**		**Score FG (%)**	**85.19**	**77**
**122**	1	0	0	0	1	0	1	1	0	0		40		
	**Electronic compatibility**							**Versatility**						
	**Styr.**	**Olefin**	**Difun.**	**Ter. Alky.**				**Rad.**	**Elect.**	**Nucleo.**				
	77/77	0/77	X	X				X						
	**Functional group tolerance**												**Average yield**	**Total Scope**
**Ref.**	**Halo.**	**Alco.**	**Amine**	**Amide**	**Cyano**	**Acid**	**Ester**	**Aldehyde**	**Phenol**	**aniline**		**Score FG (%)**	**67.86**	**36**
**125**	1	0	0	1	0	0	0	0	0	0		20		
	**Electronic compatibility**							**Versatility**						
	**Styr.**	**Olefin**	**Difun.**	**Ter. Alky.**				**Rad.**	**Elect.**	**Nucleo.**				
	0/36	36/36	X	X				X						
	**Functional group tolerance**												**Average yield**	**Total Scope**
**Ref.**	**Halo.**	**Alco.**	**Amine**	**Amide**	**Cyano**	**Acid**	**Ester**	**Aldehyde**	**Phenol**	**aniline**		**Score FG (%)**	**68.93**	**29**

	1	0	0	0	1	0	1	0	0	0		30		
**124**	**Electronic compatibility**							**Versatility**						
	**Styr.**	**Olefin**	**Difun.**	**Ter. Alky.**				**Rad.**	**Elect.**	**Nucleo.**				
	25/29	4/29		X				X						
	**Functional group tolerance**												**Average yield**	**Total Scope**
**Ref.**	**Halo.**	**Alco.**	**Amine**	**Amide**	**Cyano**	**Acid**	**Ester**	**Aldehyde**	**Phenol**	**aniline**		**Score FG (%)**	**59.55**	**20**
	1	0	0	0	0	0	1	0	1	0		30		
**67**	**Electronic compatibility**							**Versatility**						
	**Styr.**	**Olefin**	**Difun.**	**Ter. Alky.**				**Rad.**	**Elect.**	**Nucleo.**				
	16/20	4/20		X				X						
	**Functional group tolerance**												**Average yield**	**Total Scope**
**Ref.**	**Halo.**	**Alco.**	**Amine**	**Amide**	**Cyano**	**Acid**	**Ester**	**Aldehyde**	**Phenol**	**aniline**		**Score FG (%)**	**59.55**	**28**
	1	0	0	0	0	0	0	0	0	0		10		
**126**	**Electronic compatibility**							**Versatility**						
	**Styr.**	**Olefin**	**Difun.**	**Ter. Alky.**				**Rad.**	**Elect.**	**Nucleo.**				
	24/28	4/28		X				X						

	Functional group tolerance												Average yield	Total Scope
Ref.	Halo.	Alco.	Amine	Amide	Cyano	Acid	Ester	Aldehyde	Phenol	aniline		Score FG (%)	65.42	24
127	1	0	0	1	0	0	0	0	0	0		20		
	Electronic compatibility							Versatility						
	Styr.	Olefin	Difun.	Ter. Alky.				Rad.	Elect.	Nucleo.				
	1/24	23/24	X	X				X						

	Functional group tolerance												Average yield	Total Scope
Ref.	Halo.	Alco.	Amine	Amide	Cyano	Acid	Ester	Aldehyde	Phenol	aniline		Score FG (%)	78.88	25
128	1	0	0	0	0	0	1	1	0	0		30		
	Electronic compatibility							Versatility						
	Styr.	Olefin	Difun.	Ter. Alky.				Rad.	Elect.	Nucleo.				
	8/25	17/25		X				X						

	Functional group tolerance												Average yield	Total Scope
Ref.	Halo.	Alco.	Amine	Amide	Cyano	Acid	Ester	Aldehyde	Phenol	aniline		Score FG (%)	64.64	14
	1	0	0	0	0	0	0	0	0	0		30		

**85**	**Electronic compatibility**							**Versatility**						
	**Styr.**	**Olefin**	**Difun.**	**Ter. Alky.**				**Rad.**	**Elect.**	**Nucleo.**				
	14/14	0/14		X				X						
	**Functional group tolerance**												**Average yield**	**Total Scope**
**Ref.**	**Halo.**	**Alco.**	**Amine**	**Amide**	**Cyano**	**Acid**	**Ester**	**Aldehyde**	**Phenol**	**aniline**		**Score FG (%)**	**63.89**	**19**
**129**	1	0	0	0	0	0	0	1	0	0		20		
	**Electronic compatibility**							**Versatility**						
	**Styr.**	**Olefin**	**Difun.**	**Ter. Alky.**				**Rad.**	**Elect.**	**Nucleo.**				
	19/19	0/19	X	X				X						
	**Functional group tolerance**												**Average yield**	**Total Scope**
**Ref.**	**Halo.**	**Alco.**	**Amine**	**Amide**	**Cyano**	**Acid**	**Ester**	**Aldehyde**	**Phenol**	**aniline**		**Score FG (%)**	**58.64**	**21**
**130**	1	0	0	0	0	0	1	0	0	0		20		
	**Electronic compatibility**							**Versatility**						
	**Styr.**	**Olefin**	**Difun.**	**Ter. Alky.**				**Rad.**	**Elect.**	**Nucleo.**				
	8/21	13/21		X				X						

Average scope: 29.3 or 2.9/10
Electronic compatibility: 4/5 or 8/10
Average yield: 67.3% or 6.7/10
Versatility: 4/6 or 6.6/10
Resource & health: Toxic + price (50-200$/g) + Not recyclable = 1/5 or 2/10
Functional group tolerance: 2.8/10

Average score: 4.8/10

NO (R20)

	Functional group tolerance												Average yield	Total Scope
Ref.	Halo.	Alco.	Amine	Amide	Cyano	Acid	Ester	Aldehyde	Phenol	aniline		Score FG (%)	71.27	15
131	1	0	1	0	0	0	1	0	0	0		30		
	Electronic compatibility							Versatility						
	Styr.	Olefin	Difun.	Ter. Alky.				Rad.	Elect.	Nucleo.				
	8/15	7/15		X				x						

	Functional group tolerance												Average yield	Total Scope
Ref.	Halo.	Alco.	Amine	Amide	Cyano	Acid	Ester	Aldehyde	Phenol	aniline		Score FG (%)	44.88	8
132	1	1	0	0	0	0	1	0	0	0		30		
	Electronic compatibility							Versatility						
	Styr.	Olefin	Difun.	Ter. Alky.				Rad.	Elect.	Nucleo.				
	5/8	3/8	X	x				X						

Average scope: 11.5 or 1.2/10
Electronic compatibility: 4/5 or 8/10
Average yield: 58.1% or 5.8/10
Versatility: 2/6 or 3.3/10
Resource & health: Toxic + price (0-50$/g) + Not recyclable = 2/5 or 4/10
Functional group tolerance: 3/10
Average score: 4.2/10

$AgNO_3$ (R7)

	Functional group tolerance												Average yield	Total Scope
**Ref.**	**Halo.**	**Alco.**	**Amine**	**Amide**	**Cyano**	**Acid**	**Ester**	**Aldehyde**	**Phenol**	**aniline**		**Score FG (%)**	**58.21**	**19**
**116**	0	0	0	0	0	0	1	0	0	0		10		
	**Electronic compatibility**							**Versatility**						
	**Styr.**	**Olefin**	**Difun.**	**Ter. Alky.**				**Rad.**	**Elect.**	**Nucleo.**				
	4/19	15/19		x					x					

	Functional group tolerance												Average yield	Total Scope
**Ref.**	**Halo.**	**Alco.**	**Amine**	**Amide**	**Cyano**	**Acid**	**Ester**	**Aldehyde**	**Phenol**	**aniline**		**Score FG (%)**	**68.64**	**22**
**117**	1	0	0	1	0	0	1	0	0	0		30		
	**Electronic compatibility**							**Versatility**						
	**Styr.**	**Olefin**	**Difun.**	**Ter. Alky.**				**Rad.**	**Elect.**	**Nucleo.**				
	1/22	21/22	X	x				X						

Average scope: 20.5 or 2.1/10
Electronic compatibility: 5/5 or 10/10
Average yield: 63.4% or 6.3/10
Versatility: 3/6 or 5/10
Resource & health: Toxic + price (50-200$/g) + Not recyclable = 1/5 or 2/10
Functional group tolerance: 2/10
Average score: 4.6/10

HNO_3 (R8)

	Functional group tolerance												Average yield	Total Scope
**Ref.**	**Halo.**	**Alco.**	**Amine**	**Amide**	**Cyano**	**Acid**	**Ester**	**Aldehyde**	**Phenol**	**aniline**		**Score FG (%)**	**58.21**	**16**
**119**	1	0	0	0	0	0	1	0	0	0		20		
	**Electronic compatibility**							**Versatility**						
	**Styr.**	**Olefin**	**Difun.**	**Ter. Alky.**				**Rad.**	**Elect.**	**Nucleo.**				
	12/16	4/16	X	X					x					

	Functional group tolerance												Average yield	Total Scope
**Ref.**	**Halo.**	**Alco.**	**Amine**	**Amide**	**Cyano**	**Acid**	**Ester**	**Aldehyde**	**Phenol**	**aniline**		**Score FG (%)**	**60.0**	**7**
**86**	0	0	0	0	0	0	0	0	0	0		0		
	**Electronic compatibility**							**Versatility**						
	**Styr.**	**Olefin**	**Difun.**	**Ter. Alky.**				**Rad.**	**Elect.**	**Nucleo.**				
	7/7	0/7		x				X						

Average scope: 11.5 or 1.2/10
Electronic compatibility: 3/5 or 6/10
Average yield: 59.1% or 5.9/10
Versatility: 4/6 or 6.6/10
Resource & health: Toxic + price (50-200$/g) + Not recyclable = 1/5 or 2/10
Functional group tolerance: 1/10
Average score: 3.8/10

$AgNO_2$ (R18)

	Functional group tolerance												Average yield	Total Scope
Ref.	Halo.	Alco.	Amine	Amide	Cyano	Acid	Ester	Aldehyde	Phenol	aniline		Score FG (%)	78.93	27
87	1	1	0	1	1	1	1	0	0	0		60		
	Electronic compatibility							Versatility						
	Styr.	Olefin	Difun.	Ter. Alky.				Rad.	Elect.	Nucleo.				
	14/27	13/27		X				X						

	Functional group tolerance												Average yield	Total Scope
Ref.	Halo.	Alco.	Amine	Amide	Cyano	Acid	Ester	Aldehyde	Phenol	aniline		Score FG (%)	74.81	41
88	1	0	0	1	1	0	1	1	0	0		50		
	Electronic compatibility							Versatility						
	Styr.	Olefin	Difun.	Ter. Alky.				Rad.	Elect.	Nucleo.				
	23/41	18/41		x				X						

Average scope: 34 or 3.4/10
Electronic compatibility: 3/5 or 6/10
Average yield: 76.9% or 7.7/10
Versatility: 2/6 or 3.3/10
Resource & health: Not toxic + price (0-50$/g) + Not recyclable = 4/5 or 8/10
Functional group tolerance: 5.5/10
Average score: 5.7/10

$Mg(NO_3)_2.6H_2O$ (R19)

	**Functional group tolerance**												**Average yield**	**Total Scope**
**Ref.**	**Halo.**	**Alco.**	**Amine**	**Amide**	**Cyano**	**Acid**	**Ester**	**Aldehyde**	**Phenol**	**aniline**		**Score FG (%)**	**49.25**	**16**
	1	0	0	1	0	0	0	0	0	0		20		
**89**	**Electronic compatibility**							**Versatility**						
	**Styr.**	**Olefin**	**Difun.**	**Ter. Alky.**				**Rad.**	**Elect.**	**Nucleo.**				
	1/16	15/16	X	X				X						
	**Functional group tolerance**												**Average yield**	**Total Scope**
**Ref.**	**Halo.**	**Alco.**	**Amine**	**Amide**	**Cyano**	**Acid**	**Ester**	**Aldehyde**	**Phenol**	**aniline**		**Score FG (%)**	**66.0**	**24**
	1	0	0	1	0	0	0	0	0	0		20		
**90**	**Electronic compatibility**							**Versatility**						
	**Styr.**	**Olefin**	**Difun.**	**Ter. Alky.**				**Rad.**	**Elect.**	**Nucleo.**				
	1/24	23/24	X	x				X						

Average scope: 20 or 2/10
Electronic compatibility: 5/5 or 10/10
Average yield: 57.6% or 5.8/10
Versatility: 2/6 or 3.3/10
Resource & health: Not toxic + price (0-50$/g) + Not recyclable = 4/5 or 8/10
Functional group tolerance: 2/10
Average score: 5.2/10

Anomeric nitroamide (R21)

	Functional group tolerance												Average yield	Total Scope
**Ref.**	**Halo.**	**Alco.**	**Amine**	**Amide**	**Cyano**	**Acid**	**Ester**	**Aldehyde**	**Phenol**	**aniline**		**Score FG (%)**	**48.42**	**31**
**121**	1	1	1	1	0	0	1	0	0	0		50		
	**Electronic compatibility**							**Versatility**						
	**Styr.**	**Olefin**	**Difun.**	**Ter. Alky.**				**Rad.**	**Elect.**	**Nucleo.**				
	0/31	31/31	X					X						

Average scope: 31 or 3.1/10
Electronic compatibility: 4/5 or 8/10
Average yield: 48.4% or 4.8/10
Versatility: 2/6 or 3.3/10
Resource & health: Unknown + price (Not commercially available) + Not recyclable = 2/5 or 4/10
Functional group tolerance: 5/10

Average score: 4.7/10

Guanidine nitrate (R17)

	Functional group tolerance												Average yield	Total Scope
**Ref.**	**Halo.**	**Alco.**	**Amine**	**Amide**	**Cyano**	**Acid**	**Ester**	**Aldehyde**	**Phenol**	**aniline**		**Score FG (%)**	**76.26**	**69**
**69**	1	0	0	1	1	0	1	0	0	0		40		
	**Electronic compatibility**							**Versatility**						
	**Styr.**	**Olefin**	**Difun.**	**Ter. Alky.**				**Rad.**	**Elect.**	**Nucleo.**				
	63/69	6/69	X	X					x					

Average scope: 69 or 6.9/10
Electronic compatibility: 3/5 or 6/10
Average yield: 76.3% or 7.6/10
Versatility: 2/6 or 3.3/10
Resource & health: Not toxic + price (0-50$/g) + Not recyclable = 4/5 or 8/10
Functional group tolerance: 4/10

Average score: 6.0/10

4. Heteroatom Nitration

Electronic compatibility (Total = 6 points)

1. Different heteroatoms (3 points)
   - Amine = 1
   - Alcohol = 1
   - Others = 1
2. Types (3 points total)
   - Primary = 1
   - Secondary = 1
   - tertiary = 1

Versatility (Total = 6 points)

Versatility was evaluated based on the number of mechanistic pathways accessible to the method and the diversity of nitrating species detected.

- 1 mechanism → 1 point
- 2 mechanisms → 2 points
- ≥3 mechanisms → 3 points

Additionally, 1 point was awarded for each distinct nitrating species observed (e.g., Nitronium ion, nitryl radical, nitrite) up to a maximum of 3 points, giving a total possible versatility score of 6 points.

$Cu(NO_3)2.3H_2O$ (R15)

	Functional group tolerance											Average yield	Total Scope
**Ref.**	**Halo.**	**Alco.**	**Amine**	**Amide**	**Cyano**	**Acid**	**Ester**	**Aldehyde**	**Phenol**	**aniline**	**Score FG (%)**	60.50	6
**133**	0	0	0	1	0	0	1	0	0	0	20		
	**Electronic compatibility**							**Versatility**					
	**Prim.**	**Sec.**	**Tert.**	**Amine**	**Alcohol**	**other**		**Rad.**	**Elect.**	**Nucleo.**			
	x	x		x				x					
	**Functional group tolerance**											**Average yield**	**Total Scope**
**Ref.**	**Halo.**	**Alco.**	**Amine**	**Amide**	**Cyano**	**Acid**	**Ester**	**Aldehyde**	**Phenol**	**aniline**	**Score FG (%)**	78.78	9
**143**	0	0	0	0	0	0	0	0	0	0	0		
	**Electronic compatibility**							**Versatility**					
	**Prim.**	**Sec.**	**Tert.**	**Amine**	**Alcohol**	**other**		**Rad.**	**Elect.**	**Nucleo.**			
		x		x					x				

Average scope: 7.5 or 0.75/10
Electronic compatibility: 3/6 or 5/10
Average yield: 69.6% or 7.0/10
Versatility: 3/6 or 5/10
Resource & health: Not toxic + price (0-50$/g) + not recyclable = 4/5 or 8/10
Functional group tolerance: 1/10

Average score: 4.5/10

$Fe(NO_3)_3.9H_2O$ (R1)

	Functional group tolerance											Average yield	Total Scope
**Ref.**	**Halo.**	**Alco.**	**Amine**	**Amide**	**Cyano**	**Acid**	**Ester**	**Aldehyde**	**Phenol**	**aniline**	**Score FG (%)**	63.82	22
**147**	1	0	0	0	1	1	0	0	0	0	30		
	**Electronic compatibility**							**Versatility**					
	**Prim.**	**Sec.**	**Tert.**	**Amine**	**Alcohol**	**other**		**Rad.**	**Elect.**	**Nucleo.**			
		x		x				x					

Average scope: 22 or 2.2/10
Electronic compatibility: 2/6 or 3.3/10
Average yield: 63.8% or 6.4/10
Versatility: one nitrating species + used in one mechanism = 2/6 or 3.3/10
Resource & health: Not toxic + price (0-50$/g) + not recyclable = 4/5 or 8/10
Functional group tolerance: 3/10

Average score: 4.4/10

$LiNO_3$ (R22)

	Functional group tolerance											Average yield	Total Scope
**Ref.**	**Halo.**	**Alco.**	**Amine**	**Amide**	**Cyano**	**Acid**	**Ester**	**Aldehyde**	**Phenol**	**aniline**	**Score FG (%)**	71.64	11
**142**	0	0	0	1	0	0	1	0	0	0	20		
	**Electronic compatibility**							**Versatility**					
	**Prim.**	**Sec.**	**Tert.**	**Amine**	**Alcohol**	**other**		**Rad.**	**Elect.**	**Nucleo.**			
	x	x		x					x				

Average scope: 11 or 1.1/10
Electronic compatibility: 2/6 or 3.3/10
Average yield: 71.64% or 7.2/10
Versatility: 2/6 or 3.3/10
Resource & health: Not toxic + price (0-50$/g) + not recyclable = 4/5 or 8/10
Functional group tolerance: 2/10

Average score: 4.4/10

N_2O_5 (R23)

	Functional group tolerance											Average yield	Total Scope
**Ref.**	**Halo.**	**Alco.**	**Amine**	**Amide**	**Cyano**	**Acid**	**Ester**	**Aldehyde**	**Phenol**	**aniline**	**Score FG (%)**	93.83	12
**150**	0	0	0	0	0	0	0	0	0	0	0		
	**Electronic compatibility**							**Versatility**					
	**Prim.**	**Sec.**	**Tert.**	**Amine**	**Alcohol**	**other**		**Rad.**	**Elect.**	**Nucleo.**			
	x	x			x				x				

Average scope: 12 or 1.2/10
Electronic compatibility: 3/6 or 5/10
Average yield: 93.8% or 9.4/10
Versatility: 2/6 or 3.3/10
Resource & health: Unknown + price (0-50$/g) + not recyclable = 3/5 or 6/10
Functional group tolerance: 0/10

Average score: 4.2/10

HNO_3 (R8)

	Functional group tolerance											Average yield	Total Scope
**Ref.**	**Halo.**	**Alco.**	**Amine**	**Amide**	**Cyano**	**Acid**	**Ester**	**Aldehyde**	**Phenol**	**aniline**	**Score FG (%)**	59.64	5
**134**	0	0	0	0	0	0	0	0	0	0	0		
	**Electronic compatibility**							**Versatility**					
	**Prim.**	**Sec.**	**Tert.**	**Amine**	**Alcohol**	**other**		**Rad.**	**Elect.**	**Nucleo.**			
	x				x				x				

	Functional group tolerance											Average yield	Total Scope
**Ref.**	**Halo.**	**Alco.**	**Amine**	**Amide**	**Cyano**	**Acid**	**Ester**	**Aldehyde**	**Phenol**	**aniline**	**Score FG (%)**	83.71	7
**135**	0	1	1	1	0	0	0	0	0	0	30		
	**Electronic compatibility**							**Versatility**					
	**Prim.**	**Sec.**	**Tert.**	**Amine**	**Alcohol**	**other**		**Rad.**	**Elect.**	**Nucleo.**			
	x	x		x	x				x				

	Functional group tolerance											Average yield	Total Scope
**Ref.**	**Halo.**	**Alco.**	**Amine**	**Amide**	**Cyano**	**Acid**	**Ester**	**Aldehyde**	**Phenol**	**aniline**	**Score FG (%)**	76.56	16
**136**	0	0	1	0	0	0	0	0	0	0	10		
	**Electronic compatibility**							**Versatility**					
	**Prim.**	**Sec.**	**Tert.**	**Amine**	**Alcohol**	**other**		**Rad.**	**Elect.**	**Nucleo.**			

	x	x	x		x				x				
	Functional group tolerance											Average yield	Total Scope
Ref.	Halo.	Alco.	Amine	Amide	Cyano	Acid	Ester	Aldehyde	Phenol	aniline	Score FG (%)	58.23	10
133	0	0	0	1	0	0	1	0	0	0	20		
	Electronic compatibility							Versatility					
	Prim.	Sec.	Tert.	Amine	Alcohol	other		Rad.	Elect.	Nucleo.			
	x	x		x					x				
	Functional group tolerance											Average yield	Total Scope
Ref.	Halo.	Alco.	Amine	Amide	Cyano	Acid	Ester	Aldehyde	Phenol	aniline	Score FG (%)	74.50	4
137	0	0	0	0	0	0	0	0	0	0	0		
	Electronic compatibility							Versatility					
	Prim.	Sec.	Tert.	Amine	Alcohol	other		Rad.	Elect.	Nucleo.			
	x	x		x					x				
	Functional group tolerance											Average yield	Total Scope
Ref.	Halo.	Alco.	Amine	Amide	Cyano	Acid	Ester	Aldehyde	Phenol	aniline	Score FG (%)	86.50	8
138	1	0	0	0	0	0	0	0	0	0	10		
	Electronic compatibility							Versatility					

	**Prim.**	**Sec.**	**Tert.**	**Amine**	**Alcohol**	**other**		**Rad.**	**Elect.**	**Nucleo.**			
	x			x					x				
	**Functional group tolerance**											**Average yield**	**Total Scope**
**Ref.**	**Halo.**	**Alco.**	**Amine**	**Amide**	**Cyano**	**Acid**	**Ester**	**Aldehyde**	**Phenol**	**aniline**	**Score FG (%)**	80.33	3
**139**	0	0	0	0	0	0	0	0	0	0	0		
	**Electronic compatibility**							**Versatility**					
	**Prim.**	**Sec.**	**Tert.**	**Amine**	**Alcohol**	**other**		**Rad.**	**Elect.**	**Nucleo.**			
		x			x				x				
	**Functional group tolerance**											**Average yield**	**Total Scope**
**Ref.**	**Halo.**	**Alco.**	**Amine**	**Amide**	**Cyano**	**Acid**	**Ester**	**Aldehyde**	**Phenol**	**aniline**	**Score FG (%)**	94.14	7
**140**	0	0	0	0	0	0	0	0	0	0	0		
	**Electronic compatibility**							**Versatility**					
	**Prim.**	**Sec.**	**Tert.**	**Amine**	**Alcohol**	**other**		**Rad.**	**Elect.**	**Nucleo.**			
	x	x	x		x				x				
	**Functional group tolerance**											**Average yield**	**Total Scope**
**Ref.**	**Halo.**	**Alco.**	**Amine**	**Amide**	**Cyano**	**Acid**	**Ester**	**Aldehyde**	**Phenol**	**aniline**	**Score FG (%)**	70.0	2
	0	0	0	0	0	0	0	0	0	0	0		

141	Electronic compatibility							Versatility					
	Prim.	Sec.	Tert.	Amine	Alcohol	other		Rad.	Elect.	Nucleo.			
	x			x					x				

Average scope: 6.9 or 0.7/10
Electronic compatibility: 4/6 or 6.6/10
Average yield: 76% or 7.6/10
Versatility: 2/6 or 3.3/10
Resource & health: Toxic + price (50-200$/g) + not recyclable = 1/5 or 2/10
Functional group tolerance: 1/10

Average score: 3.6/10

$AgNO_3$ (R7)

	Functional group tolerance											Average yield	Total Scope
Ref.	Halo.	Alco.	Amine	Amide	Cyano	Acid	Ester	Aldehyde	Phenol	aniline	Score FG (%)	54.50	14
144	0	0	0	0	0	0	0	0	0	0	0		
	Electronic compatibility							Versatility					
	Prim.	Sec.	Tert.	Amine	Alcohol	other		Rad.	Elect.	Nucleo.			
	x	x			x				x				

Average scope: 14 or 1.4/10
Electronic compatibility: 3/6 or 5/10
Average yield: 54.50% or 5.5/10
Versatility: 2/6 or 3.3/10
Resource & health: Toxic + price (50-200$/g) + not recyclable = 1/5 or 2/10
Functional group tolerance: 0/10

Average score: 2.9/10

***N*,6-dinitrosaccharin (R24)**

	Functional group tolerance											Average yield	Total Scope
**Ref.**	**Halo.**	**Alco.**	**Amine**	**Amide**	**Cyano**	**Acid**	**Ester**	**Aldehyde**	**Phenol**	**aniline**	**Score FG (%)**	67.74	40
**145**	1	1	0	1	0	0	1	0	0	0	40		
	**Electronic compatibility**							**Versatility**					
	**Prim.**	**Sec.**	**Tert.**	**Amine**	**Alcohol**	**other**		**Rad.**	**Elect.**	**Nucleo.**			
	x	x	x		x				x				

	Functional group tolerance											Average yield	Total Scope
**Ref.**	**Halo.**	**Alco.**	**Amine**	**Amide**	**Cyano**	**Acid**	**Ester**	**Aldehyde**	**Phenol**	**aniline**	**Score FG (%)**	68.81	26
**146**	1	0	0	1	0	0	0	0	0	0	20		
	**Electronic compatibility**							**Versatility**					
	**Prim.**	**Sec.**	**Tert.**	**Amine**	**Alcohol**	**other**		**Rad.**	**Elect.**	**Nucleo.**			
	x	x			x				x				

Average scope: 33 or 3.3/10
Electronic compatibility: 4/6 or 6.6/10
Average yield: 68.3% or 6.8/10
Versatility: 2/6 or 3.3/10
Resource & health: Not toxic + price (>200$/g) + Recyclable = 3/5 or 6/10
Functional group tolerance: 3/10

Average score: 5/10

***Tert*-butyl nitrite (R13)**

	Functional group tolerance											Average yield	Total Scope
Ref.	Halo.	Alco.	Amine	Amide	Cyano	Acid	Ester	Aldehyde	Phenol	aniline	Score FG (%)	66.46	26
148	1	0	0	1	1	0	1	0	0	0	40		
	Electronic compatibility							Versatility					
	Prim.	Sec.	Tert.	Amine	Alcohol	other		Rad.	Elect.	Nucleo.			
	x			x					x				

	Functional group tolerance											Average yield	Total Scope
Ref.	Halo.	Alco.	Amine	Amide	Cyano	Acid	Ester	Aldehyde	Phenol	aniline	Score FG (%)	56.0	26
149	0	0	0	0	0	0	0	0	0	0	0		
	Electronic compatibility							Versatility					
	Prim.	Sec.	Tert.	Amine	Alcohol	other		Rad.	Elect.	Nucleo.			
	x					x			x				

Average scope: 26 or 2.6/10
Electronic compatibility: 3/6 or 5/10
Average yield: 61.2% or 6.1/10
Versatility: 3/6 or 5/10
Resource & health: Not toxic + price (0-50$/g) + not recyclable = 4/5 or 8/10
Functional group tolerance: 2/10

Average score: 4.8/10

5. Aromatic Nitration

Electronic compatibility (Total = 5 points)

1. Heteroaromatics (1 point)
    - Presence of at least one nitrated heteroaromatic substrate → 1 point, otherwise 0.
2. Electronic Classes (4 points total)
   For each electronic class (electron-rich, electron-poor, electron-neutral), points were assigned based on the proportion of substrates represented in the scope:
    - ≥25% → 2 points
    - 15–25% → 1 point
    - 0–15% → 0 points

   Electronic class definitions:
   - Electron-rich: anilines, OMe, ≥2 halogens, alcohols, *t*Bu
   - Electron-poor: carbonyl, cyano, boronic acid, OCF_3, CF_3
   - Electron-neutral: 1 halogen, alkyl, unsubstituted benzene, alkyne-substituted aromatics

Versatility (Total = 6 points)

Versatility was evaluated based on the number of mechanistic pathways accessible to the method and the diversity of nitrating species detected.

- 1–2 mechanisms → 1 point
- 3–4 mechanisms → 2 points
- ≥4 mechanisms → 3 points

Additionally, 1 point was awarded for each distinct nitrating species observed (e.g., Nitronium ion, nitryl radical, nitrite) up to a maximum of 3 points, giving a total possible versatility score of 6 points.

$Fe(NO_3)_3.9H_2O$ (R1)

	Functional group tolerance												Average yield	Total Scope
Ref.	Halo.	Alco.	Amine	Amide	Cyano	Acid	Ester	Aldehyde	Phenol	aniline	Hetero.	Score FG (%)	63.48	22
156	1	0	0	0	0	0	0	0	0	0	1	18		
	Electronic compatibility							Versatility						
	Rich	Poor	Neut.					Rad.	Elect.	Nucleo.				
	38.1	4.8	57.1					x						
	Functional group tolerance												Average yield	Total Scope
Ref.	Halo.	Alco.	Amine	Amide	Cyano	Acid	Ester	Aldehyde	Phenol	aniline	Hetero.	Score FG (%)	64.56	25
215	1	0	1	1	1	0	1	0	0	0	0	45		
	Electronic compatibility							Versatility						
	Rich	Poor	Neut.					Rad.	Elect.	Nucleo.				
	28.0	16.0	56.0					x						
	Functional group tolerance												Average yield	Total Scope
Ref.	Halo.	Alco.	Amine	Amide	Cyano	Acid	Ester	Aldehyde	Phenol	aniline	Hetero.	Score FG (%)	45.31	32

	1	0	0	0	0	0	0	0	0	0	1	18		
**216**	**Electronic compatibility**							**Versatility**						
	**Rich**	**Poor**	**Neut.**					**Rad.**	**Elect.**	**Nucleo.**				
	75.0	0.0	25.0					x						
	**Functional group tolerance**												**Average yield**	**Total Scope**
**Ref.**	**Halo.**	**Alco.**	**Amine**	**Amide**	**Cyano**	**Acid**	**Ester**	**Aldehyde**	**Phenol**	**aniline**	**Hetero.**	**Score FG (%)**	52.05	19
	1	0	0	1	0	0	1	0	0	0	0	27		
**157**	**Electronic compatibility**							**Versatility**						
	**Rich**	**Poor**	**Neut.**					**Rad.**	**Elect.**	**Nucleo.**				
	15.8	31.6	52.6						x					
	**Functional group tolerance**												**Average yield**	**Total Scope**
**Ref.**	**Halo.**	**Alco.**	**Amine**	**Amide**	**Cyano**	**Acid**	**Ester**	**Aldehyde**	**Phenol**	**aniline**	**Hetero.**	**Score FG (%)**	53.97	20
	1	0	0	0	1	1	1	1	0	0	0	45		
**152**	**Electronic compatibility**							**Versatility**						
	**Rich**	**Poor**	**Neut.**					**Rad.**	**Elect.**	**Nucleo.**				
	45.0	45.0	10.0					x						

	Functional group tolerance												Average yield	Total Scope
**Ref.**	**Halo.**	**Alco.**	**Amine**	**Amide**	**Cyano**	**Acid**	**Ester**	**Aldehyde**	**Phenol**	**aniline**	**Hetero.**	**Score FG (%)**	68.82	17
**218**	1	0	0	1	1	0	1	0	0	0	1	45		
	**Electronic compatibility**							**Versatility**						
	**Rich**	**Poor**	**Neut.**					**Rad.**	**Elect.**	**Nucleo.**				
	0	0	100.0					x						
	Functional group tolerance												Average yield	Total Scope
**Ref.**	**Halo.**	**Alco.**	**Amine**	**Amide**	**Cyano**	**Acid**	**Ester**	**Aldehyde**	**Phenol**	**aniline**	**Hetero.**	**Score FG (%)**	68.82	17
	1	1	0	1	1	1	1	0	1	1	1	82		
**177**	**Electronic compatibility**							**Versatility**						
	**Rich**	**Poor**	**Neut.**					**Rad.**	**Elect.**	**Nucleo.**				
	30.3	40.4	29.3					x						
	Functional group tolerance												Average yield	Total Scope
**Ref.**	**Halo.**	**Alco.**	**Amine**	**Amide**	**Cyano**	**Acid**	**Ester**	**Aldehyde**	**Phenol**	**aniline**	**Hetero.**	**Score FG (%)**	65.13	24
	1	0	0	0	1	0	0	0	0	0	1	27		

**219**	**Electronic compatibility**							**Versatility**						
	**Rich**	**Poor**	**Neut.**					**Rad.**	**Elect.**	**Nucleo.**				
	0	0	100.0					x						
	**Functional group tolerance**												**Average yield**	**Total Scope**
**Ref.**	**Halo.**	**Alco.**	**Amine**	**Amide**	**Cyano**	**Acid**	**Ester**	**Aldehyde**	**Phenol**	**aniline**	**Hetero.**	**Score FG (%)**	81.89	56
**181**	1	1	1	0	1	1	1	1	1	1	0	82		
	**Electronic compatibility**							**Versatility**						
	**Rich**	**Poor**	**Neut.**					**Rad.**	**Elect.**	**Nucleo.**				
	69.6	14.3	16.1						x					
	**Functional group tolerance**												**Average yield**	**Total Scope**
**Ref.**	**Halo.**	**Alco.**	**Amine**	**Amide**	**Cyano**	**Acid**	**Ester**	**Aldehyde**	**Phenol**	**aniline**	**Hetero.**	**Score FG (%)**	64.95	37
**153**	1	1	0	0	0	0	1	0	1	0	1	45		
	**Electronic compatibility**							**Versatility**						
	**Rich**	**Poor**	**Neut.**					**Rad.**	**Elect.**	**Nucleo.**				
	89.2	0.0	10.8					x						

	Functional group tolerance												Average yield	Total Scope
**Ref.**	**Halo.**	**Alco.**	**Amine**	**Amide**	**Cyano**	**Acid**	**Ester**	**Aldehyde**	**Phenol**	**aniline**	**Hetero.**	**Score FG (%)**	72.19	24
**151**	1	0	0	0	0	0	0	1	0	1	0	27		
	**Electronic compatibility**							**Versatility**						
	**Rich**	**Poor**	**Neut.**					**Rad.**	**Elect.**	**Nucleo.**				
	100.0	0	0					x						
	**Functional group tolerance**												**Average yield**	**Total Scope**
**Ref.**	**Halo.**	**Alco.**	**Amine**	**Amide**	**Cyano**	**Acid**	**Ester**	**Aldehyde**	**Phenol**	**aniline**	**Hetero.**	**Score FG (%)**	80.00	14
**158**	1	0	0	0	0	0	0	0	0	1	0	18		
	**Electronic compatibility**							**Versatility**						
	**Rich**	**Poor**	**Neut.**					**Rad.**	**Elect.**	**Nucleo.**				
	64.3	0.0	35.7						x					
	**Functional group tolerance**												**Average yield**	**Total Scope**
**Ref.**	**Halo.**	**Alco.**	**Amine**	**Amide**	**Cyano**	**Acid**	**Ester**	**Aldehyde**	**Phenol**	**aniline**	**Hetero.**	**Score FG (%)**	80.12	42
	1	0	0	0	1	1	1	1	1	1	1	73		

37	Electronic compatibility							Versatility						
	Rich	Poor	Neut.					Rad.	Elect.	Nucleo.				
	26.2	14.3	59.5						x					

Average scope: 33 or 3.3/10
Electronic compatibility: 3/5 or 6/10
Average yield: 64.4% or 6.4/10
Versatility: 5/6 or 8.3/10
Resource & health: Not toxic + price (0-50$/g) + not recyclable = 4/5 or 8/10
Functional group tolerance: 4.1/10

Average score: 6.0/10

***Tetra*-butyl ammonium nitrate (R11)**

	**Functional group tolerance**												**Average yield**	**Total Scope**
**Ref.**	**Halo.**	**Alco.**	**Amine**	**Amide**	**Cyano**	**Acid**	**Ester**	**Aldehyde**	**Phenol**	**aniline**	**Hetero.**	**Score FG (%)**	**84.53**	**30**
**155**	1	0	0	0	1	1	1	1	1	1	1	73		
	**Electronic compatibility**							**Versatility**						
	**Rich**	**Poor**	**Neut.**					**Rad.**	**Elect.**	**Nucleo.**				
	26.7	36.7	36.7						x					
	**Functional group tolerance**												**Average yield**	**Total Scope**
**Ref.**	**Halo.**	**Alco.**	**Amine**	**Amide**	**Cyano**	**Acid**	**Ester**	**Aldehyde**	**Phenol**	**aniline**	**Hetero.**	**Score FG (%)**	84.31	16
**182**	1	0	0	0	0	1	0	1	1	0	0	36		
	**Electronic compatibility**							**Versatility**						
	**Rich**	**Poor**	**Neut.**					**Rad.**	**Elect.**	**Nucleo.**				
	18.8	12.5	68.8						x					
	**Functional group tolerance**												**Average yield**	**Total Scope**
**Ref.**	**Halo.**	**Alco.**	**Amine**	**Amide**	**Cyano**	**Acid**	**Ester**	**Aldehyde**	**Phenol**	**aniline**	**Hetero.**	**Score FG (%)**	80.30	23

	1	0	0	0	0	0	0	0	0	0	1	18		
**159**	**Electronic compatibility**							**Versatility**						
	**Rich**	**Poor**	**Neut.**					**Rad.**	**Elect.**	**Nucleo.**				
	0	0	100.0						x					

Average scope: 23 or 2.3/10
Electronic compatibility: 3/5 or 6/10
Average yield: 83% or 8.3/10
Versatility: 2/6 or 3.3/10
Resource & health: Not toxic + price (0-50$/g) + not recyclable = 4/5 or 8/10
Functional group tolerance: 4.2/10

Average score: 5.4/10

Tetra-butyl ammonium nitrite (R10)

	Functional group tolerance												Average yield	Total Scope
**Ref.**	**Halo.**	**Alco.**	**Amine**	**Amide**	**Cyano**	**Acid**	**Ester**	**Aldehyde**	**Phenol**	**aniline**	**Hetero.**	**Score FG (%)**	**48.25**	**20**
**214**	1	0	0	1	0	0	1	0	1	1	0	45		
	**Electronic compatibility**							**Versatility**						
	**Rich**	**Poor**	**Neut.**					**Rad.**	**Elect.**	**Nucleo.**				
	100.0	0	0					x						

Average scope: 20 or 2.0/10
Electronic compatibility: 2/5 or 4/10
Average yield: 48.3% or 4.8/10
Versatility: 2/6 or 3.3/10
Resource & health: Not toxic + price (0-50$/g) + not recyclable = 4/5 or 8/10
Functional group tolerance: 4.6/10

Average score: 4.5/10

***6,N*-dinitrosaccharin (R24)**

	Functional group tolerance												Average yield	Total Scope
**Ref.**	**Halo.**	**Alco.**	**Amine**	**Amide**	**Cyano**	**Acid**	**Ester**	**Aldehyde**	**Phenol**	**aniline**	**Hetero.**	**Score FG (%)**	67.48	27
**146**	1	0	0	0	0	0	1	1	0	0	0	27		
	**Electronic compatibility**							**Versatility**						
	**Rich**	**Poor**	**Neut.**					**Rad.**	**Elect.**	**Nucleo.**				
	63.0	7.4	29.6						x					

Average scope: 27 or 2.7/10
Electronic compatibility: 2/5 or 4/10
Average yield: 67.5% or 6.8/10
Versatility: 2/6 or 3.3/10
Resource & health: Not toxic + price (>200$/g) + Recyclable = 3/5 or 6/10
Functional group tolerance: 2/10

Average score: 4.3/10

***N*-nitrosaccharin (R9)**

	Functional group tolerance												Average yield	Total Scope
**Ref.**	**Halo.**	**Alco.**	**Amine**	**Amide**	**Cyano**	**Acid**	**Ester**	**Aldehyde**	**Phenol**	**aniline**	**Hetero.**	**Score FG (%)**	**85.75**	**65**
**183**	1	0	0	1	1	1	1	1	1	1	1	82		
	**Electronic compatibility**							**Versatility**						
	**Rich**	**Poor**	**Neut.**					**Rad.**	**Elect.**	**Nucleo.**				
	32.3	29.2	38.5						x					

Average scope: 65 or 6.5/10
Electronic compatibility: 5/5 or 10/10
Average yield: 85.7% or 8.6/10
Versatility: 2/6 or 3.3/10
Resource & health: Not toxic + price (50-200$/g) + Recyclable = 4/5 or 8/10
Functional group tolerance: 8.2/10

Average score: 7.4/10

$Cu(NO_3)_2.3H_2O$ (R15)

	Functional group tolerance												Average yield	Total Scope
Ref.	Halo.	Alco.	Amine	Amide	Cyano	Acid	Ester	Aldehyde	Phenol	aniline	Hetero.	Score FG (%)	**57.78**	**32**
160	1	0	0	1	0	0	1	0	0	0	0	27		
	Electronic compatibility							Versatility						
	Rich	Poor	Neut.					Rad.	Elect.	Nucleo.				
	9.4	6.3	84.4					x						
	Functional group tolerance												Average yield	Total Scope
Ref.	Halo.	Alco.	Amine	Amide	Cyano	Acid	Ester	Aldehyde	Phenol	aniline	Hetero.	Score FG (%)	92.20	5
161	0	0	0	0	0	0	0	0	0	0	0	0		
	Electronic compatibility							Versatility						
	Rich	Poor	Neut.					Rad.	Elect.	Nucleo.				
	60.0	0	40.0						x					
	Functional group tolerance												Average yield	Total Scope
Ref.	Halo.	Alco.	Amine	Amide	Cyano	Acid	Ester	Aldehyde	Phenol	aniline	Hetero.	Score FG (%)	76.93	15

248	1	0	0	0	0	0	0	0	0	0	0	9		
	Electronic compatibility							Versatility						
	Rich	Poor	Neut.					Rad.	Elect.	Nucleo.				
	53.3	0.0	46.7					x						
	Functional group tolerance												Average yield	Total Scope
Ref.	Halo.	Alco.	Amine	Amide	Cyano	Acid	Ester	Aldehyde	Phenol	aniline	Hetero.	Score FG (%)	65.64	32
249	1	0	0	1	0	0	0	0	0	0	0	18		
	Electronic compatibility							Versatility						
	Rich	Poor	Neut.					Rad.	Elect.	Nucleo.				
	37.5	0.0	62.5					x						
	Functional group tolerance												Average yield	Total Scope
Ref.	Halo.	Alco.	Amine	Amide	Cyano	Acid	Ester	Aldehyde	Phenol	aniline	Hetero.	Score FG (%)	62.39	18
162	1	0	0	0	0	0	0	0	0	0	0	9		
	Electronic compatibility							Versatility						
	Rich	Poor	Neut.					Rad.	Elect.	Nucleo.				
	61.1	0.0	38.9					x						

	Functional group tolerance												Average yield	Total Scope
Ref.	Halo.	Alco.	Amine	Amide	Cyano	Acid	Ester	Aldehyde	Phenol	aniline	Hetero.	Score FG (%)	72.33	18
218	1	0	0	1	1	0	1	0	0	0	1	45		
	Electronic compatibility							Versatility						
	Rich	Poor	Neut.					Rad.	Elect.	Nucleo.				
	0	0	100.0					x						

Average scope: 20 or 2.0/10
Electronic compatibility: 2/5 or 4/10
Average yield: 71.2% or 7.1/10
Versatility: 4/6 or 6.6/10
Resource & health: Not toxic + price (0-50$/g) + not recyclable = 4/5 or 8/10
Functional group tolerance: 1.8/10

Average score: 4.9/10

Tert-butyl nitrite (R13)

	Functional group tolerance												Average yield	Total Scope
Ref.	Halo.	Alco.	Amine	Amide	Cyano	Acid	Ester	Aldehyde	Phenol	aniline	Hetero.	Score FG (%)	72.40	5
	0	0	0	0	0	0	0	0	1	0	0	9		
220	Electronic compatibility							Versatility						
	Rich	Poor	Neut.					Rad.	Elect.	Nucleo.				
	100.0	0	0					x						

	Functional group tolerance												Average yield	Total Scope
Ref.	Halo.	Alco.	Amine	Amide	Cyano	Acid	Ester	Aldehyde	Phenol	aniline	Hetero.	Score FG (%)	48.50	16
	1	0	0	0	1	0	1	0	0	1	0	36		
221	Electronic compatibility							Versatility						
	Rich	Poor	Neut.					Rad.	Elect.	Nucleo.				
	37.5	0.0	62.5					x						

	Functional group tolerance												Average yield	Total Scope
Ref.	Halo.	Alco.	Amine	Amide	Cyano	Acid	Ester	Aldehyde	Phenol	aniline	Hetero.	Score FG (%)	74.28	27

222	1	0	0	0	0	0	0	0	0	0	0	9		
	**Electronic compatibility**							**Versatility**						
	**Rich**	**Poor**	**Neut.**					**Rad.**	**Elect.**	**Nucleo.**				
	51.9	18.5	29.6					x						
	**Functional group tolerance**												**Average yield**	**Total Scope**
**Ref.**	**Halo.**	**Alco.**	**Amine**	**Amide**	**Cyano**	**Acid**	**Ester**	**Aldehyde**	**Phenol**	**aniline**	**Hetero.**	**Score FG (%)**	63.20	20
223	1	0	0	1	0	0	0	0	0	0	1	27		
	**Electronic compatibility**							**Versatility**						
	**Rich**	**Poor**	**Neut.**					**Rad.**	**Elect.**	**Nucleo.**				
	15.0	5.0	80.0					x						
	**Functional group tolerance**												**Average yield**	**Total Scope**
**Ref.**	**Halo.**	**Alco.**	**Amine**	**Amide**	**Cyano**	**Acid**	**Ester**	**Aldehyde**	**Phenol**	**aniline**	**Hetero.**	**Score FG (%)**	63.85	69
224	1	0	0	0	1	0	1	0	0	0	0	27		
	**Electronic compatibility**							**Versatility**						
	**Rich**	**Poor**	**Neut.**					**Rad.**	**Elect.**	**Nucleo.**				
	14.5	8.7	76.8					x						

	Functional group tolerance												Average yield	Total Scope
Ref.	Halo.	Alco.	Amine	Amide	Cyano	Acid	Ester	Aldehyde	Phenol	aniline	Hetero.	Score FG (%)	64.45	11
225	1	0	0	0	0	0	0	0	0	0	1	18		
	Electronic compatibility							Versatility						
	Rich	Poor	Neut.					Rad.	Elect.	Nucleo.				
	27.3	0.0	72.7					x						

	Functional group tolerance												Average yield	Total Scope
Ref.	Halo.	Alco.	Amine	Amide	Cyano	Acid	Ester	Aldehyde	Phenol	aniline	Hetero.	Score FG (%)	42.88	64
226	1	0	0	1	1	0	1	0	0	0	1	45		
	Electronic compatibility							Versatility						
	Rich	Poor	Neut.					Rad.	Elect.	Nucleo.				
	0	0	100.0					x						

	Functional group tolerance												Average yield	Total Scope
Ref.	Halo.	Alco.	Amine	Amide	Cyano	Acid	Ester	Aldehyde	Phenol	aniline	Hetero.	Score FG (%)	77.42	64
	1	0	0	1	0	0	1	0	0	0	0	27		

**227**	**Electronic compatibility**							**Versatility**					
	**Rich**	**Poor**	**Neut.**					**Rad.**	**Elect.**	**Nucleo.**			
	12.1	0.0	87.9					x					
	**Functional group tolerance**											**Average yield**	**Total Scope**
**Ref.**	**Halo.**	**Alco.**	**Amine**	**Amide**	**Cyano**	**Acid**	**Ester**	**Aldehyde**	**Phenol**	**aniline**	**Hetero.**	**Score FG (%)**	43.87	14
**228**	1	0	0	1	0	0	0	0	0	0	0	18		
	**Electronic compatibility**							**Versatility**					
	**Rich**	**Poor**	**Neut.**					**Rad.**	**Elect.**	**Nucleo.**			
	0	0	100.0					x					
	**Functional group tolerance**											**Average yield**	**Total Scope**
**Ref.**	**Halo.**	**Alco.**	**Amine**	**Amide**	**Cyano**	**Acid**	**Ester**	**Aldehyde**	**Phenol**	**aniline**	**Hetero.**	**Score FG (%)**	59.87	14
**163**	1	0	0	0	0	0	0	0	1	0	0	18		
	**Electronic compatibility**							**Versatility**					
	**Rich**	**Poor**	**Neut.**					**Rad.**	**Elect.**	**Nucleo.**			
	100.0	0	0					x					

	Functional group tolerance												Average yield	Total Scope
**Ref.**	**Halo.**	**Alco.**	**Amine**	**Amide**	**Cyano**	**Acid**	**Ester**	**Aldehyde**	**Phenol**	**aniline**	**Hetero.**	**Score FG (%)**	47.54	13
	1	0	0	0	0	0	0	0	0	0	0	9		
**229**	**Electronic compatibility**							**Versatility**						
	**Rich**	**Poor**	**Neut.**					**Rad.**	**Elect.**	**Nucleo.**				
	23.1	15.4	61.5					x						
	**Functional group tolerance**												**Average yield**	**Total Scope**
**Ref.**	**Halo.**	**Alco.**	**Amine**	**Amide**	**Cyano**	**Acid**	**Ester**	**Aldehyde**	**Phenol**	**aniline**	**Hetero.**	**Score FG (%)**	76.29	24
	1	0	0	0	0	0	1	0	0	0	0	18		
**230**	**Electronic compatibility**							**Versatility**						
	**Rich**	**Poor**	**Neut.**					**Rad.**	**Elect.**	**Nucleo.**				
	29.2	16.7	54.2					x						
	**Functional group tolerance**												**Average yield**	**Total Scope**
**Ref.**	**Halo.**	**Alco.**	**Amine**	**Amide**	**Cyano**	**Acid**	**Ester**	**Aldehyde**	**Phenol**	**aniline**	**Hetero.**	**Score FG (%)**	70.96	23
	1	0	0	1	1	0	1	0	0	0	0	36		

**231**	**Electronic compatibility**							**Versatility**						
	**Rich**	**Poor**	**Neut.**					**Rad.**	**Elect.**	**Nucleo.**				
	0	0	100.0					x						
	**Functional group tolerance**												**Average yield**	**Total Scope**
**Ref.**	**Halo.**	**Alco.**	**Amine**	**Amide**	**Cyano**	**Acid**	**Ester**	**Aldehyde**	**Phenol**	**aniline**	**Hetero.**	**Score FG (%)**	57.59	30
**250**	1	0	0	0	0	0	1	0	0	0	1	27		
	**Electronic compatibility**							**Versatility**						
	**Rich**	**Poor**	**Neut.**					**Rad.**	**Elect.**	**Nucleo.**				
	0	0	100.0					x						
	**Functional group tolerance**												**Average yield**	**Total Scope**
**Ref.**	**Halo.**	**Alco.**	**Amine**	**Amide**	**Cyano**	**Acid**	**Ester**	**Aldehyde**	**Phenol**	**aniline**	**Hetero.**	**Score FG (%)**	68.81	32
**233**	1	0	0	0	1	0	0	1	0	0	1	36		
	**Electronic compatibility**							**Versatility**						
	**Rich**	**Poor**	**Neut.**					**Rad.**	**Elect.**	**Nucleo.**				
	0	0	100.0					x						

	Functional group tolerance												Average yield	Total Scope
**Ref.**	**Halo.**	**Alco.**	**Amine**	**Amide**	**Cyano**	**Acid**	**Ester**	**Aldehyde**	**Phenol**	**aniline**	**Hetero.**	**Score FG (%)**	67.95	38
**234**	1	0	0	0	0	0	0	0	0	1	0	18		
	**Electronic compatibility**							**Versatility**						
	**Rich**	**Poor**	**Neut.**					**Rad.**	**Elect.**	**Nucleo.**				
	44.7	0.0	55.3					x						

	Functional group tolerance												Average yield	Total Scope
**Ref.**	**Halo.**	**Alco.**	**Amine**	**Amide**	**Cyano**	**Acid**	**Ester**	**Aldehyde**	**Phenol**	**aniline**	**Hetero.**	**Score FG (%)**	75.39	18
**235**	1	0	0	1	0	0	0	0	0	0	0	18		
	**Electronic compatibility**							**Versatility**						
	**Rich**	**Poor**	**Neut.**					**Rad.**	**Elect.**	**Nucleo.**				
	22.2	22.2	55.6					x						

	Functional group tolerance												Average yield	Total Scope
**Ref.**	**Halo.**	**Alco.**	**Amine**	**Amide**	**Cyano**	**Acid**	**Ester**	**Aldehyde**	**Phenol**	**aniline**	**Hetero.**	**Score FG (%)**	81.00	6
	1	0	0	0	0	0	1	1	0	1	0	36		

**236**	**Electronic compatibility**							**Versatility**						
	**Rich**	**Poor**	**Neut.**					**Rad.**	**Elect.**	**Nucleo.**				
	18.8	37.5	43.8					x						
	**Functional group tolerance**												**Average yield**	**Total Scope**
**Ref.**	**Halo.**	**Alco.**	**Amine**	**Amide**	**Cyano**	**Acid**	**Ester**	**Aldehyde**	**Phenol**	**aniline**	**Hetero.**	**Score FG (%)**	58.89	18
**237**	1	0	0	1	0	0	1	0	0	0	0	27		
	**Electronic compatibility**							**Versatility**						
	**Rich**	**Poor**	**Neut.**					**Rad.**	**Elect.**	**Nucleo.**				
	38.9	16.7	44.4					x						
	**Functional group tolerance**												**Average yield**	**Total Scope**
**Ref.**	**Halo.**	**Alco.**	**Amine**	**Amide**	**Cyano**	**Acid**	**Ester**	**Aldehyde**	**Phenol**	**aniline**	**Hetero.**	**Score FG (%)**	68.80	24
**238**	1	0	0	1	0	0	0	0	0	0	0	18		
	**Electronic compatibility**							**Versatility**						
	**Rich**	**Poor**	**Neut.**					**Rad.**	**Elect.**	**Nucleo.**				
	20.8	0.0	79.2					x						

	Functional group tolerance												Average yield	Total Scope
**Ref.**	**Halo.**	**Alco.**	**Amine**	**Amide**	**Cyano**	**Acid**	**Ester**	**Aldehyde**	**Phenol**	**aniline**	**Hetero.**	**Score FG (%)**	75.97	33
	1	0	0	0	0	0	1	0	0	0	0	18		
**251**	**Electronic compatibility**							**Versatility**						
	**Rich**	**Poor**	**Neut.**					**Rad.**	**Elect.**	**Nucleo.**				
	3.0	0	97.0					x						
	**Functional group tolerance**												**Average yield**	**Total Scope**
**Ref.**	**Halo.**	**Alco.**	**Amine**	**Amide**	**Cyano**	**Acid**	**Ester**	**Aldehyde**	**Phenol**	**aniline**	**Hetero.**	**Score FG (%)**	59.93	29
	1	1	0	1	1	0	1	0	0	0	0	45		
**232**	**Electronic compatibility**							**Versatility**						
	**Rich**	**Poor**	**Neut.**					**Rad.**	**Elect.**	**Nucleo.**				
	27.6	0.0	72.4					x						
	**Functional group tolerance**												**Average yield**	**Total Scope**
**Ref.**	**Halo.**	**Alco.**	**Amine**	**Amide**	**Cyano**	**Acid**	**Ester**	**Aldehyde**	**Phenol**	**aniline**	**Hetero.**	**Score FG (%)**	60.74	60
	1	1	0	1	1	0	1	1	0	1	1	73		

239	Electronic compatibility							Versatility						
	Rich	Poor	Neut.					Rad.	Elect.	Nucleo.				
	31.7	13.3	55.0					x						
	Functional group tolerance												Average yield	Total Scope
Ref.	Halo.	Alco.	Amine	Amide	Cyano	Acid	Ester	Aldehyde	Phenol	aniline	Hetero.	Score FG (%)	71.17	24
238	1	0	0	0	1	0	1	0	0	0	0	27		
	Electronic compatibility							Versatility						
	Rich	Poor	Neut.					Rad.	Elect.	Nucleo.				
	100.0	0	0					x						

Average scope: 27.3 or 2.7/10
Electronic compatibility: 2/5 or 4/10
Average yield: 64.2% or 6.4/10
Versatility: 3/6 or 5/10
Resource & health: Not toxic + price (0-50$/g) + not recyclable = 4/5 or 8/10
Functional group tolerance: 2.7/10

Average score: 4.8/10

$NaNO_2$ (R3)

	Functional group tolerance												Average yield	Total Scope
**Ref.**	**Halo.**	**Alco.**	**Amine**	**Amide**	**Cyano**	**Acid**	**Ester**	**Aldehyde**	**Phenol**	**aniline**	**Hetero.**	**Score FG (%)**	**71.18**	**17**
**164**	1	0	0	1	0	0	0	0	0	0	0	18		
	**Electronic compatibility**							**Versatility**						
	**Rich**	**Poor**	**Neut.**					**Rad.**	**Elect.**	**Nucleo.**				
	0	0	100.0					x						

	Functional group tolerance												Average yield	Total Scope
**Ref.**	**Halo.**	**Alco.**	**Amine**	**Amide**	**Cyano**	**Acid**	**Ester**	**Aldehyde**	**Phenol**	**aniline**	**Hetero.**	**Score FG (%)**	65.68	31
**247**	1	0	0	1	0	0	1	0	0	0	0	27		
	**Electronic compatibility**							**Versatility**						
	**Rich**	**Poor**	**Neut.**					**Rad.**	**Elect.**	**Nucleo.**				
	48.4	6.5	45.2					x						

	Functional group tolerance												Average yield	Total Scope
**Ref.**	**Halo.**	**Alco.**	**Amine**	**Amide**	**Cyano**	**Acid**	**Ester**	**Aldehyde**	**Phenol**	**aniline**	**Hetero.**	**Score FG (%)**	65.68	31

**154**	1	0	0	1	0	1	0	0	0	1	1	45		
	**Electronic compatibility**							**Versatility**						
	**Rich**	**Poor**	**Neut.**					**Rad.**	**Elect.**	**Nucleo.**				
	78.3	0.0	21.7					x						
	**Functional group tolerance**												**Average yield**	**Total Scope**
**Ref.**	**Halo.**	**Alco.**	**Amine**	**Amide**	**Cyano**	**Acid**	**Ester**	**Aldehyde**	**Phenol**	**aniline**	**Hetero.**	**Score FG (%)**	74.10	20
**253**	1	0	0	1	0	0	0	0	1	1	0	36		
	**Electronic compatibility**							**Versatility**						
	**Rich**	**Poor**	**Neut.**					**Rad.**	**Elect.**	**Nucleo.**				
	55.0	5.0	40.0					x						
	**Functional group tolerance**												**Average yield**	**Total Scope**
**Ref.**	**Halo.**	**Alco.**	**Amine**	**Amide**	**Cyano**	**Acid**	**Ester**	**Aldehyde**	**Phenol**	**aniline**	**Hetero.**	**Score FG (%)**	69.50	24
**184**	1	0	0	0	0	1	0	0	1	0	1	36		
	**Electronic compatibility**							**Versatility**						
	**Rich**	**Poor**	**Neut.**					**Rad.**	**Elect.**	**Nucleo.**				
	50.0	8.3	41.7						x					

	Functional group tolerance											Average yield	Total Scope
Ref.	Halo.	Alco.	Amine	Amide	Cyano	Acid	Ester	Aldehyde	Phenol	aniline	Hetero.	Score FG (%)	61.41	22
235	1	0	0	1	0	0	0	0	0	0	0	18		
	Electronic compatibility							Versatility						
	Rich	Poor	Neut.					Rad.	Elect.	Nucleo.				
	13.6	40.9	45.5						x					

	Functional group tolerance												Average yield	Total Scope
Ref.	Halo.	Alco.	Amine	Amide	Cyano	Acid	Ester	Aldehyde	Phenol	aniline	Hetero.	Score FG (%)	53.55	20
21	1	0	0	0	1	0	1	0	0	0	0	27		
	Electronic compatibility							Versatility						
	Rich	Poor	Neut.					Rad.	Elect.	Nucleo.				
	60.0	40.0	0							x				

	Functional group tolerance												Average yield	Total Scope
Ref.	Halo.	Alco.	Amine	Amide	Cyano	Acid	Ester	Aldehyde	Phenol	aniline	Hetero.	Score FG (%)	60.34	38
	1	0	0	0	1	0	1	0	0	0	0	27		

**254**	**Electronic compatibility**							**Versatility**						
	**Rich**	**Poor**	**Neut.**					**Rad.**	**Elect.**	**Nucleo.**				
	42.1	10.5	47.4					x						
	**Functional group tolerance**												**Average yield**	**Total Scope**
**Ref.**	**Halo.**	**Alco.**	**Amine**	**Amide**	**Cyano**	**Acid**	**Ester**	**Aldehyde**	**Phenol**	**aniline**	**Hetero.**	**Score FG (%)**	70.43	30
	1	0	0	1	0	0	0	0	0	1	0	27		
**252**	**Electronic compatibility**							**Versatility**						
	**Rich**	**Poor**	**Neut.**					**Rad.**	**Elect.**	**Nucleo.**				
	66.7	3.3	33.3					x						
	**Functional group tolerance**												**Average yield**	**Total Scope**
**Ref.**	**Halo.**	**Alco.**	**Amine**	**Amide**	**Cyano**	**Acid**	**Ester**	**Aldehyde**	**Phenol**	**aniline**	**Hetero.**	**Score FG (%)**	79.67	38
	1	0	0	1	1	0	1	1	0	1	1	64		
**240**	**Electronic compatibility**							**Versatility**						
	**Rich**	**Poor**	**Neut.**					**Rad.**	**Elect.**	**Nucleo.**				
	44.7	10.5	44.7							x				

Average scope: 26.3 or 2.6/10
Electronic compatibility: 2/5 or 4/10
Average yield: 65.6% or 6.6/10
Versatility: 5/6 or 8.3/10
Resource & health: Toxic + price (50-200$/g) + not recyclable = 1/5 or 2/10
Functional group tolerance: 3.3/10

Average score: 4.5/10

$NaNO_3$ (R31)

	Functional group tolerance												Average yield	Total Scope
Ref.	Halo.	Alco.	Amine	Amide	Cyano	Acid	Ester	Aldehyde	Phenol	aniline	Hetero.	Score FG (%)	84.29	14
185	1	0	0	0	0	0	0	1	1	1	0	36		
	Electronic compatibility							Versatility						
	Rich	Poor	Neut.					Rad.	Elect.	Nucleo.				
	35.7	7.1	57.1						x					

	Functional group tolerance												Average yield	Total Scope
Ref.	Halo.	Alco.	Amine	Amide	Cyano	Acid	Ester	Aldehyde	Phenol	aniline	Hetero.	Score FG (%)	94.07	14
186	1	0	0	0	0	0	0	1	1	1	0	36		
	Electronic compatibility							Versatility						
	Rich	Poor	Neut.					Rad.	Elect.	Nucleo.				
	35.7	7.1	57.1						x					

	Functional group tolerance												Average yield	Total Scope
Ref.	Halo.	Alco.	Amine	Amide	Cyano	Acid	Ester	Aldehyde	Phenol	aniline	Hetero.	Score FG (%)	70.86	13

**165**	1	0	0	0	0	0	0	0	1	0	1	27		
	**Electronic compatibility**							**Versatility**						
	**Rich**	**Poor**	**Neut.**					**Rad.**	**Elect.**	**Nucleo.**				
	15.4	7.7	76.9						x					
	**Functional group tolerance**												**Average yield**	**Total Scope**
**Ref.**	**Halo.**	**Alco.**	**Amine**	**Amide**	**Cyano**	**Acid**	**Ester**	**Aldehyde**	**Phenol**	**aniline**	**Hetero.**	**Score FG (%)**	85.81	16
**187**	1	0	0	0	0	0	0	1	1	1	0	36		
	**Electronic compatibility**							**Versatility**						
	**Rich**	**Poor**	**Neut.**					**Rad.**	**Elect.**	**Nucleo.**				
	62.5	0.0	37.5						x					
	**Functional group tolerance**												**Average yield**	**Total Scope**
**Ref.**	**Halo.**	**Alco.**	**Amine**	**Amide**	**Cyano**	**Acid**	**Ester**	**Aldehyde**	**Phenol**	**aniline**	**Hetero.**	**Score FG (%)**	92.55	11
**166**	1	0	0	1	0	1	0	0	1	1	0	45		
	**Electronic compatibility**							**Versatility**						
	**Rich**	**Poor**	**Neut.**					**Rad.**	**Elect.**	**Nucleo.**				
	18.2	27.3	54.5						x					

Average scope: 13.6 or 1.4/10
Electronic compatibility: 2/5 or 4/10
Average yield: 85.5% or 8.6/10
Versatility: 2/6 or 3.3/10
Resource & health: Not toxic + price (0-50$/g) + not recyclable = 4/5 or 8/10
Functional group tolerance: 3.6/10

Average score: 4.8/10

KNO_3 (R29)

	Functional group tolerance												Average yield	Total Scope
**Ref.**	**Halo.**	**Alco.**	**Amine**	**Amide**	**Cyano**	**Acid**	**Ester**	**Aldehyde**	**Phenol**	**aniline**	**Hetero.**	**Score FG (%)**	**87.29**	**7**
**188**	1	0	0	0	0	0	0	0	0	0	0	9		
	**Electronic compatibility**							**Versatility**						
	**Rich**	**Poor**	**Neut.**					**Rad.**	**Elect.**	**Nucleo.**				
	57.1	0	42.9						x					
	Functional group tolerance												Average yield	Total Scope
**Ref.**	**Halo.**	**Alco.**	**Amine**	**Amide**	**Cyano**	**Acid**	**Ester**	**Aldehyde**	**Phenol**	**aniline**	**Hetero.**	**Score FG (%)**	79.56	70
**180**	1	0	0	0	1	1	1	0	1	0	1	55		
	**Electronic compatibility**							**Versatility**						
	**Rich**	**Poor**	**Neut.**					**Rad.**	**Elect.**	**Nucleo.**				
	18.6	54.3	27.1						x					

Average scope: 38.5 or 3.8/10
Electronic compatibility: 5/5 or 10/10
Average yield: 83.4% or 8.3/10
Versatility: 2/6 or 3.3/10
Resource & health: Not toxic + price (0-50$/g) + not recyclable = 4/5 or 8/10
Functional group tolerance: 3.2/10
Average score: 6.1/10

$AgNO_3$ (R7)

	Functional group tolerance												Average yield	Total Scope
**Ref.**	**Halo.**	**Alco.**	**Amine**	**Amide**	**Cyano**	**Acid**	**Ester**	**Aldehyde**	**Phenol**	**aniline**	**Hetero.**	**Score FG (%)**	**52.70**	**13**
**167**	1	0	0	0	0	0	0	0	1	1	0	27		
	**Electronic compatibility**							**Versatility**						
	**Rich**	**Poor**	**Neut.**					**Rad.**	**Elect.**	**Nucleo.**				
	100.0	0	0						x					

	Functional group tolerance												Average yield	Total Scope
**Ref.**	**Halo.**	**Alco.**	**Amine**	**Amide**	**Cyano**	**Acid**	**Ester**	**Aldehyde**	**Phenol**	**aniline**	**Hetero.**	**Score FG (%)**	63.55	15
**168**	1	0	0	0	1	0	0	0	1	1	0	36		
	**Electronic compatibility**							**Versatility**						
	**Rich**	**Poor**	**Neut.**					**Rad.**	**Elect.**	**Nucleo.**				
	60.0	0	40.0						x					

	Functional group tolerance												Average yield	Total Scope
**Ref.**	**Halo.**	**Alco.**	**Amine**	**Amide**	**Cyano**	**Acid**	**Ester**	**Aldehyde**	**Phenol**	**aniline**	**Hetero.**	**Score FG (%)**	58.17	24

	1	0	0	0	0	0	1	0	0	0	0	18		
**255**	**Electronic compatibility**							**Versatility**						
	**Rich**	**Poor**	**Neut.**					**Rad.**	**Elect.**	**Nucleo.**				
	25.0	4.2	70.8					x						
	**Functional group tolerance**												**Average yield**	**Total Scope**
**Ref.**	**Halo.**	**Alco.**	**Amine**	**Amide**	**Cyano**	**Acid**	**Ester**	**Aldehyde**	**Phenol**	**aniline**	**Hetero.**	**Score FG (%)**	74.07	15
	1	0	0	1	0	0	0	0	0	0	0	18		
**256**	**Electronic compatibility**							**Versatility**						
	**Rich**	**Poor**	**Neut.**					**Rad.**	**Elect.**	**Nucleo.**				
	13.3	20.0	66.7					x						
	**Functional group tolerance**												**Average yield**	**Total Scope**
**Ref.**	**Halo.**	**Alco.**	**Amine**	**Amide**	**Cyano**	**Acid**	**Ester**	**Aldehyde**	**Phenol**	**aniline**	**Hetero.**	**Score FG (%)**	51.06	18
	1	0	0	0	0	0	1	0	0	0	0	18		
**257**	**Electronic compatibility**							**Versatility**						
	**Rich**	**Poor**	**Neut.**					**Rad.**	**Elect.**	**Nucleo.**				
	44.4	22.2	33.3					x						

Average scope: 17 or 1.7/10
Electronic compatibility: 2/5 or 4/10
Average yield: 59.9% or 6.0/10
Versatility: 3/6 or 5/10
Resource & health: Toxic + price (0-50$/g) + not recyclable = 2/5 or 4/10
Functional group tolerance: 2.4/10

Average score: 3.8/10

HNO_3 (R8)

	Functional group tolerance												Average yield	Total Scope
**Ref.**	**Halo.**	**Alco.**	**Amine**	**Amide**	**Cyano**	**Acid**	**Ester**	**Aldehyde**	**Phenol**	**aniline**	**Hetero.**	**Score FG (%)**	**81.78**	**21**
**258**	1	0	0	0	1	0	0	0	0	0	0	18		
	**Electronic compatibility**							**Versatility**						
	**Rich**	**Poor**	**Neut.**					**Rad.**	**Elect.**	**Nucleo.**				
	9.5	33.3	57.1					x						
	**Functional group tolerance**												**Average yield**	**Total Scope**
**Ref.**	**Halo.**	**Alco.**	**Amine**	**Amide**	**Cyano**	**Acid**	**Ester**	**Aldehyde**	**Phenol**	**aniline**	**Hetero.**	**Score FG (%)**	73.78	9
**189**	1	0	0	0	0	0	0	0	0	0	0	9		
	**Electronic compatibility**							**Versatility**						
	**Rich**	**Poor**	**Neut.**					**Rad.**	**Elect.**	**Nucleo.**				
	22.2	11.1	66.7						x					
	**Functional group tolerance**												**Average yield**	**Total Scope**
**Ref.**	**Halo.**	**Alco.**	**Amine**	**Amide**	**Cyano**	**Acid**	**Ester**	**Aldehyde**	**Phenol**	**aniline**	**Hetero.**	**Score FG (%)**	81.17	6

190	1	0	0	0	0	0	0	0	0	1	0	18		
	Electronic compatibility							Versatility						
	Rich	Poor	Neut.					Rad.	Elect.	Nucleo.				
	33.3	0.0	66.7						x					
	Functional group tolerance												Average yield	Total Scope
Ref.	Halo.	Alco.	Amine	Amide	Cyano	Acid	Ester	Aldehyde	Phenol	aniline	Hetero.	Score FG (%)	69.40	5
191	1	0	0	0	0	0	0	0	0	0	0	9		
	Electronic compatibility							Versatility						
	Rich	Poor	Neut.					Rad.	Elect.	Nucleo.				
	20.0	0	80.0						x					
	Functional group tolerance												Average yield	Total Scope
Ref.	Halo.	Alco.	Amine	Amide	Cyano	Acid	Ester	Aldehyde	Phenol	aniline	Hetero.	Score FG (%)	81.18	11
192	1	0	0	0	0	0	0	0	0	0	0	9		
	Electronic compatibility							Versatility						
	Rich	Poor	Neut.					Rad.	Elect.	Nucleo.				
	36.4	0	63.6						x					

	Functional group tolerance												Average yield	Total Scope
**Ref.**	**Halo.**	**Alco.**	**Amine**	**Amide**	**Cyano**	**Acid**	**Ester**	**Aldehyde**	**Phenol**	**aniline**	**Hetero.**	**Score FG (%)**	91.62	13
**193**	1	0	1	0	0	0	0	0	0	1	0	27		
	**Electronic compatibility**							**Versatility**						
	**Rich**	**Poor**	**Neut.**					**Rad.**	**Elect.**	**Nucleo.**				
	76.9	0.0	23.1						x					
	Functional group tolerance												Average yield	Total Scope
**Ref.**	**Halo.**	**Alco.**	**Amine**	**Amide**	**Cyano**	**Acid**	**Ester**	**Aldehyde**	**Phenol**	**aniline**	**Hetero.**	**Score FG (%)**	76.48	21
**194**	1	0	1	0	0	0	0	1	1	1	1	55		
	**Electronic compatibility**							**Versatility**						
	**Rich**	**Poor**	**Neut.**					**Rad.**	**Elect.**	**Nucleo.**				
	38.1	9.5	52.4						x					
	Functional group tolerance												Average yield	Total Scope
**Ref.**	**Halo.**	**Alco.**	**Amine**	**Amide**	**Cyano**	**Acid**	**Ester**	**Aldehyde**	**Phenol**	**aniline**	**Hetero.**	**Score FG (%)**	74.89	20
	1	0	0	1	0	0	0	1	1	1	1	55		

Ref.														
195	Electronic compatibility							Versatility						
	Rich	Poor	Neut.					Rad.	Elect.	Nucleo.				
	40.0	10.0	50.0						x					
	Functional group tolerance												Average yield	Total Scope
Ref.	Halo.	Alco.	Amine	Amide	Cyano	Acid	Ester	Aldehyde	Phenol	aniline	Hetero.	Score FG (%)	73.75	12
196	1	0	0	1	0	0	0	1	1	1	1	55		
	Electronic compatibility							Versatility						
	Rich	Poor	Neut.					Rad.	Elect.	Nucleo.				
	25.0	8.3	66.7						x					
	Functional group tolerance												Average yield	Total Scope
Ref.	Halo.	Alco.	Amine	Amide	Cyano	Acid	Ester	Aldehyde	Phenol	aniline	Hetero.	Score FG (%)	75.67	49
197	1	0	0	1	1	1	1	0	0	1	0	55		
	Electronic compatibility							Versatility						
	Rich	Poor	Neut.					Rad.	Elect.	Nucleo.				
	22.4	0	77.6					x						

	Functional group tolerance												Average yield	Total Scope
Ref.	Halo.	Alco.	Amine	Amide	Cyano	Acid	Ester	Aldehyde	Phenol	aniline	Hetero.	Score FG (%)	90.48	4
198	0	0	0	0	0	0	0	0	0	0	0	0		
	Electronic compatibility							Versatility						
	Rich	Poor	Neut.					Rad.	Elect.	Nucleo.				
	75.0	0	25.0						x					

	Functional group tolerance												Average yield	Total Scope
Ref.	Halo.	Alco.	Amine	Amide	Cyano	Acid	Ester	Aldehyde	Phenol	aniline	Hetero.	Score FG (%)	67.63	27
199	1	0	0	0	0	0	0	0	0	0	0	9		
	Electronic compatibility							Versatility						
	Rich	Poor	Neut.					Rad.	Elect.	Nucleo.				
	7.4	7.4	85.2						x					

	Functional group tolerance												Average yield	Total Scope
Ref.	Halo.	Alco.	Amine	Amide	Cyano	Acid	Ester	Aldehyde	Phenol	aniline	Hetero.	Score FG (%)	70.60	10
	0	0	0	0	0	0	0	0	0	0	0	0		

200	Electronic compatibility							Versatility						
	Rich	Poor	Neut.					Rad.	Elect.	Nucleo.				
	60.0	0	40.0						x					
	Functional group tolerance												Average yield	Total Scope
Ref.	Halo.	Alco.	Amine	Amide	Cyano	Acid	Ester	Aldehyde	Phenol	aniline	Hetero.	Score FG (%)	55.28	29
201	0	0	0	1	0	0	1	0	1	1	0	36		
	Electronic compatibility							Versatility						
	Rich	Poor	Neut.					Rad.	Elect.	Nucleo.				
	20.7	17.2	62.1						x					
	Functional group tolerance												Average yield	Total Scope
Ref.	Halo.	Alco.	Amine	Amide	Cyano	Acid	Ester	Aldehyde	Phenol	aniline	Hetero.	Score FG (%)	72.25	13
202	1	0	0	0	0	0	1	0	0	0	1	27		
	Electronic compatibility							Versatility						
	Rich	Poor	Neut.					Rad.	Elect.	Nucleo.				
	30.8	0	69.2						x					

	Functional group tolerance												Average yield	Total Scope
**Ref.**	**Halo.**	**Alco.**	**Amine**	**Amide**	**Cyano**	**Acid**	**Ester**	**Aldehyde**	**Phenol**	**aniline**	**Hetero.**	**Score FG (%)**	95.84	19
**203**	1	0	0	0	1	0	1	0	0	0	0	27		
	**Electronic compatibility**							**Versatility**						
	**Rich**	**Poor**	**Neut.**					**Rad.**	**Elect.**	**Nucleo.**				
	31.6	26.3	42.1						x					

Average scope: 16.8 or 1.7/10
Electronic compatibility: 2/5 or 4/10
Average yield: 77% or 7.7/10
Versatility: 3/6 or 5/10
Resource & health: Toxic + price (50-200$/g) + not recyclable = 1/5 or 2/10
Functional group tolerance: 2.6/10

Average score: 3.8/10

N_2O_4 (R43)

	Functional group tolerance												Average yield	Total Scope
**Ref.**	**Halo.**	**Alco.**	**Amine**	**Amide**	**Cyano**	**Acid**	**Ester**	**Aldehyde**	**Phenol**	**aniline**	**Hetero.**	**Score FG (%)**	**85.67**	**6**
**259**	1	0	0	0	0	0	0	0	0	0	0	9		
	**Electronic compatibility**							**Versatility**						
	**Rich**	**Poor**	**Neut.**					**Rad.**	**Elect.**	**Nucleo.**				
	0	0	100					x						

Average scope: 6 or 0.6/10
Electronic compatibility: 1/5 or 2/10
Average yield: 85.7% or 8.6/10
Versatility: 2/6 or 3.3/10
Resource & health: toxic + price (50-200$/g) + not recyclable = 1/5 or 2/10
Functional group tolerance: 0.9/10

Average score: 2.9/10

$AgNO_2$ (R18)

	Functional group tolerance												Average yield	Total Scope
**Ref.**	**Halo.**	**Alco.**	**Amine**	**Amide**	**Cyano**	**Acid**	**Ester**	**Aldehyde**	**Phenol**	**aniline**	**Hetero.**	**Score FG (%)**	**78.77**	**31**
**169**	1	0	0	0	0	0	0	0	0	1	1	27		
	**Electronic compatibility**							**Versatility**						
	**Rich**	**Poor**	**Neut.**					**Rad.**	**Elect.**	**Nucleo.**				
	41.9	9.7	48.4							x				

Average scope: 31 or 3.1/10
Electronic compatibility: 3/5 or 6/10
Average yield: 78.8% or 7.9/10
Versatility: 2/6 or 3.3/10
Resource & health: Not toxic + price (0-50$/g) + not recyclable = 4/5 or 8/10
Functional group tolerance: 2.7/10

Average score: 5.2/10

Urea Nitrate (R28)

	Functional group tolerance												Average yield	Total Scope
**Ref.**	**Halo.**	**Alco.**	**Amine**	**Amide**	**Cyano**	**Acid**	**Ester**	**Aldehyde**	**Phenol**	**aniline**	**Hetero.**	**Score FG (%)**	**85.40**	**5**
**170**	0	0	0	0	0	0	0	1	0	0	0	9		
	**Electronic compatibility**							**Versatility**						
	**Rich**	**Poor**	**Neut.**					**Rad.**	**Elect.**	**Nucleo.**				
	100	0	0						x					

	Functional group tolerance												Average yield	Total Scope
**Ref.**	**Halo.**	**Alco.**	**Amine**	**Amide**	**Cyano**	**Acid**	**Ester**	**Aldehyde**	**Phenol**	**aniline**	**Hetero.**	**Score FG (%)**	94.14	7
**204**	0	0	0	0	0	1	0	0	0	1	0	18		
	**Electronic compatibility**							**Versatility**						
	**Rich**	**Poor**	**Neut.**					**Rad.**	**Elect.**	**Nucleo.**				
	0	0	100						x					

Average scope: 6 or 0.6/10
Electronic compatibility: 2/5 or 4/10
Average yield: 89.8% or 9.0/10
Versatility: 2/6 or 3.3/10
Resource & health: Not toxic + price (0-50$/g) + not recyclable = 4/5 or 8/10
Functional group tolerance: 1.4/10
Average score: 4.4/10

Guanidine nitrate (R17)

	Functional group tolerance												Average yield	Total Scope
**Ref.**	**Halo.**	**Alco.**	**Amine**	**Amide**	**Cyano**	**Acid**	**Ester**	**Aldehyde**	**Phenol**	**aniline**	**Hetero.**	**Score FG (%)**	**72.59**	**27**
**205**	1	0	0	1	0	0	1	0	1	1	0	45		
	**Electronic compatibility**							**Versatility**						
	**Rich**	**Poor**	**Neut.**					**Rad.**	**Elect.**	**Nucleo.**				
	25.9	11.1	63.0						x					

	Functional group tolerance												Average yield	Total Scope
**Ref.**	**Halo.**	**Alco.**	**Amine**	**Amide**	**Cyano**	**Acid**	**Ester**	**Aldehyde**	**Phenol**	**aniline**	**Hetero.**	**Score FG (%)**	68.73	29
**171**	0	1	0	1	0	0	1	0	1	1	0	45		
	**Electronic compatibility**							**Versatility**						
	**Rich**	**Poor**	**Neut.**					**Rad.**	**Elect.**	**Nucleo.**				
	89.7	0	10.3						x					

Average scope: 28 or 2.8/10
Electronic compatibility: 2/5 or 4/10
Average yield: 70.7% or 7.1/10
Versatility: 2/6 or 3.3/10
Resource & health: Not toxic + price (0-50$/g) + not recyclable = 4/5 or 8/10
Functional group tolerance: 4.6/10
Average score: 5.0/10

Al(NO$_3$)$_3$.9H$_2$O (R16)

	Functional group tolerance												Average yield	Total Scope
**Ref.**	**Halo.**	**Alco.**	**Amine**	**Amide**	**Cyano**	**Acid**	**Ester**	**Aldehyde**	**Phenol**	**aniline**	**Hetero.**	**Score FG (%)**	**34.33**	**5**
**172**	1	0	0	0	0	0	0	0	0	0	0	9		
	**Electronic compatibility**							**Versatility**						
	**Rich**	**Poor**	**Neut.**					**Rad.**	**Elect.**	**Nucleo.**				
	40	20	40						x					

	Functional group tolerance												Average yield	Total Scope
**Ref.**	**Halo.**	**Alco.**	**Amine**	**Amide**	**Cyano**	**Acid**	**Ester**	**Aldehyde**	**Phenol**	**aniline**	**Hetero.**	**Score FG (%)**	58.73	50
**173**	1	0	1	1	1	0	0	1	1	1	1	73		
	**Electronic compatibility**							**Versatility**						
	**Rich**	**Poor**	**Neut.**					**Rad.**	**Elect.**	**Nucleo.**				
	80	0	20						x					

Average scope: 27.5 or 2.8/10
Electronic compatibility: 3/5 or 6/10
Average yield: 46.5% or 4.7/10
Versatility: 2/6 or 3.3/10
Resource & health: Toxic + price (0-50$/g) + not recyclable = 2/5 or 4/10
Functional group tolerance: 4.1/10
Average score: 4.1/10

$LiNO_3$ (R22)

	Functional group tolerance												Average yield	Total Scope
**Ref.**	**Halo.**	**Alco.**	**Amine**	**Amide**	**Cyano**	**Acid**	**Ester**	**Aldehyde**	**Phenol**	**aniline**	**Hetero.**	**Score FG (%)**	**32.84**	**8**
**172**	0	1	0	0	0	0	0	1	0	0	1	27		
	**Electronic compatibility**							**Versatility**						
	**Rich**	**Poor**	**Neut.**					**Rad.**	**Elect.**	**Nucleo.**				
	12.5	0	87.5						x					
	Functional group tolerance												Average yield	Total Scope
**Ref.**	**Halo.**	**Alco.**	**Amine**	**Amide**	**Cyano**	**Acid**	**Ester**	**Aldehyde**	**Phenol**	**aniline**	**Hetero.**	**Score FG (%)**	75.17	36
**206**	1	0	0	1	1	1	1	1	1	1	1	82		
	**Electronic compatibility**							**Versatility**						
	**Rich**	**Poor**	**Neut.**					**Rad.**	**Elect.**	**Nucleo.**				
	2.8	30.6	66.7						x					

Average scope: 22 or 2.2/10
Electronic compatibility: 2/5 or 4/10
Average yield: 54% or 5.4/10
Versatility: 2/6 or 3.3/10
Resource & health: Not toxic + price (0-50$/g) + not recyclable = 4/5 or 8/10
Functional group tolerance: 5.5/10
Average score: 4.7/10

$Bi_5O(OH)_9(NO_3)_4$ (R32)

	Functional group tolerance												Average yield	Total Scope
**Ref.**	**Halo.**	**Alco.**	**Amine**	**Amide**	**Cyano**	**Acid**	**Ester**	**Aldehyde**	**Phenol**	**aniline**	**Hetero.**	**Score FG (%)**	**59.49**	**30**
**174**	1	0	0	1	0	0	0	1	1	0	0	36		
	**Electronic compatibility**							**Versatility**						
	**Rich**	**Poor**	**Neut.**					**Rad.**	**Elect.**	**Nucleo.**				
	33.3	6.7	60.0						x					

Average scope: 30 or 3.0/10
Electronic compatibility: 2/5 or 4/10
Average yield: 59.5% or 5.9/10
Versatility: 2/6 or 3.3/10
Resource & health: Not toxic + price (0-50$/g) + not recyclable = 4/5 or 8/10
Functional group tolerance: 3.6/10

Average score: 4.7/10

Cerium ammonium nitrate (R41)

	Functional group tolerance												Average yield	Total Scope
**Ref.**	**Halo.**	**Alco.**	**Amine**	**Amide**	**Cyano**	**Acid**	**Ester**	**Aldehyde**	**Phenol**	**aniline**	**Hetero.**	**Score FG (%)**	**47.49**	**9**
**118**	0	0	1	0	0	0	1	0	0	0	1	27		
	**Electronic compatibility**							**Versatility**						
	**Rich**	**Poor**	**Neut.**					**Rad.**	**Elect.**	**Nucleo.**				
	22	0	77.8						x					

	Functional group tolerance												Average yield	Total Scope
**Ref.**	**Halo.**	**Alco.**	**Amine**	**Amide**	**Cyano**	**Acid**	**Ester**	**Aldehyde**	**Phenol**	**aniline**	**Hetero.**	**Score FG (%)**	48.48	27
**242**	1	0	0	1	0	0	0	0	0	0	0	18		
	**Electronic compatibility**							**Versatility**						
	**Rich**	**Poor**	**Neut.**					**Rad.**	**Elect.**	**Nucleo.**				
	0	0	100					x						

	Functional group tolerance												Average yield	Total Scope
**Ref.**	**Halo.**	**Alco.**	**Amine**	**Amide**	**Cyano**	**Acid**	**Ester**	**Aldehyde**	**Phenol**	**aniline**	**Hetero.**	**Score FG (%)**	65.77	31

241	1	0	0	0	1	0	1	0	0	0	0	27		
	**Electronic compatibility**							**Versatility**						
	**Rich**	**Poor**	**Neut.**					**Rad.**	**Elect.**	**Nucleo.**				
	19.4	29.0	51.6						x					
	**Functional group tolerance**												**Average yield**	**Total Scope**
**Ref.**	**Halo.**	**Alco.**	**Amine**	**Amide**	**Cyano**	**Acid**	**Ester**	**Aldehyde**	**Phenol**	**aniline**	**Hetero.**	**Score FG (%)**	41.5	18
218	1	0	0	1	1	0	1	0	0	0	1	45		
	**Electronic compatibility**							**Versatility**						
	**Rich**	**Poor**	**Neut.**					**Rad.**	**Elect.**	**Nucleo.**				
	0	0	100					x						

Average scope: 21.3 or 2.1/10
Electronic compatibility: 1/5 or 2/10
Average yield: 50.8% or 5.1/10
Versatility: 3/6 or 5/10
Resource & health: Not toxic + price (0-50$/g) + not recyclable = 4/5 or 8/10
Functional group tolerance: 2.9/10

Average score: 4.2/10

$Bi(NO_3)_3$ (R30)

	Functional group tolerance												Average yield	Total Scope
**Ref.**	**Halo.**	**Alco.**	**Amine**	**Amide**	**Cyano**	**Acid**	**Ester**	**Aldehyde**	**Phenol**	**aniline**	**Hetero.**	**Score FG (%)**	**85.95**	**21**
**207**	1	0	0	0	0	1	0	1	1	1	0	45		
	**Electronic compatibility**							**Versatility**						
	**Rich**	**Poor**	**Neut.**					**Rad.**	**Elect.**	**Nucleo.**				
	33.3	14.3	52.4						x					

	Functional group tolerance												Average yield	Total Scope
**Ref.**	**Halo.**	**Alco.**	**Amine**	**Amide**	**Cyano**	**Acid**	**Ester**	**Aldehyde**	**Phenol**	**aniline**	**Hetero.**	**Score FG (%)**	74.52	10
**208**	1	0	0	0	1	1	0	0	0	0	0	27		
	**Electronic compatibility**							**Versatility**						
	**Rich**	**Poor**	**Neut.**					**Rad.**	**Elect.**	**Nucleo.**				
	30	30	40						x					

Average scope: 15.5 or 1.6/10
Electronic compatibility: 3/5 or 6/10
Average yield: 80.2% or 8.1/10
Versatility: 2/6 or 3.3/10
Resource & health: Not toxic + price (0-50$/g) + not recyclable = 4/5 or 8/10
Functional group tolerance: 3.6/10
Average score: 5.1/10

$Ni(NO_3)_2.6H_2O$ (R42)

	Functional group tolerance												Average yield	Total Scope
**Ref.**	**Halo.**	**Alco.**	**Amine**	**Amide**	**Cyano**	**Acid**	**Ester**	**Aldehyde**	**Phenol**	**aniline**	**Hetero.**	**Score FG (%)**	**84.08**	**12**
**209**	1	0	0	0	0	0	0	1	1	0	0	27		
	**Electronic compatibility**							**Versatility**						
	**Rich**	**Poor**	**Neut.**					**Rad.**	**Elect.**	**Nucleo.**				
	36.4	8.3	8.3						x					

Average scope: 12 or 1.2/10
Electronic compatibility: 2/5 or 4/10
Average yield: 84.1% or 8.4/10
Versatility: 2/6 or 3.3/10
Resource & health: Toxic + price (0-50$/g) + not recyclable = 2/5 or 4/10
Functional group tolerance: 2.7/10

Average score: 3.9/10

Ethyl ammonium nitrate (R25)

	Functional group tolerance												Average yield	Total Scope
**Ref.**	**Halo.**	**Alco.**	**Amine**	**Amide**	**Cyano**	**Acid**	**Ester**	**Aldehyde**	**Phenol**	**aniline**	**Hetero.**	**Score FG (%)**	**83.05**	**33**
**175**	1	1	0	1	1	0	1	1	1	1	1	82		
	**Electronic compatibility**							**Versatility**						
	**Rich**	**Poor**	**Neut.**					**Rad.**	**Elect.**	**Nucleo.**				
	36.4	57.6	6.1						x					

Average scope: 33 or 3.3/10
Electronic compatibility: 4/5 or 8/10
Average yield: 83.1% or 8.3/10
Versatility: 2/6 or 3.3/10
Resource & health: Not toxic + price (0-50$/g) + not recyclable = 4/5 or 8/10
Functional group tolerance: 8.2/10

Average score: 4.8/10

3-methyl-1-sulfonic acid imidazolium nitrate (R37)

	Functional group tolerance												Average yield	Total Scope
**Ref.**	**Halo.**	**Alco.**	**Amine**	**Amide**	**Cyano**	**Acid**	**Ester**	**Aldehyde**	**Phenol**	**aniline**	**Hetero.**	**Score FG (%)**	**74.53**	**17**
**210**	1	0	0	0	0	0	0	0	1	1	0	27		
	**Electronic compatibility**							**Versatility**						
	**Rich**	**Poor**	**Neut.**					**Rad.**	**Elect.**	**Nucleo.**				
	64.7	0	35.3					x						

Average scope: 17 or 2.6/10
Electronic compatibility: 2/5 or 4/10
Average yield: 74.5% or 7.5/10
Versatility: 2/6 or 3.3/10
Resource & health: Unknown + price (>200$/g) + not recyclable = 1/5 or 2/10
Functional group tolerance: 2.7/10

Average score: 3.5/10

Thiourea Nitrate (R39)

	Functional group tolerance												Average yield	Total Scope
**Ref.**	**Halo.**	**Alco.**	**Amine**	**Amide**	**Cyano**	**Acid**	**Ester**	**Aldehyde**	**Phenol**	**aniline**	**Hetero.**	**Score FG (%)**	**53.92**	**12**
**211**	0	0	0	0	1	1	0	1	1	1	0	45		
	**Electronic compatibility**							**Versatility**						
	**Rich**	**Poor**	**Neut.**					**Rad.**	**Elect.**	**Nucleo.**				
	25.0	0	75.0						x					

Average scope: 12 or 1.2/10
Electronic compatibility: 1/5 or 2/10
Average yield: 53.9% or 5.4/10
Versatility: 2/6 or 3.3/10
Resource & health: Toxic + price (0-50$/g) + not recyclable = 2/5 or 4/10
Functional group tolerance: 4.6/10

Average score: 3.4/10

Pyridinium Nitrate (R38)

	Functional group tolerance											Average yield	Total Scope
**Ref.**	**Halo.**	**Alco.**	**Amine**	**Amide**	**Cyano**	**Acid**	**Ester**	**Aldehyde**	**Phenol**	**aniline**	**Hetero.**	**Score FG (%)**	**72.41**	**17**
**243**	1	0	0	0	0	0	0	0	1	1	0	27		
	**Electronic compatibility**							**Versatility**						
	**Rich**	**Poor**	**Neut.**					**Rad.**	**Elect.**	**Nucleo.**				
	58.8	0	16.2					x						

Average scope: 17 or 1.7/10
Electronic compatibility: 2/5 or 4/10
Average yield: 72.4% or 7.2/10
Versatility: 2/6 or 3.3/10
Resource & health: Unknown + price (>200$/g) + not recyclable = 1/5 or 2/10
Functional group tolerance: 2.7/10

Average score: 3.5/10

$Y(NO_3)_3.6H_2O$ (R40)

	Functional group tolerance												Average yield	Total Scope
**Ref.**	**Halo.**	**Alco.**	**Amine**	**Amide**	**Cyano**	**Acid**	**Ester**	**Aldehyde**	**Phenol**	**aniline**	**Hetero.**	**Score FG (%)**	**82.0**	**6**
**212**	0	0	0	0	0	0	1	1	1	0	0	27		
	**Electronic compatibility**							**Versatility**						
	**Rich**	**Poor**	**Neut.**					**Rad.**	**Elect.**	**Nucleo.**				
	83.3	0	16.7						x					

Average scope: 6 or 0.6/10
Electronic compatibility: 2/5 or 4/10
Average yield: 82.0% or 8.2/10
Versatility: 2/6 or 3.3/10
Resource & health: Not toxic + price (0-50$/g) + not recyclable = 4/5 or 8/10
Functional group tolerance: 2.7/10

Average score: 2.7/10

Nitro methane (R36)

	Functional group tolerance												Average yield	Total Scope
**Ref.**	**Halo.**	**Alco.**	**Amine**	**Amide**	**Cyano**	**Acid**	**Ester**	**Aldehyde**	**Phenol**	**aniline**	**Hetero.**	**Score FG (%)**	**54.48**	**29**
**176**	1	0	0	0	0	0	1	0	1	1	0	36		
	**Electronic compatibility**							**Versatility**						
	**Rich**	**Poor**	**Neut.**					**Rad.**	**Elect.**	**Nucleo.**				
	51.7	6.9	41.4						x					

Average scope: 29 or 2.9/10
Electronic compatibility: 2/5 or 4/10
Average yield: 54.5% or 5.5/10
Versatility: 2/6 or 3.3/10
Resource & health: Toxic + price (0-50$/g) + not recyclable = 2/5 or 4/10
Functional group tolerance: 3.6/10

Average score: 3.9/10

3-(Ethoxycarbonyl)-1-(5-methyl-5-(nitrosooxy)hexyl)pyridin-1-ium bis(trifluoromethanesulfonyl)imides (R27)

	Functional group tolerance												Average yield	Total Scope
**Ref.**	**Halo.**	**Alco.**	**Amine**	**Amide**	**Cyano**	**Acid**	**Ester**	**Aldehyde**	**Phenol**	**aniline**	**Hetero.**	**Score FG (%)**	**75.79**	**30**
**245**	1	0	0	1	1	1	1	0	1	1	0	64		
	**Electronic compatibility**							**Versatility**						
	**Rich**	**Poor**	**Neut.**					**Rad.**	**Elect.**	**Nucleo.**				
	46.7	20.0	33.3						x					

Average scope: 30 or 3.0/10
Electronic compatibility: 3/5 or 6/10
Average yield: 75.8% or 7.6/10
Versatility: 2/6 or 3.3/10
Resource & health: Unknown + price (>200$/g) + not recyclable = 1/5 or 2/10
Functional group tolerance: 6.4/10

Average score: 4.7/10

5-methyl-1,4-dinitroimidazole (R26)

	Functional group tolerance												Average yield	Total Scope
Ref.	Halo.	Alco.	Amine	Amide	Cyano	Acid	Ester	Aldehyde	Phenol	aniline	Hetero.	Score FG (%)	**66.36**	**14**
**178**	1	0	1	0	0	0	1	0	1	0	0	36		
	**Electronic compatibility**							**Versatility**						
	**Rich**	**Poor**	**Neut.**					**Rad.**	**Elect.**	**Nucleo.**				
	85.7	0	14.3					x						

	Functional group tolerance												Average yield	Total Scope
Ref.	Halo.	Alco.	Amine	Amide	Cyano	Acid	Ester	Aldehyde	Phenol	aniline	Hetero.	Score FG (%)	66.46	98
**148**	1	0	0	1	0	0	1	1	1	1	1	64		
	**Electronic compatibility**							**Versatility**						
	**Rich**	**Poor**	**Neut.**					**Rad.**	**Elect.**	**Nucleo.**				
	31.6	20.4	48						x					

	Functional group tolerance												Average yield	Total Scope
Ref.	Halo.	Alco.	Amine	Amide	Cyano	Acid	Ester	Aldehyde	Phenol	aniline	Hetero.	Score FG (%)	73.03	36

**179**	1	0	0	0	1	0	1	0	1	1	0	45		
	**Electronic compatibility**							**Versatility**						
	**Rich**	**Poor**	**Neut.**					**Rad.**	**Elect.**	**Nucleo.**				
	44.4	30.6	25.0					x						

Average scope: 49.3 or 4.9/10
Electronic compatibility: 3/5 or 6/10
Average yield: 68.6% or 6.9/10
Versatility: 3/6 or 5/10
Resource & health: Unknown + price (>200$/g) + Recyclable = 2/5 or 4/10
Functional group tolerance: 4.9/10

Average score: 5.3/10

2-methoxymethyl nitrite (R33)

	Functional group tolerance												Average yield	Total Scope
**Ref.**	**Halo.**	**Alco.**	**Amine**	**Amide**	**Cyano**	**Acid**	**Ester**	**Aldehyde**	**Phenol**	**aniline**	**Hetero.**	**Score FG (%)**	**79.14**	**22**
**244**	1	1	1	0	0	0	1	1	1	0	0	55		
	**Electronic compatibility**							**Versatility**						
	**Rich**	**Poor**	**Neut.**					**Rad.**	**Elect.**	**Nucleo.**				
	90.9	9.1	0					x						

Average scope: 22 or 2.2/10
Electronic compatibility: 2/5 or 4/10
Average yield: 79.1% or 7.9/10
Versatility: 2/6 or 3.3/10
Resource & health: Unknown + price (>200$/g) + not recyclable = 1/5 or 2/10
Functional group tolerance: 5.5/10

Average score: 4.2/10

Dinitro-5,5-dimethylhydantoin (R34)

	Functional group tolerance												Average yield	Total Scope
**Ref.**	**Halo.**	**Alco.**	**Amine**	**Amide**	**Cyano**	**Acid**	**Ester**	**Aldehyde**	**Phenol**	**aniline**	**Hetero.**	**Score FG (%)**	**61.94**	**32**
**213**	1	0	0	0	1	0	0	1	1	0	1	45		
	**Electronic compatibility**							**Versatility**						
	**Rich**	**Poor**	**Neut.**					**Rad.**	**Elect.**	**Nucleo.**				
	40.6	9.4	50						x					

Average scope: 32 or 3.2/10
Electronic compatibility: 3/5 or 6/10
Average yield: 61.9% or 6.2/10
Versatility: 2/6 or 3.3/10
Resource & health: Unknown + price (>200$/g) + not recyclable = 1/5 or 2/10
Functional group tolerance: 4.6/10

Average score: 4.2/10

Benziodazole-Type O_2NO-I(III) (R35)

	Functional group tolerance												Average yield	Total Scope
**Ref.**	**Halo.**	**Alco.**	**Amine**	**Amide**	**Cyano**	**Acid**	**Ester**	**Aldehyde**	**Phenol**	**aniline**	**Hetero.**	**Score FG (%)**	**74.51**	**35**
**246**	1	0	0	1	0	0	1	0	1	1	0	45		
	**Electronic compatibility**							**Versatility**						
	**Rich**	**Poor**	**Neut.**					**Rad.**	**Elect.**	**Nucleo.**				
	62.9	0	37.1					x						

Average scope: 35 or 3.5/10
Electronic compatibility: 2/5 or 4/10
Average yield: 74.5% or 7.5/10
Versatility: 2/6 or 3.3/10
Resource & health: Unknown + price (>200$/g) + not recyclable = 1/5 or 2/10
Functional group tolerance: 4.6/10

Average score: 4.1/10

II. Extended analysis for the aromatic nitration:

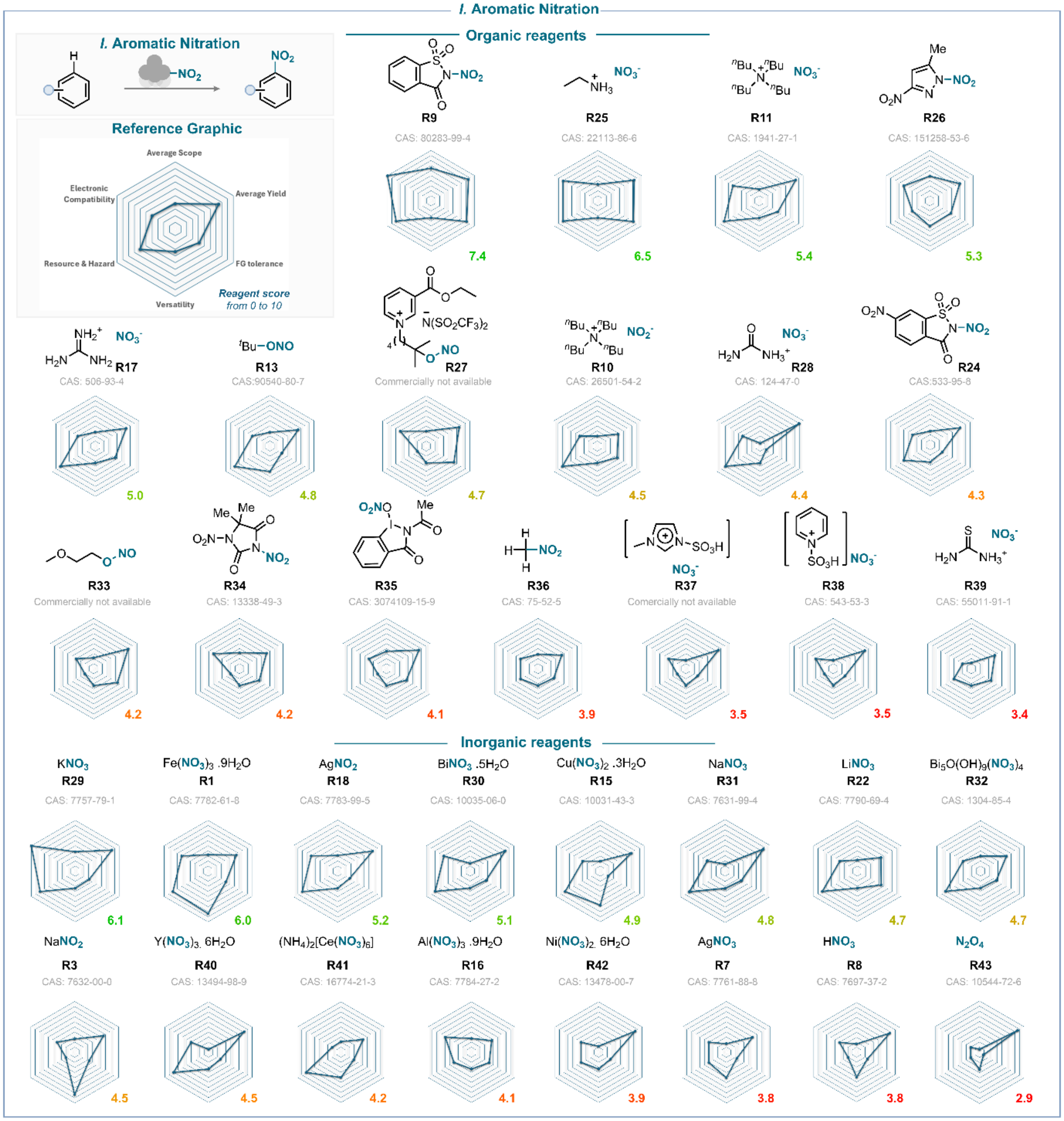

Supplement: Supplementary file 1 — Supporting File 1: anie71946‐sup‐0001‐SuppMat.pdf. [file ANIE-65-e26128-s001.pdf]
